# Community engagement for stakeholder and community trust in healthcare: Short-term evaluation findings from a nationwide initiative in Lao PDR

**DOI:** 10.1016/j.socscimed.2024.117079

**Published:** 2024-08

**Authors:** Marco J. Haenssgen, Elizabeth M. Elliott, Sysavanh Phommachanh, Ounkham Souksavanh, Hironori Okabayashi, Shogo Kubota

**Affiliations:** a Department of Social Science and Development, Chiang Mai University, 239 Huay Kaew Rd. T. Suthep Muang, Chiang Mai, 50200, Thailand; b World Health Organization Regional Office for the Western Pacific, P.O. Box 2932, 1000, Manila, Philippines; c Institute of Research and Education Development, University of Health Sciences, Payawat Village, Sisattanak District, Vientiane Capital, Laos; d World Health Organization Representative, Country Office for Lao People's Democratic Republic, 125 Saphanthong Road, Unit 5 Ban Saphangthongtai, Sisattanak District, Vientiane Capital, Laos

**Keywords:** Trust, Health system, Mixed methods, Community engagement, Rural areas, Lao PDR

## Abstract

**Background:**

Trust remains a critical concept in healthcare provision, but little is known about the ability of health policy and interventions to stimulate more trusting relationships between communities and the health system. The CONNECT (Community Network Engagement for Essential Healthcare and COVID-19 Responses Through Trust) Initiative in Lao PDR provided an opportunity to assess the community-level impact of a trust-building community engagement approach.

**Methods:**

A mixed-method process evaluation was implemented from 10/2022–12/2023 among 14 diverse case study communities in four provinces across Lao PDR. Data collection involved two rounds of census surveys (3161 observations incl. panel data from 618 individuals) including an 8-item trust scale, 50 semi-structured interviews with villagers, and 50 contextualizing key informant interviews. The two data collection rounds were implemented before and three months after village-based CONNECT activities and helped discern impacts among activity participants, indirectly exposed villagers, and unexposed villagers in a difference-in-difference analysis.

**Results:**

Stakeholders attested strong support for the CONNECT Initiative although community-level retention of trust-related themes from the activities was limited. Quantitative data nevertheless showed that, at endline, the 8-item trust index (from [–8 to +8]) increased by 0.95 points from 4.44 to 5.39 and all trust indicators were universally higher. Difference-in-difference analysis showed that villagers exposed to the CONNECT activities had a 1.02-index-point higher trust index compared to unexposed villagers. Trust impacts improved gradually over time and were relatively more pronounced among men and ethnic minority groups.

**Conclusions:**

The CONNECT Initiative had considerable direct and systemic effects on community members’ trust in their local health centers in the short term, which arose from strong stakeholder mobilization and gradual institutional learning. Relational community engagement approaches have the potential to create important synergies in health policy and broader cross-sectorial strategies, but also require contextual grounding to identify locally relevant dimensions of trust.

## Highlights

• Community engagement holds promise for building trust in healthcare
• Study provides short-term mixed-method evaluation evidence from rural Lao PDR
• Trust improved strongly and consistently across all dimensions
• Systemic and direct impacts underline success of relational community engagement
• Trust-building approaches require strong stakeholder support and local grounding

## 1 Introduction

It is widely accepted – in policy, practice, and scholarship alike – that “Trust matters in health care” (Lee et al., 2019:539) – not only intrinsically but also instrumentally for health service utilization, patient satisfaction, subjective health outcomes, and even lower mortality (Birkhäuer et al., 2017; Ezumah et al., 2022; Halbert et al., 2009). The COVID-19 pandemic amplified the interest in the subject yet further due to concerns about globally waning population trust in healthcare and science (Taylor et al., 2023).

While proposals on how to build trust are plentiful, actual evidence on their effectiveness is largely absent or otherwise mixed and concentrated in high-income settings. Community engagement – generally understood as both a process and outcome of community-led multi-stakeholder cooperation to address local health-related issues (WHO, 2017, 2020) – offers an alternative route to building trust that is explicitly relational and respectful of the agency of target populations, without imposing external knowledge and solutions in a top-down fashion (De Weger et al., 2018; Kilpatrick, 2009; Milton et al., 2011; Odugleh-Kolev and Parrish-Sprowl, 2018). Relational community engagement approaches have also expanded rapidly into low- and middle-income settings during the pandemic (Loewenson et al., 2021), but evaluation knowledge on their impacts on trust in healthcare remains scarce as well.

Speaking to this important research gap in global health policy and practice, the objective of this article is to contribute to the understanding of whether and how relational community engagement activities can build stakeholder and community trust in healthcare. We draw on the case of a nationwide initiative to strengthen primary health care services that the Lao PDR Ministry of Health (MoH) and Ministry of Home Affairs (MoHA) implemented with technical support from the World Health Organization (WHO). This initiative was borne out of healthcare challenges experienced during and prior to the COVID-19 pandemic and involved support for localised healthcare governance and a multi-sectoral approach to empower communities to jointly improve health of the people in Lao PDR (WHO WPRO, 2023). Relationship building and healthcare ownership through trust-building engagement activities on the village level played a central role in this process; the initiative became accordingly referred to as Community Network Engagement for Essential Healthcare and COVID-19 Responses Through Trust (CONNECT).

## 2 Background

### 2.1 Building trust in healthcare through community engagement

While the notion of trust in vernacular and policy discourses often remains ambiguous with a wide range of meanings (Taylor et al., 2023), it is commonly understood in the academic literature as a relational and multidimensional concept that relates to issues of power, risk, and ethics (Larson et al., 2018; Pilgrim and Vassilev, 2010; Ward, 2017). In the domain of “patient trust in healthcare,” trust can be understood as “a set of expectations that the healthcare provider will do the best for the patient, and with good will, recognising the patient's vulnerability” (Rasiah et al., 2020:2), and can be divided further into several types and dimensions. Three major types of trust in healthcare include interpersonal forms such as honesty and respect, service- and product-related trust (e.g. perceptions of vaccine safety or provider competence), and institutional trust for instance in the government's ability to operate a functioning health system (Adhikari et al., 2022; Gopichandran and Chetlapalli, 2013; Larson et al., 2018; LoCurto and Berg, 2016). Note also that not only patients should be expected to trust healthcare providers but also providers themselves need to build trust and respect towards patients (Sousa-Duarte et al., 2020).

Patterns of trust in a population are subject to generalised trust on a societal level, external influencing factors such as health information or political action, and to individual and shared experiences and background characteristics that are shaped for instance by ethnicity, gender, or wealth (Larson et al., 2018; Murray and McCrone, 2015). However, the literature is dominated by high-income perspectives (Ozawa and Sripad, 2013; Taylor et al., 2023), which creates problematic blind spots as different health system contexts can produce qualitatively different encounters between patients and healthcare providers (Gopichandran et al., 2015). In low- and middle-income country contexts, the emerging yet still scant literature has for instance highlighted different expressions of interpersonal trust as loyalty (Gopichandran and Chetlapalli, 2013), demonstrated how healthcare providers built interpersonal trust through more salient (albeit not necessarily qualitatively better) performances of care (Rodrigues, 2021), and emphasized the different patterns of trust towards diverse healthcare providers in pluralistic health systems (Gryseels et al., 2019; Ozawa and Walker, 2011). In research from Lao PDR, interpersonal trust was a particularly pronounced type of trust in healthcare as patients depended strongly on existing social relationships with healthcare providers or other community members’ recommendations for trustworthy resorts to care (Bochaton, 2015; Buchner, 2011; Elliott, 2021; Phommachanh et al., 2019a, 2019b; Sychareun et al., 2012). Low- and middle-income country experiences can thus help develop our understanding of the nuanced and contextualised expressions of patient trust in healthcare.

Overall, the academic literature accepts the importance of trust in healthcare utilization (Lee et al., 2019). However, how concretely patient trust in healthcare can be built remains an important research gap. Among the wide range of suggested activities are, for instance, top-down or provider-initiated communication and education campaigns to persuade patients that healthcare providers and solutions such as vaccines are trustworthy, patient satisfaction enquiries, or provider-sided training programmes to engage in more respectful and empathetic interactions with patients to earn their trust (Lee et al., 2019; Mechanic, 1996; Murray and McCrone, 2015; Rolfe et al., 2014). Approaches addressing institutional trust could also be considered given that pressures of commercialisation and privatisation can undermine institutional forms of trust, for instance through healthcare staff disclosure of financial interests, visible standards of practice, or the inclusion of patient representatives in the boards or medical institutions (Lee et al., 2019; Mechanic, 1996; Rolfe et al., 2014). Evidence on the success of such measures has again been heavily biased towards high-income contexts and showed at best small improvements in patient trust (Brennan et al., 2013; Rolfe et al., 2014). For example, a systematic review of interventions targeting medical doctors by Rolfe et al. (2014) found that trials involving communication training and the disclosure of financial interests improved patient trust only in some isolated cases.

A particularly promising yet still understudied route to building trust in healthcare is community engagement – an approach that experienced a surge in interest during the COVID-19 pandemic (Adebisi et al., 2021; Gilmore et al., 2020; Questa et al., 2020). Community engagement is an approach that spans a wide spectrum of participation, empowerment, and community control: from “informing” communities at one end of the spectrum via co-production at the centre and to maximising their agency and control over health matters at the other end of the spectrum (De Weger et al., 2018; Kilpatrick, 2009; Milton et al., 2011). While community engagement generally emphasises relationships, trust, and mutual work between communities and other actors such as healthcare providers or governments on eye-level, the emphasis on “relational” aspects is a recent evolution of the field that (a) encourages context-sensitive health service provision by foregrounding people's viewpoints of health priorities and solutions, (b) recognizes humans as intrinsically social beings (i.e. expanding away from individualistic models of behavior and the primacy of physical health), and (c) considers dynamically evolving relationships between health service providers and the local population (Odugleh-Kolev and Parrish-Sprowl, 2018; WHO, 2017, 2020; WHO WPRO, 2023). The relational approach received rapid recognition during the pandemic to encourage participation in COVID-19 prevention and control measures (Loewenson et al., 2021), but its impacts specifically on building trust have remained elusive. The case of the CONNECT Initiative, which aimed at building trust in healthcare especially in rural Lao communities, offers an opportunity to speak to this important research gap.

### 2.2 The CONNECT initiative

The CONNECT Initiative in Lao PDR responded to challenges surrounding community trust in healthcare that became visible during the COVID-19 pandemic (WHO WPRO, 2023). Among others, the pandemic years demonstrated highly variable vaccination uptake, delays in testing, and access to primary healthcare services – not only across socio-economic strata but also as a systemic result of poor trust in state service provision (Sychareun et al., 2022; Wilcox, 2022). Responding thus to a need for locally led healthcare provision, relationship building, and community ownership in healthcare, the format of the CONNECT Initiative reflected the character of a relational community engagement initiative (emphasizing elements of “delegated power” and “community control” in the community engagement typology; Milton et al., 2011). It was co-designed as an approach (rather than a “project”) to empower citizens and local officials to seek health solutions based on local knowledge and capacity that are realized together for their community through building trust (WHO WPRO, 2023). The Initiative collaborated with local officials through the Lao PDR MoH and MoHA (in collaboration with WHO) to prepare for, prevent, and respond to the potential widespread transmission of COVID-19 and enhance community health beyond COVID-19 to achieve Universal Health Coverage and health-related Sustainable Development Goals – all against the backdrop of achieving health equity in Lao PDR. The roll-out of CONNECT commenced in December 2021 (and was still ongoing at the time of this manuscript), targeting high priority “focus” districts and villages nation-wide.

As can be seen in Table 1, the CONNECT activities comprised three modules that together aimed to achieve a set of five interlinked operational objectives. Objective 3.3 (see bottom row in Table 1) was of particular interest for the current study as it focused on “Enhanced trust” among community members and primary healthcare providers. This objective reflected the key mechanism underlying CONNECT's theory of change, which the Initiative intended to realize especially through its Module-2 activities. The Module-2 activities took place on the community level and involved (a) a participatory planning workshop with community members, health staff, district and provincial stakeholders, and central-level facilitators to understand needs and available resources of villagers and jointly develop a village plan; and (b) supportive supervision activities to support communities' implementation, scale-up, and institutionalization of village-based activities through central and provincial teams, health centers, and village representatives. Trust-building opportunities within the Module 2 workshop involved, among others, collaborative tasks such as a “health bridge” building competition, participatory mapping to understand the local community, scenario role plays to demonstrate values and communication practices, and the joint formulation of health-related community action plans based on a growing mutual understanding between villagers and healthcare staff. The supportive supervision activities subsequently reinforced these workshop activities and contributed to the ongoing building of trust through mentoring and role plays.

**Table 1:** Overview of CONNECT Initiative activities.

	Module 1	Module 2 (*focal module in this study*)	Module 3
**Focus**	Strengthened local health governance in alignment with *Samsang* (“Three Builds Policy”) to respond to COVID-19 and primary health care challenges	Empowered and resilient communities to collectively and effectively respond to COVID-19 and primary health care challenges in their community	Strengthened health professionals' capacities on COVID-19 and essential maternal and child health care services through a respectful and people-centered approach
**Target**	Health service governance: administrative and health authorities from village, district, and provincial levels	(Mainly) rural communities and frontline public health service providers: village representatives, villagers (esp. “vulnerable” members incl. pregnant women and women in child-bearing age), and health center staff	Frontline public health service providers: health center staff
**Activities**	2-day interactive health governance strengthening planning workshop Funding mobilization for district- and province-wide CONNECT implementation	**Workshop:** 2-day facilitator training; 2-day participatory planning workshop with community members including health staff to identify existing resources, build relationships, develop plans; 1-day lessons learned meeting with facilitators **Supportive supervision:** Central & provincial teams support district officials and health centre staff to support village representatives and community members to implement community action plans, as well as scaling up activities and good practices to other villages until these become integrated into routine mechanisms.	3-day healthcare staff training on people-centered care for the safe provision of essential MCH services during COVID-19
**Topics**	Locally led, government-funded scalability and institutionalization, universal (primary) healthcare access, COVID-19, essential healthcare services	Ownership of and agency in promoting health, healthcare access, relationship between healthcare providers and communities, COVID-19, essential healthcare services	Maternal and child healthcare services, COVID-19, counselling skills, respectful care, referral care
**Objectives**	3.1: Strengthened central/local health governance	3.2: Enhanced community engagement 3.3: Enhanced trust 3.4: Increased service utilization	3.5: Improved healthcare service quality

## 3 Material and methods

### 3.1 Research design

Embedded within a larger multi-sector process evaluation of the CONNECT Initiative (involving policy, health sector, and community-level assessments; Phrasisombath et al., 2024), this study reports on the subset of the village-based evaluation activities concerned specifically with enhancing community trust in primary healthcare providers (CONNECT Objective 3.3). To assess the community-level effects of the Initiative, we implemented a two-round mixed-method evaluation design that involved a set of methods summarized in Table 2.

**Table 2:** Summary of research methods.

	Community survey	Semi-structured interviews	Expert interviews
**Objective**	To assess patterns and scale of community-level effects of the CONNECT Initiative	To test survey questionnaire and contextualize survey responses	To provide complementary insights into the implementation and perception of the CONNECT Initiative
**Data collection**	40-min interviewer-administered face-to-face questionnaire	20-30-min semi-structured community member interviews plus survey implementation fieldnotes	30-45-min semi-structured key informant interviews
	Two rounds of data collection: baseline plus endline (endline at least three months after CONNECT implementation)		
**Population**	Adults aged ≥16 years in 14 diverse case study communities across four provinces where CONNECT took place		Healthcare stakeholders from local to central government levels, village representatives, villagers
**Sampling technique**	Complete census surveys in all 14 case study communities	Purposive maximum variation sampling (sex, age, education, ethnicity, community), recruited from survey sample	Purposive sampling (competencies and experiences relating to the CONNECT Initiative)
**Sample size**	3161	50	50
**Analysis techniques**	Descriptive statistical analysis, difference-in-difference analysis	Content-oriented thematic analysis	

The assessment of trust followed previous mixed-method research in which we established the relevance of community members’ trust in their primary healthcare providers and identified relevant of the concept in rural Lao PDR (see Section 3.3 for the operationalisation of “trust” in the survey instruments) (Haenssgen et al., 2024). Building on this conceptual groundwork, the two-round census survey design permitted us to trace changes of multi-dimensional trust among participants and non-participants of the CONNECT activity, taking into account the complete social composition of the participating communities, and thus community-level changes. The quantitative survey data further enabled difference-in-difference analyses of relative changes among directly and indirectly exposed participants (i.e. those aware of CONNECT but who did not participate in its activities), and non-participants as a “control group” within the community setting. Qualitative methods contextualised and validated the quasi-experimental quantitative analysis results. The research was implemented between October 2022 and December 2023.

### 3.2 Ethical approval

We received ethical approval from the Lao National Ethics Committee for Health Research (NECHR; ref. 069/NECHR). Prior informed consent was obtained from all study participants. The data collection in was integrated into government structures: public health authorities facilitated village access and data collection; operational insights from each survey mission were fed back to village and health authorities; and survey findings were shared with provincial and central government authorities (including MoH and MoHA). Participants received a small in-kind gift as a token of appreciation for their time and contribution.

### 3.3 Research sites

**Fig. 1:**
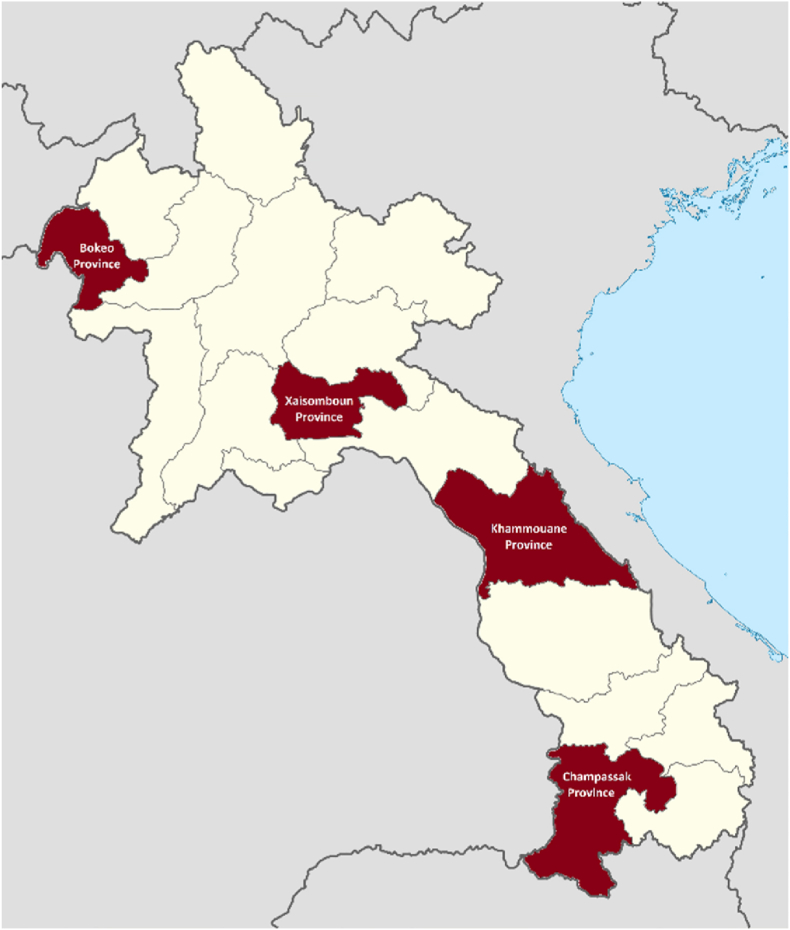
Map of Lao PDR and study sites. *Notes*. Study provinces highlighted in red; Bokeo province was also site for the preliminary qualitative research.

Source: Adapted from Wikimedia Commons (2023), file: Laos provinces.svg. user: Infernoapple.

At the time of this research, Lao PDR was classified as a lower-middle income country, which had undergone a prolonged period of rapid economic development but experienced persistent healthcare challenges and inequalities, which were further complicated by the COVID-19 pandemic and the subsequent post-COVID geopolitical environment (Kim et al., 2024; World Bank, 2023). The specific study provinces of Bokeo, Xaisomboun, Khammouane, and Champassak (depicted in Fig. 1) reflected the variability of development outcomes across the country, with poverty rates and adult literacy in Bokeo of 25.5% and 67.0%, in Xaisomboun of 27.8% and 74.4%, in Khammouane of 27.1% and 83.5%, and in Champassak of 22.8% and 91.2% (Coulombe et al., 2016). Three to four case study communities were selected within each province as study sites, all of which participated in the CONNECT Initiative and were selected purposively from CONNECT's participant registers. The communities reflected geographical, economic, and ethnic diversity (see Supplementary Material Table A1 for details). For example, reported literacy rates in the four study sites in Bokeo ranged from 58.3% to 80.6% and from 34.9% to 67.0% in the three study communities in Khammouane. Similarly, whereas up to 94.7% of the village population belonged to the majority Lowland Lao Loum ethnicity in a study community in Champassak Province, some of the Hmong ethnic communities in Xaisomboun Province had less than 2.0% Lowland Lao Loum residents.

### 3.4 Data and analysis

Emulating the survey implementation process described in Haenssgen et al. (2018), quantitative survey data from the case study villages was collected in a two-round census survey design, wherein all available members of the 14 case study communities (aged 16 and above) were invited to participate in a baseline survey and a follow-up survey at least three months after the first CONNECT activities (attendance at workshops and supportive supervision was recorded separately). Because village population registers often varied in their accuracy, we established sampling frames of each selected community based on publicly available satellite maps (*Google Maps* and *Bing Maps*), on which we identified and numbered all residential structures, and subsequently approached them in person during the community visits to enumerate and invite all available adult residents to our survey (Haenssgen, 2015). Where satellite maps did not cover the most recent housing construction in a community, we added them to the sampling frame on-the-fly.

The 40-min questionnaire used in the survey covered several modules to evaluate the village-based CONNECT activities (e.g. respondent/household characteristics, or COVID-19-related attitudes and practices). Relevant for this study were modules covering the socio-economic background of the respondents, endline information on their participation in the CONNECT Module-2 activities, and a module collected at baseline and endline about respondents’ trust in primary care services. Building on the preliminary qualitative work to conceptualize trust (Haenssgen et al., 2024), this module elicited questions to compose an 8-item trust index to gauge attitudes towards primary healthcare services. The eight dimensions corresponded to three major types of trust in healthcare (Adhikari et al., 2022; Larson et al., 2018), namely interpersonal trust (5 dimensions: respectful care, integrity, communication, relationship, fairness), service-related trust (2 dimensions: assurance of treatment, perceived competence), and institutional trust (1 dimension: reputation of healthcare provider, gauged through positive or negative tonality of “stories” surrounding health centers in the community). The module further allowed participants to provide an overarching judgement whether they would “rather trust or distrust” their health center, and it triangulated the trust index with a recommendation index comprising their recommended resort to care for (a) antenatal care, (b) place of delivery, (c) injury treatment, and (d) COVID-19 care (Haenssgen et al., 2024).

The qualitative material had two main sources (Haenssgen, 2020). Firstly, semi-structured 20-30-min interviews accompanied the survey to interpret and contextualize the survey findings, involving community members who were recruited purposively with view towards maximum variation (sex, age, education, ethnicity, community) from among the survey respondents. The semi-structured interview guide followed key questionnaire items, thus allowing respondents to add further contextualizing and in-depth information about the various dimensions of trust and their experiences with healthcare services. Qualitative survey implementation notes collected by the survey team complemented these interviews. Secondly, 30-45-min semi-structured key informant interviews with community members and representatives, village authorities, health center staff as well as CONNECT stakeholders on the district, provincial, and central levels helped provide further information related to the policy context and higher-level implementation of the CONNECT Initiative. The respondents were again recruited purposively based on their competencies and experiences relating to the CONNECT Initiative across all study provinces and implementation levels. The key informant interview guide followed the evaluation criteria that guided the CONNECT evaluation to provide information about efficiency, effectiveness, relevance, coherence, impact, sustainability, and equity (Haenssgen, 2019; OECD Development Assistance Committee, 2019). The topic of “trust” was thereby particularly relevant to the evaluation criteria of “relevance” (as key mechanism underlying the theory of change of CONNECT) and “effectiveness” in terms of achievement of the CONNECT Objective 3.3.

A 7-member Lao team experienced in community development and who received five days of mixed-method evaluation and survey training implemented the data collection. The questionnaire and interview guides (available in the Supplemental Material) were developed in Lao language with the help of the survey team. The survey data collection used an interviewer-administered questionnaire operating on tablets using *SurveyCTO* (Dobility Inc., 2017). A survey pilot took place in Champassak Province and was followed by a further round of expert review (including CONNECT implementation team, survey and community development specialists, and maternal and child health experts), which entailed minor revisions of the questionnaire (the data remained comparable across all provinces). Interview data was collected through face-to-face interviews, which were audio-recorded, transcribed, and translated into English by the evaluation team. All survey and qualitative data were collected in Lao or in the preferred ethnic languages of the respondents (for which we recruited local translators), and participants received a small in-kind gift as a token of appreciation for their time and participation.

The resulting material (summarized in Table 3) comprised 54:02hrs of audio-recorded material equivalent to 209,000 words of transcription and translation from 50 cognitive interviews and 50 key informant interviews. 48.0% of all qualitative participants were female and the subset of interviews involving villagers and village authorities (71 out 100 interviews) included a relative majority of 28.2% lowland Lao (Lao Loum) ethnicity, Hmong (25.4%), and Khmu (19.7%) among others. The survey data (summarized in Table 4) included 3161 survey observations (1838 at baseline and 1323 at endline, whereby endline data collection was complicated by seasonal working and migration patterns), of whom 53.0% were female and whose main ethnic groups included Lao Loum (35.4%), Hmong (20.1%), and Khmu (18.3%). In addition, 618 respondents could be followed up and matched across the two survey rounds, which enabled us to create a 2-round panel data sets from a sub-sample of the survey.

**Table 3:** Summary of qualitative data.

	Province				Central level	Total
	Champassak	Bokeo	Khammouane	Xaisomboun		
Semi-Structured Interviews (SSI)	11	11	11	17		50
*Baseline*	*5*	*4*	*7*	*10*		*26*
*Endline*	*6*	*7*	*4*	*7*		*24*
						
Key Informant Interviews (KII)	9	12	12	13	4	50
*Baseline*	*3*	*5*	*5*	*4*	*4*	*21*
*Endline*	*6*	*7*	*7*	*9*		*29*
						
**Total**	**20**	**23**	**23**	**30**	**4**	**100**

**Table 4:** Summary statistics.

Variable		Explanation	Baseline					Endline					Total				
			Mean	Std. dev.	Min.	Max.	N	Mean	Std. dev.	Min.	Max.	N	Mean	Std. dev.	Min.	Max.	N
Gender^a^		% female	51.6%				1838	54.9%				1323	53.0%				3161
		% male	48.4%					45.1%					47.0%				
Age (in years)		Age as reported by respondent	40.44	15.37	16	87	1838	40.51	15.49	16	91	1323	40.47	15.42	16	91	3161
Education (in years)		Years of completed education	5.23	4.03	0	20	1838	5.24	4.12	0	20	1123	5.23	4.06	0	20	2961
Ethnic group		% majority ethnic group (Lowland Lao)	37.9%				1838	26.3%				1323	33.0%				3161
		% minority ethnic group	62.1%					73.7%					67.0%				
Disability		% no self-declared physical disability	97.4%				1838	97.7%				1323	97.6%				3161
		% self-declared physical disability	2.6%					2.3%					2.4%				
Wealth [0 to +1]		Aggregate index of 8 common household assets	0.50	0.21	0	0.88	1817	0.47	0.21	0	0.88	1108	0.49	0.21	0	0.88	2925
Household size		Number of household members	6.44	2.89	0	19	1824	6.64	2.90	1	23	1111	6.52	2.89	0	23	2935
**Exposure to CONNECT activities**		Heard about workshop or supportive supervision	.	.	.	.	0	0.37	0.48	0	1	1323	0.37	0.48	0	1	1323
		Attended workshop or supportive supervision	.	.	.	.	0	0.29	0.45	0	1	1323	0.29	0.45	0	1	1323
**Trust dimensions [-1 to +1]**^b^	Poor treatment	Indicator of interpersonal trust (Respectful care)	0.52	0.76	−1	1	1797	0.62	0.75	−1	1	1281	0.56	0.76	−1	1	3078
	Informal payments	Indicator of interpersonal trust (Integrity a)	0.57	0.70	−1	1	1797	0.73	0.62	−1	1	1281	0.64	0.67	−1	1	3078
	Payment-dependent care^c^	Indicator of interpersonal trust (Integrity b)	0.41	0.78	−1	1	1165	0.54	0.74	−1	1	1281	0.48	0.76	−1	1	2446
	Comfortable conversation	Indicator of interpersonal trust (Communic./Relationship)	0.68	0.61	−1	1	1797	0.84	0.49	−1	1	1281	0.75	0.57	−1	1	3078
	Discrimination	Indicator of interpersonal trust (Fairness)	0.69	0.61	−1	1	1797	0.87	0.43	−1	1	1281	0.76	0.55	−1	1	3078
	Refusal of treatment	Indicator of interpersonal trust (Relationship/Fairness)	0.77	0.52	−1	1	1797	0.89	0.41	−1	1	1281	0.82	0.48	−1	1	3078
	Availability for injury care	Indicator of service-related trust (Treatment assurance)	0.65	0.62	−1	1	1797	0.82	0.52	−1	1	1281	0.72	0.58	−1	1	3078
	Confidence in provider skills^d^	Indicator of service-related trust (Competence)	0.37	0.62	−1	1	1797	0.54	0.55	−1	1	1281	0.45	0.60	−1	1	3078
	Tonality of health centre stories	Indicator of institutional trust (Reputation)	0.08	0.55	−1	1	1797	0.27	0.55	−1	1	1281	0.16	0.56	−1	1	3078
**Overarching trust assessments**		Trust index [-8 to +8]^e^	4.44	2.72	−5	8	1797	5.39	2.21	−4	8	1281	4.83	2.56	−5	8	3078
		Individual trust assessment [-1 to +1]^f^	0.33	0.56	−1	1	1797	0.50	0.55	−1	1	1281	0.40	0.57	−1	1	3078
		Recommendation index [0 to +4]^g^	2.29	1.47	0	4	1838	2.80	1.40	0	4	1323	2.50	1.46	0	4	3161

a Elicited only for participants who were aware of their local health centre.

b Elicited only for participants who were aware of their local health centre.

c Indicator was added after pilot survey in Champassak.

d Answer category of “no strong opinion” added as neutral response option after pilot survey in Champassak.

e Composite index of 8 trust components, using indicators of Integrity (a) in Champassak at baseline and Integrity (b) in all other cases.

f Concluding survey question after each individual dimension, asking, “Overall, would you say you rather trust or distrust the healthcare centre for your personal healthcare?” Values of [-1], [0], and [+1] correspond to responses “rather distrust,” “neutral/no opinion/don't know,” and “rather trust,” respectively.

g Based on the number of times that health centers were mentioned as recommended resorts to care for (a) antenatal care, (b) place of delivery, (c) injury treatment, and (d) COVID-19 care; including all respondents irrespective of their awareness of local health centers.

We followed a two-step mixed-method analysis strategy. In the first step, we analyzed the qualitative material through content-oriented thematic analysis to understand and contextualize the implementation setting (Haenssgen, 2020; using MAXQDA, 2020; VERBI Software, 2021). The analysis identified and coded any content relating to (a) implementation of and participation in the CONNECT Module-2 activities, and (b) outcomes of CONNECT relating to changes in trust (or the absence thereof). The diversity of respondents and settings helped to actively source negative cases to challenge evolving findings and to provide nuance for the assessment. The subsequent quantitative analysis aimed at ascertaining the engagement with and outcomes of the CONNECT activities systematically and at scale through descriptive statistical analysis (non-inferential due to the use of census data) using Stata 16 (StataCorp, 2019). The census survey design enabled a distinction within each community between villagers who remained unaware of CONNECT (“unexposed”), who were aware of the activities but did not participate (“indirectly exposed,” e.g. through word-of-mouth), and those who participated in the Module-2 workshops and/or supportive supervision activities (“directly exposed”). The two-round design made it then possible to assess indicator changes in the case study communities before and after the CONNECT activities, and among individuals who were directly, indirectly, and not at all exposed to the activities. We further analyzed the panel data set comprising 618 individuals in a difference-in-difference framework (i.e. comparing relative changes among differently exposed groups).

## 4 Results

### 4.1 Engagement with CONNECT

Among the CONNECT stakeholders, the interpretation of the Module-2 activities resonated clearly with the positions of the Initiative. Although CONNECT was often described as somewhat similar to other initiatives with elements of knowledge transfer and health education, stakeholders also and especially stressed CONNECT's relationship-building impact as well as its focus on positive potential (rather than emphasizing what is lacking). Provincial health authorities thereby indicated the success of CONNECT to instate a mindset change with greater sympathy towards the realities of target communities. A respondent from the Bokeo Provincial Health Office exemplified this point as follows:

*Before the CONNECT Initiative happened, it was still difficult for people* [in the villages] *to understand* [us] *because the knowledge that we would share with them was not able to cover all* [aspects of their lives] *and we didn’t visit them to encourage them. We did have health education, but people still didn’t come for the services. Through the CONNECT Module 2, we learned many ways to reach people: whether small groups or large groups, mobilizing people to use the health services, elderly who have not been vaccinated for COVID and who are afraid of death.* (KII, Bokeo, Provincial Health Office, Endline)

Also district- and local-level health actors interpreted the CONNECT activities in accordance with key elements of the Initiative, whereby key themes included.

• relational aspects of primary healthcare: “*Most of these* [other] *meetings are similar but CONNECT talks about building people's relationship*” (KII, Bokeo, Health Center, Endline),
• the positive approach of CONNECT: “*Making mistakes should not be said to be negative*. *We will explain to them to understand*” (KII, Champassak, District Health Office, Endline),
• respectful engagement: “*listening to people's problems, then we will share our lessons with them and ask them for more comments*” (KII, Champassak, District Health Office, Endline),
• community ownership and engagement: “*Before* [CONNECT], *the community didn't have the ownership to cooperate*. […] *Therefore, we want to have a CONNECT team to encourage the village deputy*” (KII, Bokeo, District Health Office, Endline), and
• collaboration and equity: “*As I understood it, the CONNECT Initiative is about everyone helping and working together, the village and the health center; health education and equal access to health services. Previously our health center also always did that, but not the full extent [that we have realized] since CONNECT*” (KII, Champassak, Health Center, Endline).

At the community level, interpretations of the CONNECT village-based activities were more diverse, although key aspects of the Initiative remained discernible. For example, several village chiefs interpreted CONNECT as a regular health education campaign as “*They're similar in giving advice in healthcare*” (KII, Khammouane, Village Chief, Endline) and that “*after this work, we came to spread awareness to the public and advise the right to receive social security for various treatments and advice on how risky it is to give birth at home to pregnant women*” (KII, Bokeo, Village Chief, Endline). However, other respondents also encouragingly emphasized CONNECT's focus on “ownership in health care” (KII, Bokeo, Village Chief, Endline), and a village health volunteer, too, stressed the relational aspects of the Initiative by arguing that, “*everyone has to be harmonized as one and as the orange tree. Like, if they want to have a good health center, but we also have to help each other*” (SSI, Bokeo, Male villager, Endline). A workshop participant from the Lao Front elaborated eloquently on the role of this mechanism in achieving health objectives:

*The CONNECT workshop trained us about the harmony between the village authorities and the villagers*. […] *We are like the mango tree* [i.e. respondent is alluding to the action plan tree]: *we need to collaborate and be harmonious to accomplish our work. If we are not harmonious, then we are like a mango tree without fertilizer, which leaves the mango tree unable to produce fruits*. (SSI, Champassak, Male villager, Endline)

**Fig. 2:**
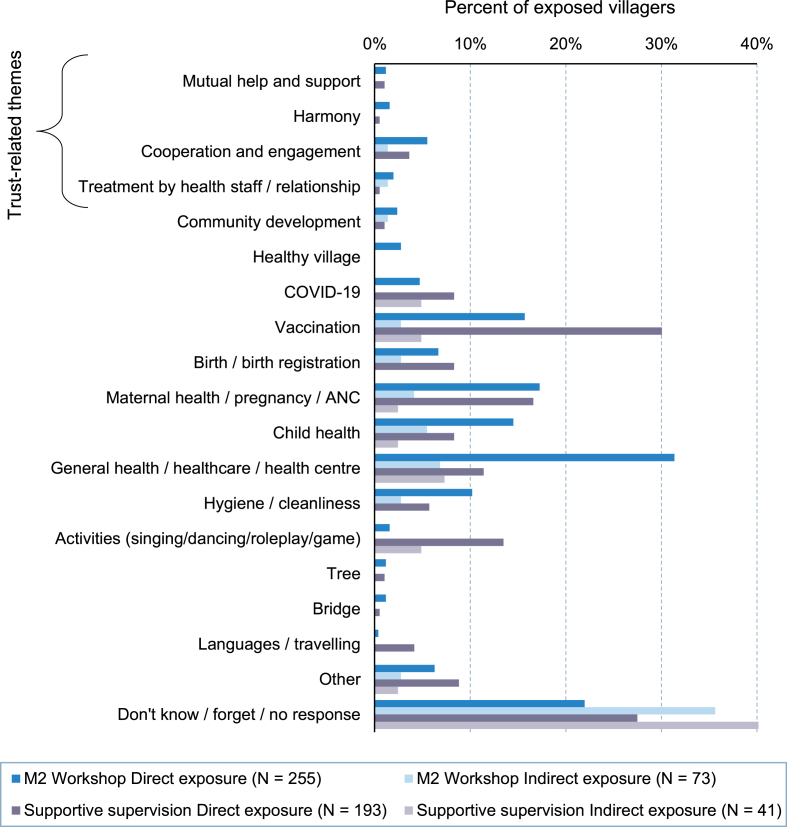
Interpretations of CONNECT M2 workshops and supportive supervision by exposure (direct participation vs. being aware without participation). *Notes.* Percentage share based on respective subgroup. Multiple responses possible; coded based on free-text responses.

In contrast to community-level authorities and broader healthcare stakeholders, very few interpretations of the workshop and supportive supervision among the community members departed from standard health education campaigns (see Fig. 2 for an overview of topic interpretations). None of the community members – neither in interviews nor in the survey responses – explicitly mentioned the role of trust within the CONNECT activities. However, indirect references to trust included mutual help, community engagement in and ownership of healthcare, relationships to healthcare staff, and “*harmony*” (SSI, Champassak, Female villager, Endline), which materialized among 10.5% of the workshop participants and 5.4% of the supportive supervision participants. Interview respondents from all three provinces with CONNECT activities thereby perceived the workshops as “*not really different*” (KII, Champassak, Male villager, Endline) from standard health promotion campaigns and described them as “*about going to the health center, going to the hospital*” (KII, Bokeo, Female villager, Endline) and generally “about health care. It may not really be different” (KII, Khammouane, Female villager, Endline).

As shown in the breakdown in Fig. 2, the main topics that villagers thus recalled from the workshops were interpreted as common health education issues of COVID vaccination, delivery at the health center, seeking healthcare from the local health center, and community and personal hygiene. Yet, villagers were in many instances unable to articulate the content or objectives of the activities at all and would simply explain that they did not understand the activities and “*did not learn anything. Just sit and watch*” (SSI, Bokeo, Female villager, Endline). Villagers who were only exposed to the activities through word-of-mouth or by seeing their outputs (e.g. the action plan tree) were yet more likely to simply state that they did not know what the CONNECT activities were about (see light-shaded bars in Fig. 2).

### 4.2 Qualitative perspectives on the trust impact of the CONNECT workshops

The CONNECT Initiative was part of a complex healthcare context that was noticeably – and increasingly – resource constrained (aggravated by a strong exchange rate decline and high inflation). Stakeholders were conscious of these constraints, as in the case of a respondent from a District Health Office in Champassak Province:

*One of the areas that is not doing well is the service area. Our community hospital is still a small hospital. When there are many people coming to use the service, we may not be able to provide treatment because our staff is limited and the supply is not full*. (KII, Champassak, District Health Office, Endline)

Health center staff recognized this limitation as well – across all provinces (e.g. “*The biggest problems we face presently are about the lack of medicine*,” KII, Xaisomboun, Health Center, Baseline) but especially so in Bokeo Province: When asked about the priority areas for health service improvements, health center staff argued repeatedly about their limited facilities which “*might need the support for new equipment* […] *and computer support* […] *and budget to expand the health center*” (KII, Bokeo, Health Center, Baseline) as well as about the challenges in reaching their catchment population:

*The difficult* side [of our service provision is] *for patients in severe cases or for those with low income or who don’t have vehicles*. *Pregnant women* […] *sometimes couldn’t come* [to their appointments] *and that would make them miss the opportunities and force them to give birth at their houses*. *For general patients or kids below five years*, [… their parents might at times have to] *spend around five hours to reach the health center*. (KII, Bokeo, Health Center, Baseline)

Despite challenges in physical service provision, our foundational research (reported in Haenssgen et al., 2024) had established that the residents of the 14 study communities widely understood the concept of trust in primary healthcare, readily explained different dimensions such as service quality or assurance of care, and responded to their trust assessments through their treatment-seeking behavior. Healthcare workers corroborated this view as they argued towards the need to increase trust between them and the local population: “To reduce fear of discrimination, *[there is a need]* to talk or advice the villagers and patients that they shouldn't be afraid for the gap in services at the health center” (KII, Champassak, Health Center, Baseline).

Against the backdrop of these baseline conditions, the enhancement of trust through the village-based CONNECT activities was a success story: Although complex health system and infrastructural challenges continued to constrain the impact of the trust-building activities, stakeholders and villagers alike reported widespread improvements in trust between the health system and local communities. Voices from higher-level healthcare stakeholders at endline would stress the strengthened relationships towards local communities, enabled by more respectful communication, empathy, and a growing mutual understanding. For example, a respondent from a District Health Office in Xaisomboun Province who had already been exposed to the CONNECT Initiative at baseline explained that, “*now, after CONNECT*, *we let* [the community members] *speak and give examples of the problems and have them find the solutions*” (KII, Xaisomboun, District Health Office, Baseline) while a District Health Office respondent in Bokeo Province stressed the direct trust impact of CONNECT: “*As I have observed, I could say that it* [trust after CONNECT] does change a lot*. It is better if we have CONNECT help people to understand more and it'll make them come use the services more*” (KII, Bokeo, District Health Office, Endline).

Health center staff echoed this positive sentiment. Khammouane healthcare staff stated clearly and powerfully that, “*I can see that people's trust has changed a lot, in a better way*” (KII, Khammouane, Health Center, Baseline), and the Head of a Health Center in Bokeo Province explained at length the improved mutual understanding between healthcare workers and communities that CONNECT has enabled:

*Before* [CONNECT]*, most people might feel disappointed about the relationship* [to the health center] *when we couldn’t support them as they expected us to do.* […] *For example, when people don’t understand* [us]*, they might scold us or complain about the services. But we have to be patient to build the relationship as we have been trained* [by CONNECT]*. We have to make people understand about the services, and if we really couldn’t make people understand, if we make mistakes or couldn’t fix it, then we’ll also discuss with the* [health center] *team.* (KII, Bokeo, Health Center, Endline)

Analogous to these positive impressions on the side of the health system, community authorities and members voiced similarly positive impressions about changes in trust. A village chief in Khammouane Province reported that, “*It can be seen that there is a difference. People talk more easily. In the past, people were still afraid of doctors*” (KII, Khammouane, Village Chief, Endline). Villagers in Champassak Province attributed the CONNECT workshop to “*changes in the health center staff's behavior; they take care of us better than before*” (SSI, Champassak, Feale villager, Endline) and that “*it has changed a lot and they're more friendly*” (KII, Champassak, Female villager, Endline). A pregnant woman visiting a health center in Champassak for ante-natal care after the workshop thereby reported her positive experience in which the health center staff “*talk nicely. They ask me if I feel hurt somewhere when they touch my stomach*” and therefore clearly stated that she trusts them (“*yes*, *I do*,” SSI, Champassak, Female villager, Endline). In short, the qualitative reports of changes in trust were overwhelmingly positive.

Despite the visible improvements, changes of trust were also subject to inertia and contextual constraints. Even at endline, the evaluation team was privy to stories of neglect, poor treatment experiences, and discrimination at local health centers that left villagers disillusioned. While relationships clearly mattered to the respondents, they also explicitly highlighted that their trust depended on the ability of the health centers to deliver quality care. Especially the availability of medicine – which had become an ever more pressing health system challenge in the evolving economic climate – had been a key obstacle for villagers to trust their local health centers: “*I do not trust the health center because I am afraid that they do not have enough medicine*” (SSI, Champassak, Male villager, Endline). A villager in Bokeo Province who attended the CONNECT workshop would still explain at endline that she would only give the health center a trust score of 1 on a scale from 1 to 10 because “*there were no medicines at the health center and I had to go buy it outside at their* [staff members’] *house*” (SSI, Bokeo, Female villager, Endline).

### 4.3 Quantitative analysis on CONNECT short-term impact on trust

**Fig. 3:**
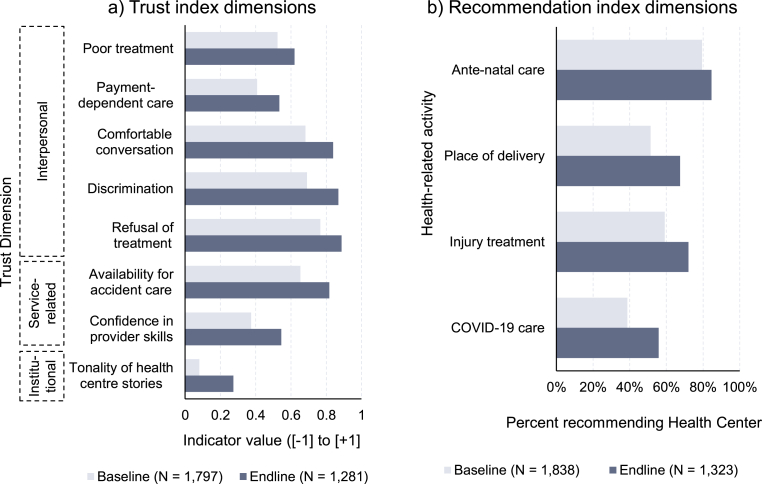
Change in (a) trust indicators and (b) health center as recommended resort to care between baseline and endline surveys. *Notes.* Baseline and endline data. Payment-dependent care excludes Champassak Province (indicator was added after pilot survey). Note that differences in cross-sectional sample composition may affect representation across baseline and endline surveys.

To explore whether the qualitative impressions were reflected at a broader scale, the statistical evaluation data from the community surveys allowed more detailed insights into the effects of CONNECT on the various dimensions of trust as well as on people's inclination to recommend health centers as a resort to care. That these impressions were not merely circumstantial but indeed systematic was evidenced in the survey data: whereas 36.7% of the respondents at baseline indicated that they would “rather trust” their local health center, a considerably higher portion of 51.3% would do so at endline. The composite indices showed similar positive trends across the survey rounds as the average reported trust index increased by 0.95 points from 4.44 to 5.39 (on a scale from [–8 to +8]) and the recommendation index by 0.51 points from 2.29 to 2.80 (on a scale from [0 to +4]). Disaggregated data across the index dimensions (Fig. 3) showed a similarly encouraging picture: Panel a in Fig. 3 demonstrates that, despite the somewhat hesitant qualitative responses, *all* dimensions of trust experienced an improvement between the baseline and endline data collection. While especially the interpersonal dimensions were expected to improve as a result of the activities (average change across the five indicators: +0.14 indicator points, +22.7% relative change), yet stronger absolute and relative improvements were in fact registered in the areas of service-related (+0.17 indicator points; +35.3%) and institutional trust (+0.19 indicator points; +241.2%). Panel b in Fig. 3 shows that, following the CONNECT village-based activities, health centers were mentioned more commonly as recommended resorts to care for (a) antenatal care, (b) place of delivery, (c) injury treatment, and (d) COVID-19 care.

**Fig. 4:**
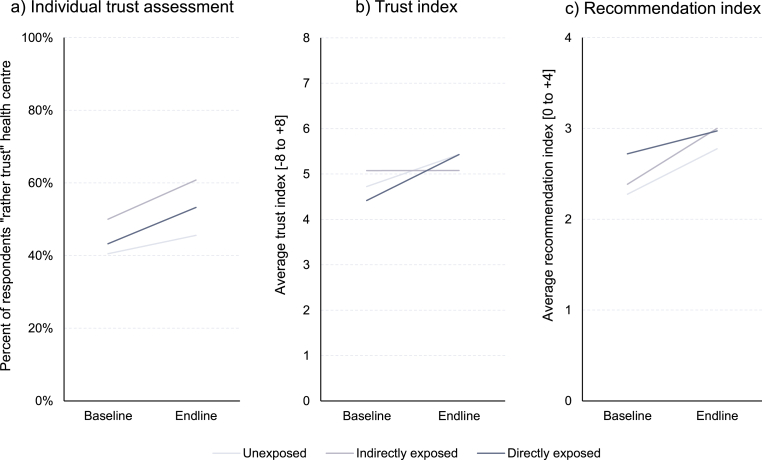
Change in trust measures across baseline and endline surveys by exposure to CONNECT activities. *Notes.* Panel data. *N* = 1217 for Panels a and b (limited to respondents familiar with their local health centre) and *N* = 1236 for Panel c.

The final analysis step involved the assessment of indicator changes in the matched panel data set through a difference-in-difference analysis. Fig. 4 shows (a) the overarching individual assessment whether respondents would “rather trust” their health center, (b) the 8-item trust index, and (c) the 4-item recommendation index (see Supplemental Material Table A2 for the underlying statistics and detailed indicator breakdown). All three measures improved among both the exposed (dark-shaded lines) and unexposed groups (light-shaded lines). For example, the trust index at endline was on average 1.02 index points higher than at baseline for villagers exposed to the CONNECT activities. The same group also recorded a 10 percentage-point higher response of “rather trust[ing]” in their health center, and a 0.25-point higher recommendation index. The relative improvement of the trust index and overall trust judgement among the exposed group outpaced those of the unexposed groups (+0.31 index points and +4.9 percentage points, respectively), whereas unexposed groups a exhibited relatively stronger increase in the recommendation index (−0.25 index points). In contrast, indirect exposure through word-of-mouth was associated with comparatively weaker trust index developments but relatively more favorable developments of the recommendation index and overall trust judgement.

Among the component dimensions of the trust index (each on a scale from [–1 to +1], shown in Supplemental Material Table A2), exposed individuals registered improved indicators across all dimensions except respectful treatment (−0.03 indicator points), whereas the general development of the responses among all unexposed individuals was consistently positive across all dimensions. Interestingly, this meant that, relatively to the general trend among unexposed individuals, participants of the CONNECT activities were relatively *less* likely (−0.16 indicator points) to report on respectful care (indicated through poor treatment) from their local health center at endline. However, all other indicators among the CONNECT participants developed either in line with the trend or yet more positively – especially in terms of discrimination (indicator improved by +0.12 points), refusal of treatment (improvement by +0.07), and confidence in healthcare providers’ skills (+0.07 points).

Separating the difference-in-difference analysis of the trust index by province, gender, and ethnicity offered further insights into the spatial, temporal, and social distribution of the outcomes: While the index increased in all provinces among exposed and unexposed groups alike, the relative change among the exposed group was negative in Champassak Province (−0.41 indicator points), became positive in Bokeo and Khammouane Provinces (+0.35 and + 0.25 indicator points), and yet more pronounced in Xaisomboun Province (+1.07 indicator points) – reflecting thus spatial heterogeneity that followed approximately the implementation timeline of the CONNECT Initiative. The trust index among men increased at a relatively faster pace than women (+0.54 vs. +0.08 relative change compared to unexposed men and women), thereby starting both from a lower (4.27 vs. 4.55) and reaching higher level (5.58 vs. 5.29) at endline in absolute terms. Similarly, ethnic minority groups registered a faster gain in the trust index compared to the Lowland Lao majority population (+0.31 vs. +0.19), again starting at a lower (4.30 vs. 4.66) and reaching a higher absolute index level (5.49 vs. 5.31). In all these comparisons, indirectly exposed villagers exhibited highly variable absolute and relative index developments.

## 5 Discussion

Speaking to the strong potential yet lacking evidence of community engagement approaches to foster trust between communities and healthcare providers, our study provided rare empirical evidence from a mixed-method process evaluation to assess the implementation and short-term trust impact of the CONNECT initiative in Lao PDR. Examining the experiences of 14 case study communities, we provided systematic evidence that the introduction of the CONNECT Initiative contributed to improvements in community members’ trust in their primary healthcare services. Institutional and service-related forms of trust registered the largest positive changes at endline while, relative to exposure, CONNECT participants registered the largest positive changes in the dimensions of discrimination and treatment refusal (interpersonal trust) and perceived confidence in provider skills (service-related trust). Alongside plausible systemic effects and contextual developments that yielded more trusting responses in the endline data collection, the difference-in-difference analysis enabled a direct attribution of disproportionate increases in trust to the CONNECT activities.

Compared to the existing literature, our evaluation of the CONNECT Initiative provided strong and systematic support for the typically indicative or anecdotal assessments of trust impacts in community engagement activities (Loewenson et al., 2021; WHO WPRO, 2023). It further suggested that a community engagement approach can be superior to conventional (i.e. selective and/or top-down) trust-building measures in healthcare, which had been reported with limited success from high-income contexts (Brennan et al., 2013; Rolfe et al., 2014). However, trust does not follow automatically from community engagement, as was also cautioned previously in the literature (De Weger et al., 2018; Kilpatrick, 2009; Petts, 2008): The experience of CONNECT demonstrated the critical importance of (and extensive work involved in) cultivating support and understanding from institutional stakeholders, and the Initiative itself underwent a visible learning process with the gradual strengthening of its trust outcomes over time. At the same time, the spread of awareness through word-of-mouth could create unpredictable trust outcomes as villagers gained only a rudimentary understanding of the activity's content and intentions. The range of personal, historical, and other external drivers of trust (Larson et al., 2018; Murray and McCrone, 2015) further suggested heterogeneous distributional consequences of trust-building activities, which corresponded with our observations that male and ethnic minority respondents registered the largest absolute and relative (difference-in-difference) changes. The role of such factors was evidenced in related research in Lao PDR as well, where trust in vaccines created particularly strong changes in COVID-19 vaccine uptake among the patriarchal and male-dominated Hmong ethnic minority in Lao PDR (Elliott et al., 2024, Phrasisombath et al., 2024).

The nature of our study also requires further discussion. Among others, the in-depth study of 14 communities contains a diverse sample of cases across rural settings of Lao PDR, but it cannot claim representativeness for the country as a whole and calls for further community engagement evaluation research in other diverse geographies. In contexts such as rural Lao PDR where local populations were not previously exposed to relational community engagement approaches, it is possible that the trust building activities were understood correctly but interpreted within existing frames of reference. This could explain the common references to health education (rather than “community engagement”) and harmony (rather than “trust”) in the presence of potential systemic trust outcomes of CONNECT. Understanding these systemic effects (which the qualitative data corroborated) would benefit from further operational research involving unexposed contexts as “control groups.” The continued circulation of negative stories further suggested a certain inertia in enhancing community trust (thereby also underlining that trust is dynamic and changes over time and relative to the landscape of healthcare providers), which would require not only continuing relationship- and trust-building activities in the communities but also a longer-term assessment period. Quasi-experimental research designs using multi-round nationally representative household survey data such as the Lao Social Indicator Survey could help respond to these empirical research needs (Lao Statistics Bureau, 2024).

## 6 Conclusion

While the importance of trust in healthcare is recognized in global health policy, practice, and scholarship, measures to actively build patient trust in healthcare have hitherto demonstrated only mixed results. Community engagement as a promising approach to leverage and build trust has received particularly strong attention since the COVID-19 pandemic but evaluation knowledge on its trust impact remains scarce. Experiences from low- and middle-income countries can thereby offer not only global-health-relevant insights into new health policy approaches, but also help develop our understanding of the nuanced and contextualised expressions of patient trust in healthcare.

Drawing on the case of the CONNECT Initiative in Lao PDR with a specific focus on patient trust in primary healthcare, our study offered a rare and detailed insight into the successful development of trust in rural settings of a lower-middle-income country. Mixed-method evidence demonstrated a direct as well as systemic improvement of trust – in general and across its component dimensions – that was surprisingly strong and consistent, whereby sub-populations (men, ethnic minority groups) appeared to benefit more than others. It can therefore be concluded that the CONNECT Initiative had a considerable short-term effect on community members’ trust in their local health centers, which arose from participatory workshops to build trust and the subsequent reinforcement of the workshop outcomes through supportive supervision activities. The outcomes were enabled by strong stakeholder mobilization and became gradually stronger with institutional learning.

By understanding the ability of a community engagement initiative to build trust and improve the relationship between the general population and the health system, this research helps explore untapped potential in improving the health of rural communities in low- and middle-income countries. However, the setting of Lao PDR as a resource-constrained context that is currently experiencing high inflation and a strong exchange rate decline also requires words of caution: The contribution of trust to boost health service uptake may remain limited unless essential medicines and equipment are in place at the primary care level, and the prolonged unavailability of essential medicines may even exacerbate villager's distrust to the health system. The effective use of flexible funding (such as a World Bank loan intended to improve healthcare quality) could thereby directly replenish essential commodities and thereby enable trust to have a positive effect on health service uptake.

By highlighting relational aspects of health and health policy, community engagement approaches such as CONNECT can therefore help realize synergies with physical and organizational health policy approaches. In the medium term, it may be useful to integrate the CONNECT approach firmly into national health system strategies and expand its integrative sentiment for cross-sectorial strategies with view towards “health [and trust] in all policies.” Given the multiple dimensions of trust and their varied influencing factors, processes to operationalize trust in policy should thereby build on community consultations and other participatory community engagement mechanisms as integral components to ensure that the most pressing problem areas and experiences of trust can be addressed.

## Ethical approval

We received ethical approval from the Lao National Ethics Committee for Health Research (NECHR; ref. 069/NECHR). Prior informed consent was obtained from all study participants. The data collection in was integrated into government structures: public health authorities facilitated village access and data collection; operational insights from each survey mission were fed back to village and health authorities; and survey findings were shared with provincial and central government authorities (including MoH and MoHA).

## Funding sources

The research was funded by the 10.13039/100004423World Health Organization Regional Office # 202634961.

## Consent for publication

N/A.

## CRediT authorship contribution statement

**Marco J. Haenssgen:** Writing – review & editing, Writing – original draft, Visualization, Validation, Supervision, Project administration, Methodology, Investigation, Formal analysis, Data curation, Conceptualization. **Elizabeth M. Elliott:** Writing – review & editing, Project administration, Methodology, Conceptualization. **Sysavanh Phommachanh:** Writing – review & editing, Methodology. **Ounkham Souksavanh:** Writing – review & editing, Supervision, Project administration, Methodology. **Hironori Okabayashi:** Writing – review & editing, Supervision, Resources, Project administration. **Shogo Kubota:** Writing – review & editing, Supervision, Resources, Project administration, Funding acquisition, Conceptualization.

## Declaration of competing interest

We declare that no conflict of interest – financial or otherwise – exists. Please note that the authors alone are responsible for the views expressed in this article and they do not necessarily represent the views, decisions or policies of the institutions with which they are affiliated.

## Data Availability

The data that has been used is confidential.

## References

[bib1] Adebisi Y.A., Rabe A., Lucero-Prisno III D.E. (2021). Risk communication and community engagement strategies for COVID-19 in 13 African countries. Health Promot. Perspect..

[bib2] Adhikari B., Yeong Cheah P., von Seidlein L. (2022). Trust is the common denominator for COVID-19 vaccine acceptance: a literature review. Vaccine X.

[bib3] Birkhäuer J., Gaab J., Kossowsky J. (2017). Trust in the health care professional and health outcome: a meta-analysis. PLoS One.

[bib4] Bochaton A. (2015). Cross-border mobility and social networks: laotians seeking medical treatment along the Thai border. Soc. Sci. Med..

[bib5] Brennan N., Barnes R., Calnan M. (2013). Trust in the health-care provider-patient relationship: a systematic mapping review of the evidence base. Int. J. Qual. Health Care.

[bib6] Buchner D. (2011).

[bib7] Coulombe H., Epprecht M., Pimhidzai O. (2016). Vientiane: Ministry of Planning and Investment.

[bib8] De Weger E., Van Vooren N., Luijkx K.G. (2018). Achieving successful community engagement: a rapid realist review. BMC Health Serv. Res..

[bib9] Dobility Inc. (2017).

[bib10] Elliott E. (2021).

[bib11] Elliott E.M., Kubota S., Robinson D.R. (2024). Gradients of trust in vaccines: embodied inequities, religion, and relational care in Laos. Asian Med..

[bib12] Ezumah N., Manzano A., Ezenwaka U. (2022). Role of trust in sustaining provision and uptake of maternal and child healthcare: evidence from a national programme in Nigeria. Soc. Sci. Med..

[bib13] Gilmore B., Ndejjo R., Tchetchia A. (2020). Community engagement for COVID-19 prevention and control: a rapid evidence synthesis. BMJ Glob. Health.

[bib14] Gopichandran V., Chetlapalli S.K. (2013). Dimensions and determinants of trust in health care in resource poor settings – a qualitative exploration. PLoS One.

[bib15] Gopichandran V., Wouters E., Chetlapalli S.K. (2015). Development and validation of a socioculturally competent trust in physician scale for a developing country setting. BMJ Open.

[bib16] Gryseels C., Kuijpers L.M.F., Jacobs J. (2019). When ‘substandard’ is the standard, who decides what is appropriate? Exploring healthcare provision in Cambodia. Crit. Publ. Health.

[bib17] Haenssgen M.J. (2015). Satellite-aided survey sampling and implementation in low- and middle-income contexts: a low-cost/low-tech alternative. Emerg. Themes Epidemiol..

[bib18] Haenssgen M.J. (2019). New impulses from international development for more comprehensive and balanced public engagement evaluation. Glob. Health Action.

[bib19] Haenssgen M.J. (2020).

[bib20] Haenssgen M.J., Charoenboon N., Zanello G. (2018). Antibiotics and activity spaces: protocol of an exploratory study of behaviour, marginalisation, and knowledge diffusion. BMJ Glob. Health.

[bib21] Haenssgen M.J., Elliott E.M., Phommachanh S. (2024). Trust in healthcare: methodological and conceptual insights from mixed-method research in Lao People's Democratic Republic. BMJ Glob. Health.

[bib22] Halbert C.H., Weathers B., Delmoor E. (2009). Racial differences in medical mistrust among men diagnosed with prostate cancer. Cancer.

[bib23] Kilpatrick S. (2009). Multi-level rural community engagement in health. Aust. J. Rural Health.

[bib24] Kim E., Park Y.L., Lo Y.-R. (2024). Sustaining essential health services in Lao PDR in the context of donor transition and COVID-19. Health Pol. Plann..

[bib25] Lao Statistics Bureau (2024).

[bib26] Larson H.J., Clarke R.M., Jarrett C. (2018). Measuring trust in vaccination: a systematic review. Hum. Vaccines Immunother..

[bib27] Lee T.H., McGlynn E.A., Safran D.G. (2019). A framework for increasing trust between patients and the organizations that care for them. JAMA.

[bib28] LoCurto J., Berg G.M. (2016). Trust in healthcare settings: scale development, methods, and preliminary determinants. SAGE Open Medicine.

[bib29] Loewenson R., Colvin C.J., Szabzon F. (2021). Beyond command and control: a rapid review of meaningful community-engaged responses to COVID-19. Global Publ. Health.

[bib30] Mechanic D. (1996). Changing medical organization and the erosion of trust. Milbank Q..

[bib31] Milton B., Attree P., French B. (2011). The impact of community engagement on health and social outcomes: a systematic review. Community Dev. J..

[bib32] Murray B., McCrone S. (2015). An integrative review of promoting trust in the patient–primary care provider relationship. J. Adv. Nurs..

[bib33] Odugleh-Kolev A., Parrish-Sprowl J. (2018). Universal health coverage and community engagement. Bull. World Health Organ..

[bib34] OECD Development Assistance Committee (2019).

[bib35] Ozawa S., Sripad P. (2013). How do you measure trust in the health system? A systematic review of the literature. Soc. Sci. Med..

[bib36] Ozawa S., Walker D.G. (2011). Comparison of trust in public vs private health care providers in rural Cambodia. Health Pol. Plann..

[bib37] Petts J. (2008). Public engagement to build trust: false hopes?. J. Risk Res..

[bib38] Phommachanh S., Essink D.R., Jansen M. (2019). Improvement of quality of antenatal care (ANC) service provision at the public health facilities in Lao PDR: perspective and experiences of supply and demand sides. BMC Pregnancy Childbirth.

[bib39] Phommachanh S., Essink D.R., Wright E.P. (2019). Do health care providers give sufficient information and good counseling during ante-natal care in Lao PDR?: an observational study. BMC Health Serv. Res..

[bib40] Phrasisombath K., Kubota S., Elliott E.M. (2024). Reaching the unreached through building trust: a mixed-method study on COVID-19 vaccination in rural Lao PDR. BMJ Glob. Health.

[bib41] Pilgrim D., Vassilev I. (2010).

[bib42] Questa K., Das M., King R. (2020). Community engagement interventions for communicable disease control in low- and lower- middle-income countries: evidence from a review of systematic reviews. Int. J. Equity Health.

[bib43] Rasiah S., Jaafar S., Yusof S. (2020). A study of the nature and level of trust between patients and healthcare providers, its dimensions and determinants: a scoping review protocol. BMJ Open.

[bib44] Rodrigues C.F. (2021). Communicative trust in therapeutic encounters: users' experiences in public healthcare facilities and community pharmacies in Maputo, Mozambique. Soc. Sci. Med..

[bib45] Rolfe A., Cash-Gibson L., Car J. (2014). Interventions for improving patients' trust in doctors and groups of doctors. Cochrane Database Syst. Rev..

[bib46] Sousa‐Duarte F., Brown P., Mendes A.M. (2020). Healthcare professionals' trust in patients: a review of the empirical and theoretical literatures. Sociology Compass.

[bib47] StataCorp (2019).

[bib48] Sychareun V., Hansana V., Somphet V. (2012). Reasons rural Laotians choose home deliveries over delivery at health facilities: a qualitative study. BMC Pregnancy Childbirth.

[bib49] Sychareun V., Nouanthong P., Thongmyxay S. (2022).

[bib50] Taylor L.A., Nong P., Platt J. (2023). Fifty years of trust research in health care: a synthetic review. Milbank Q..

[bib51] VERBI Software (2021).

[bib52] Ward P.R. (2017). Improving access to, use of, and outcomes from public health programs: the importance of building and maintaining trust with patients/clients. Front. Public Health.

[bib53] WHO (2017).

[bib54] WHO (2020).

[bib55] WHO WPRO (2023).

[bib56] Wikimedia Commons (2023). Wikimedia commons. https://commons.wikimedia.org.

[bib57] Wilcox P., Daljit S., Hoang Thi H. (2022). Southeast Asian Affairs 2022.

[bib58] World Bank (2023). World databank.

