# Supplementary material for: Community engagement for stakeholder and community trust in healthcare: Short-term evaluation findings from a nationwide initiative in Lao PDR
Source: Soc Sci Med. 2024 Aug;354:117079. doi: 10.1016/j.socscimed.2024.117079 (PMC11413521; doi:10.1016/j.socscimed.2024.117079)
Supplement: Multimedia component 1 [file mmc1.pdf]

Table A1. Case study site characteristics.

Province	Village	Registered population[a]	Village population[b]			Village attributes (baseline sample averages)								
			Residents	of which: Adults[c]	Baseline survey sample[d]	Household size[e]	Female[f]	Age	Years of education	Literate[g]	Wealth index[e,h]	Lao Loum	Currently / recently pregnant[i]	Disabled[j]
Champassak	**1:** relatively remote	298	224	153	108	5.3	52.8%	40.6	5.0	82.2%	47.7%	90.7%	10.2%	5.6%
	**2:** near military hospital	1152	708	521	318	5.5	55.3%	43.9	7.0	83.6%	60.8%	94.7%	6.9%	1.3%
	**3:** centrally located	1168	650	472	232	5.2	62.5%	43.2	6.4	80.6%	59.7%	77.2%	8.2%	0.4%
Bokeo	**1:** moderately remote Khmu village	600	545	320	108	7.0	51.9%	37.4	3.9	58.3%	29.1%	1.9%	10.2%	2.8%
	**2:** remote + poor Khmu village	316	259	155	65	5.7	46.2%	40.3	4.6	66.2%	22.3%	3.1%	9.2%	7.7%
	**3:** centrally located Lamed village	561	449	333	138	5.0	47.8%	45.5	4.0	73.9%	39.8%	15.2%	5.8%	1.4%
	**4:** moderately remote Lamed village	504	462	299	129	5.5	41.1%	36.5	5.0	80.6%	31.4%	0.0%	8.5%	0.0%
Khammouane	**1:** small Phu Tai & Lao Therng village	173	140	76	43	5.4	51.2%	35.3	1.3	34.9%	21.6%	7.0%	9.3%	2.3%
	**2:** remote Phu Tai village	243	276	168	70	6.1	42.9%	37.0	2.7	49.3%	34.4%	17.1%	5.7%	2.9%
	**3:** accessible & affluent Phu Tai village	427	326	211	109	4.7	55.0%	40.3	4.7	67.0%	56.3%	45.0%	9.2%	0.0%
Xaisomboun	**1:** moderately remote Hmong village	1064	691	397	161	7.2	55.9%	36.4	5.5	55.9%	47.8%	1.9%	11.2%	0.0%
	**2:** central Hmong village	1527	418	252	122	7.5	54.1%	37.0	5.8	61.5%	44.6%	0.0%	9.0%	1.6%
	**3:** central Hmong & Khmu village	1810	991	632	169	6.7	43.2%	41.4	4.8	75.1%	49.2%	12.4%	3.6%	2.4%
	**4:** mod. remote Hmong & Khmu village	1173	375	238	66	5.8	36.4%	37.3	4.2	74.2%	54.6%	7.6%	7.6%	0.0%
	**Total / average**	11016	6514	4227	1838	5.9	51.6%	40.4	5.2	71.7%	46.0%	37.9%	7.9%	1.6%

Note: Baseline survey data as of December 2023.

[a.] Based on administrative data.

[b.] Based on village census data.

[c.] Aged ≥16 years, including household members temporarily absent due to labor migration, household members unable to partake e.g. due to illness, and household members who declined participation.

[d.] Aged ≥16 years.

[e.] Statistics reported on household level (not individual level).

[f.] No respondent disclosed a non-binary gender identity.

[g.] Self-reported ability to read Lao.

[h.] Simple aggregate index (from [0] to [1]) of 8 common household assets (7 types of items incl. cars and rice cooker plus ratio of [>1] for number of rooms per household member)

[i.] Recently pregnant includes women who delivered within the last 12 months prior to the survey interview.

[j.] Self-identified; including here only physical disabilities (no other disabilities were disclosed by participants in the current sample).

[k.] 1st round survey had to be stopped early due to dangerous weather conditions.

Table A2. Overview of key trust indicators following CONNECT activities from matched panel dataset.

	Indicator	Exposure						First Difference			Difference-in-difference		N
		Unexposed		Indirect		Direct		Unex-posed	Indirect	Direct	Direct vs. unexposed	Indirect vs. unexposed	
		Baseline	Endline	Baseline	Endline	Baseline	Endline						
	**Percent of respondents "rather trust" health centre**	40.5%	45.6%	50.0%	60.8%	43.3%	53.2%	+5.1 p.p.	+10.8 p.p.	+10.0 p.p.	+4.9 p.p.	+5.7 p.p.	1217
	**Average trust index [-8 to +8]**	4.72	5.43	5.08	5.08	4.41	5.43	+0.71	+0.00	+1.02	+0.31	–0.71	1217
	**Average recommendation index [0 to +4]**	2.28	2.78	2.38	3.00	2.72	2.97	+0.50	+0.62	+0.25	–0.25	+0.12	1236
**Trust dimensions [-1 to +1]**	**Poor treatment**	0.54	0.67	0.56	0.67	0.62	0.59	+0.13	+0.11	–0.03	–0.16	–0.03	1217
	**Payment-dependent care**	0.47	0.54	0.18	0.33	0.39	0.53	+0.07	+0.15	+0.14	+0.06	+0.08	1001
	**Comfortable conversation**	0.71	0.84	0.73	0.84	0.68	0.84	+0.13	+0.11	+0.16	+0.03	–0.02	1217
	**Discrimination**	0.75	0.90	0.77	0.78	0.64	0.92	+0.15	+0.02	+0.27	+0.12	–0.14	1217
	**Refusal of treatment**	0.79	0.90	0.81	0.82	0.72	0.90	+0.11	+0.02	+0.18	+0.07	–0.10	1217
	**Availability for accident care**	0.67	0.81	0.87	0.80	0.68	0.82	+0.14	–0.06	+0.14	+0.00	–0.20	1217
	**Confidence in provider skills**	0.38	0.52	0.54	0.59	0.36	0.57	+0.13	+0.05	+0.21	+0.07	–0.08	1217
	**Tonality of health centre stories**	0.10	0.25	0.17	0.24	0.07	0.26	+0.16	+0.06	+0.20	+0.04	–0.09	1217
**Province (chro-nological order)**	**Champasak: Average trust index [-8 to +8]**	4.66	5.24	5.74	5.83	4.81	4.98	+0.58	+0.10	+0.18	–0.41	–0.49	429
	**Bokeo: Average trust index [-8 to +8]**	4.64	5.50	4.82	4.73	4.23	5.44	+0.86	–0.09	+1.21	+0.35	–0.95	388
	**Khammouane: Average trust index [-8 to +8]**	4.83	6.10	4.50	6.50	4.30	5.82	+1.27	+2.00	+1.52	+0.25	+0.73	174
	**Xaisomboun: Average trust index [-8 to +8]**	4.90	5.21	4.44	4.11	4.34	5.71	+0.30	–0.33	+1.37	+1.07	–0.64	226
**Gender**	**Male: Average trust index [-8 to +8]**	4.78	5.54	5.45	5.05	4.27	5.58	+0.77	–0.40	+1.31	+0.54	–1.17	569
	**Female: Average trust index [-8 to +8]**	4.68	5.34	4.84	5.10	4.55	5.29	+0.66	+0.25	+0.74	+0.08	–0.40	648
**Ethnic Group**	**Minority ethnic: Average trust index [-8 to +8]**	4.66	5.54	4.80	4.83	4.30	5.49	+0.88	+0.03	+1.19	+0.31	–0.85	765
	**Majority ethnic: Average trust index [-8 to +8]**	4.81	5.27	5.65	5.63	4.66	5.31	+0.46	–0.02	+0.65	+0.19	–0.48	452

Interview data [*Record observation*]		
i. District Number ໝາຍເລກເຂດ		[***code entered automatically***] [ຂໍ້ມູນຖືກບັນທຶກໂດຍອັດຕະໂນມັດ]
ii. Village Number ໝາຍເລກບ້ານ		[***code entered automatically***] [ຂໍ້ມູນຖືກບັນທຶກໂດຍອັດຕະໂນມັດ]
iii. Household number ໝາຍເລກຄົວເຮືອນ		Number: ______ [drawn from sampling map] ໝາຍເລກ: ______
iv. Household coordinates ພິກັດເຮືອນ	a) Latitude ເສັ້ນຂະໜານ	[***coordinates entered automatically***] **[ພິກັດຖືກບັນທຶກໂດຍອັດຕະໂນມັດ]**
	b) Longitude ເສັ້ນແວງ	[***coordinates entered automatically***] **[ພິກັດຖືກບັນທຶກໂດຍອັດຕະໂນມັດ]**

Introduction

Hello, I'm a researcher working with the Ministry of Health, Ministry of Home Affair and together with the World Health Organisation. We are interested in the lives and health behaviours of villagers across Lao PDR. We are inviting all people above 16 years in the village to participate in our survey, so we better understand how villagers experience healthcare services. [provide PIS on request]

ສະບາຍດີ, ພວກເຮົາເປັນທີມນັກຄົ້ນຄວ້າ-ວິໄຈ ທີ່ເຮັດວຽກຮ່ວມກັນກັບ ກະຊວງສາທາລະນະສຸກ, ກະຊວງພາຍໃນ ພ້ອມກັນກັບ ອົງການອະນາໄມໂລກ ປະຈຳ ສ.ປ.ປ ລາວ. ພວກເຮົາໄດ້ໃຫ້ຄວາມສົນໃຈໃນການດຳລົງຊີວິດ ແລະ ສຸຂະພາບ ຂອງປະຊາຊົນໃນຂອບເຂດທົ່ວປະເທດ. ພວກເຮົາຂໍເຊີນຜູ້ທີ່ມີອາຍຸ 16 ປີ ຂຶ້ນໄປໃນກຸ່ມບ້ານເພື່ອເຂົ້າຮ່ວມໃນການສຳຫຼວດຂອງພວກເຮົາ, ເພື່ອຊ່ວຍໃຫ້ພວກເຮົາໄດ້ເຂົ້າໃຈເລິກເຊິ່ງກ່ຽວກັບປະສົບການໃນການເຂົ້າໃຊ້ບໍລິການ ຫຼື ຮັບການປິ່ນປົວທີ່ ສຸກສາລາ/ໂຮງໝໍເມືອງ ຂອງພວກທ່ານ. (ສະໜອງເອກະສານຂໍ້ມູນສຳລັບຜູ້ເຂົ້າຮ່ວມສຳພາດຕາມການຮ້ອງຂໍ)

Statement of consent (Verbal consent will be taken)

ຄຳຍິນຍອມເຂົ້າຮ່ວມການຄົ້ນຄວ້າ (ຜູ້ເຂົ້າຮ່ວມການຄົ້ນຄ້ວາຈະໄດ້ຮັບເອກະສານຂໍ້ມູນແລະບັນທຶກສຽງສະແດງຄວາມຍິນຍອມໃນການເຂົ້າຮ່ວມການຄົ້ນຄ້ວາ)
Thank you for participating. ຂໍຂອບໃຈທີ່ເຂົ້າຮ່ວມການສຳພາດກັບພວກເຮົາ.

v.Date of interview ວັນທີ່ສຳພາດ	[***date entered automatically***] [ວັນຖືກບັນທຶກໂດຍອັດຕະໂນມັດ]
vi.Time of interview begin ເວລາຂອງການສຳພາດ	[***time entered automatically***] [ເວລາຖືກບັນທຶກໂດຍອັດຕະໂນມັດ]
vii.Respondent name ຊື່ຂອງຜູ້ຖືກສຳພາດ	Respondent name: _____ ຊື່ຂອງຜູ້ຖືກສຳພາດ: _____
viii.Nickname ຊື່ຫຼິ້ນ	Nickname: _____ ຊື່ຫຼິ້ນ: ________
ix.Interviewer code ລະຫັດສຳພາດ	[***code entered automatically***] [ຂໍ້ມູນຖືກບັນທຶກໂດຍອັດຕະໂນມັດ]
x.[***Round 2 only:***] Confirm if respondent had been interviewed before ຢືນຢັນຖ້າຫາກຜູ້ຕອບແບບສອບຖາມເຄີຍຖືກສຳພາດມາກ່ອນແລ້ວ	Yes ແມ່ນ 1<br>No ບໍ່ແມ່ນ 0

Module I: Personal and Household Characteristics (Round I or new respondents in Round 2) (ການສຳຫຼວດຄັ້ງທີ 1 ຫຼື ຜູ້ຖືກສຳພາດກຸ່ມໃໝ່ໃນການສຳຫຼວດຄັ້ງທີ 2)
ພາກທີ 1: ຂໍ້ມູນສ່ວນຕົວແລະລັກສະນະຂອງຄົວເຮືອນ

1. Let us begin with a few questions about yourself and your household. ເຮົາຈະເລີ່ມຈາກຄຳຖາມກ່ຽວກັບທ່ານແລະຄົວເຮືອນຂອງທ່ານ

**1.1.**	[***record as observed***] Sex ບັນທຶກຈາກວິຈາລະນະຍານຂອງຜູ້ສຳພາດ] ເພດ	Female ຍິງ 1<br>Male ຊາຍ 0<br>Non-binary ບໍ່ລະບຸເພດ 2	
**1.2.**	How old are you? [*in years*] [***If respondent cannot give exact age, ask for approximate age***] ອາຍຸ [ປີ] [ຖ້າຜູ້ໃຫ້ສຳພາດບໍ່ສາມາດບອກອາຍຸຈິງໄດ້ ໃຫ້ຜູ້ສຳພາດປະມານອາຍຸຕົນເອງ]	Age in years: _______<br>ອາຍຸ [ປີ]: _______	
**1.3.**	Please indicate what kind of work you do. If you have more than one occupation at one time or throughout the year, please begin with the one in which you spend the most time and name up to three. If you do not have an occupation, please also mention whether you are still a student, retired, or unemployed.<br>ກະລຸນາບອກອາຊີບຂອງທ່ານ. ທ່ານເຮັດຫຼາຍກ່ວາໜຶ່ງອາຊີບໃນເວລາດຽວກັນ ຫຼື ພາຍໃນໄລຍະເວລາໜຶ່ງປີ, ກະລຸນາເລີ່ມຈາກ 3 ອາຊີບທີ່ທ່ານໃຊ້ເວລາເຮັດວຽກນັ້ນໆຫຼາຍທີ່ສຸດ. ຖ້າທ່ານບໍ່ມີອາຊີບ, ກະລຸນາບອກວ່າທ່ານເປັນນັກຮຽນ, ກະສຽນ ຫຼື ຫວ່າງງານ	a) Main occupation ອາຊີບຫຼັກ	Occupation: __<br>ອາຊີບ: ______
		b) Side occupation ອາຊີບເສີມ	Occupation: __<br>ອາຊີບ: ______
		c) Side occupation ອາຊີບເສີມ	Occupation: __<br>ອາຊີບ: ______
**1.4.**	What is your mother tongue? ພາສາທີ່ທ່ານໃຊ້ເປັນຫລັກແມ່ນພາສາຫຍັງ?	Mother tongue: ________________<br>ພາສາຫລັກ: ________________	
**1.5.**	Can you speak Lao? ທ່ານສາມາດເວົ້າພາສາລາວໄດ້ຫຼືບໍ່?	Yes ແມ່ນ 1<br>No ບໍ່ແມ່ນ 0	
**1.6.**	Can you read Lao? ທ່ານສາມາດອ່ານພາສາລາວໄດ້ ຫຼື ບໍ່?	Yes ແມ່ນ 1<br>No ບໍ່ແມ່ນ 0	

**1.7.** What is the highest grade of schooling that you completed? [***excluding informal education and pre-school education such as nursery and kindergarten, but including grade school, high school, vocational training, tertiary education, etc.***]<br>ລະດັບການສຶກສາສູງສຸດຂອງທ່ານແມ່ນຊັ້ນໃດ? [ບໍ່ນັບການສຶກສາກ່ອນລະບົບສາມັນ ເຊັ່ນ ໂຮງຮຽນລ້ຽງເດັກ ແລະ ອານຸບານ. ແຕ່ເລີ່ມນັບຕັ້ງແຕ່ລະດັບຊັ້ນປະຖົມ, ມັດທະຍົມ, ອາຊີວະສຶກສາ, ມະຫາວິທະຍາໄລ ແລະອື່ນໆ.]	Highest grade: ___<br>ລະດັບການສຶກສາສູງສຸດ: ____
**1.8.** Are you the head of your household? ທ່ານເປັນຫົວໜ້າຄົວເຮືອນນີ້ຫຼືບໍ່?	Yes ແມ່ນ 1<br>No ບໍ່ແມ່ນ 0<br>No, and HH head is not available 9<br>ບໍ່, ແລະ ຫົວໜ້າຄອບຄົວ ແມ່ນບໍ່ສະດວກໃນຂະນະນີ້
**1.8.1.** [*if yes to 1.8 or household head not available*] [**ຖ້າທ່ານຕອບ ແມ່ນໃນຂໍ້ 1.8 ຫຼື ຫົວໜ້າຄົວເຮືອນແມ່ນບໍ່ສະດວກໃນຂະນະນີ້**] How many adults and children (below 16) live in your household? ມີຜູ້ໃຫຍ່ ຫຼື ເດັກນ້ອຍ (ທີ່ອາຍຸຕ່ຳກວ່າ 16 ປີ) ຈັກຄົນທີ່ອາໄສຢູ່ໃນຄອບຄົວ ຂອງທ່ານ?	Number of adults: ___<br>ຈຳນວນ ຂອງ ຜູ້ໃຫຍ່ ___<br>Number of children: ___<br>ຈຳນວນ ຂອງ ເດັກນ້ອຍ ___
**1.8.2.** [*if yes to 1.8 or household head not available*] [**ຖ້າທ່ານຕອບ ແມ່ນໃນຂໍ້ 1.8 ຫຼື ຫົວໜ້າຄົວເຮືອນແມ່ນບໍ່ສະດວກໃນຂະນະນີ້**] How many rooms does your house have apart from toilet and hallways? ເຮືອນຂອງທ່ານມີຫ້ອງທັງໝົດຈັກຫ້ອງ ຖ້າບໍ່ນັບຫ້ອງນ້ຳແລະທາງເດີນ?	Number of rooms: ___<br>ຈຳນວນຫ້ອງ: _____
**1.8.3.** [*if yes to 1.8 or household head not available*] [**ຖ້າທ່ານຕອບ ແມ່ນໃນຂໍ້ 1.8 ຫຼື ຫົວໜ້າຄົວເຮືອນແມ່ນບໍ່ສະດວກໃນຂະນະນີ້**] I will now ask you for some items in your household. Please tell me…<br>ຕໍ່ໄປເຮົາຈະຖາມກ່ຽວກັບສິ່ງຂອງທີ່ທ່ານຄອບຄອງໃນຄົວເຮືອນ	Number of items in household:<br>ຈຳນວນສິ່ງຂອງທີ່ມີໃນຄົວເຮືອນ
**1.8.3.1.** Have you got a *functioning* **TV** in your household? If so, how many?<br>ທ່ານມີ**ໂທລະພາບ**ທີ່ໃຊ້ງານໄດ້ໃນຄົວເຮືອນຂອງທ່ານຫຼືບໍ່ ຖ້າມີ ມີຈັກໜ່ວຍ?	__
**1.8.3.2.** Have you got a *functioning* **rice cooker** in your household? If so, how many?<br>ທ່ານມີ**ໝໍ້ຫຸງເຂົ້າ**ທີ່ໃຊ້ງານໄດ້ໃນຄົວເຮືອນຂອງທ່ານຫຼືບໍ່ ຖ້າມີ ມີຈັກໜ່ວຍ?	__
**1.8.3.3.** Have you got a *functioning* **mobile phone** in your household? If so, how many?<br>ທ່ານມີ**ໂທລະສັບມືຖື, ແທັບເລັດ**ທີ່ໃຊ້ງານໄດ້ໃນຄົວເຮືອນຂອງທ່ານຫຼືບໍ່ ຖ້າມີ ມີຈັກໜ່ວຍ?	__
**1.8.3.4.** Have you got a *functioning* **computer** in your household? If so, how many?<br>ທ່ານມີ**ຄອມພິວເຕີ**ທີ່ໃຊ້ງານໄດ້ໃນຄົວເຮືອນຂອງທ່ານຫຼືບໍ່ ຖ້າມີ ມີຈັກໜ່ວຍ?	__
**1.8.3.5.** Have you got a *functioning* **scooter, motorcycle, or tricycle** in your household? If so, how many?<br>ທ່ານມີ**ລົດຈັກ, ລົດສາມລໍ້**ທີ່ໃຊ້ງານໄດ້ໃນຄົວເຮືອນຂອງທ່ານຫຼືບໍ່ ຖ້າມີ ມີຈັກຄັນ?	__
**1.8.3.6.** Have you got a *functioning* **car or truck** in your household? If so, how many?<br>ທ່ານມີ**ລົດໃຫຍ່ ຫຼື ລົດບັນທຸກ**ທີ່ໃຊ້ງານໄດ້ໃນຄົວເຮືອນຂອງທ່ານຫຼືບໍ່ ຖ້າມີ ມີຈັກຄັນ?	__
**1.8.3.7.** Have you got a *functioning* **refrigerator or freezer** in your household? If so, how many?<br>ທ່ານມີ**ຕູ້ເຢັນ ຫຼື ຕູ້ແຊ່**ທີ່ໃຊ້ງານໄດ້ໃນຄົວເຮືອນຂອງທ່ານຫຼືບໍ່ ຖ້າມີ ມີຈັກໜ່ວຍ?	__

**1.9.** What is your religion? ທ່ານນັບຖືສາສະໜາຫຍັງ?	No religion ບໍ່ນັບຖືສາສະໜາ	0
	Buddhist ພຸດ	1
	Christian ຄຣິດ	2
	Muslim ອິດສະລາມ	3
	Spirit / animism ນັບຖືຜີ	4
	Other (Specify) ______ ອື່ນໆ (ກະລຸນາບອກ) ____	5
	Don't know / prefer not to say ບໍ່ຮູ້ / ບໍ່ຢາກກ່າວ	99
**1.10.** What is your nationality? ສັນຊາດຂອງທ່ານແມ່ນຫຍັງ?	Lao ລາວ	1
	Thai ໄທ	2
	Myanmar/Burmese ມຽນມາ	3
	Vietnamese ຫວຽດນາມ	4
	Cambodian ກຳປູເຈຍ	5
	Chinese ຈີນ	6
	Other (Specify) ____ ອື່ນໆ (ກະລຸນາບອກ) _______	9
	Don't know / prefer not to say ບໍ່ຮູ້ / ບໍ່ຢາກກ່າວ	99
**1.11.** What is your ethnic background?<br>ຊົນເຜົ່າຂອງທ່ານແມ່ນຫຍັງ?	Lao Loum ລາວລຸ່ມ	1
	Tai-Thay ໄຕ-ໄທ	2
	Khmuic ຂະໝຸກ	3
	Palaungic ປາລວງຕິກ	4
	Vietic ຫວຽດຕິກ	5
	Katuic ກະຕອກ	6
	Bahnaric-Khmer ບານາລິກ-ຂະແມ	7
	Hmong ມົ້ງ	8
	Mien ມຽນ	9
	Tibeto-Buman ຕິເບດ-ພະມ້າ	10
	Other (Specify) ____ ອື່ນໆ (ກະລຸນາບອກ) ___	98
	Don't know / prefer not to say ບໍ່ຮູ້ / ບໍ່ຢາກກ່າວ	99
**1.12.** Do you have any physical disability?<br>ທ່ານມີຄວາມພິການທາງດ້ານຮ່າງກາຍ ຫຼື ບໍ່?	Yes ແມ່ນ	1
	No ບໍ່ແມ່ນ	0
	Don't know / prefer not to say ບໍ່ຮູ້ / ບໍ່ຢາກກ່າວ	9
**1.13.** [*if yes*] Can you please tell us what this condition/s is?<br>**[ຖ້າມີ]**<br>ທ່ານສາມາດບອກພວກເຮົາໄດ້ບໍ່ວ່າອາການເຫຼົ່ານີ້ແມ່ນຫຍັງ?	Condition/s: ______________________<br>ອາການ: _________________________	

**1.14.** Do you have any other chronic or long-term health conditions?<br>ທ່ານມີອາການຊໍາເຮື້ອ ຫຼື ບັນຫາສຸຂະພາບໄລຍະຍາວອື່ນໆກ່ຽວຂ້ອງບໍ່?	Yes ແມ່ນ 1<br>No ບໍ່ແມ່ນ 0<br>Don't know / prefer not to say ບໍ່ຮູ້ / ບໍ່ຢາກກ່າວ 9
**1.15.** [***if yes and different from 1.12***] Can you please tell us what this condition/s is?<br>**[ຖ້າແມ່ນ ແລະ ແຕກຕ່າງຈາກ ຂໍ້ 1.12]**<br>ທ່ານສາມາດບອກພວກເຮົາໄດ້ບໍ່ວ່າອາການເຫຼົ່ານັ້ນແມ່ນຫຍັງ?	Condition/s ອາການ: ______________________<br><br>[ອາການ] _________________________
**1.16.** Do you regularly smoke cigarettes, tobacco, or e-cigarettes? [***"regularly" means at least once a day***]<br>ທ່ານໄດ້ສູບຢາ, ຢາສູບ (ຢາເສັ້ນ), ຫຼື ຢາສູບໄຟຟ້າ ປົກກະຕິ ຫຼື ບໍ່? **["ປົກກະຕິ/ເປັນປະຈໍາ" ໝາຍເຖິງຢ່າງໜ້ອຍມື້ລະເທື່ອ]**	Yes **ແມ່ນ** 2<br>Yes, but not regularly **ແມ່ນ, ແຕ່ບໍ່ປົກກະຕິ/ເປັນປະຈໍາ** 1<br>No **ບໍ່ແມ່ນ** 0<br>Don't know **ບໍ່ຮູ້**/ prefer not to say **ບໍ່ຢາກກ່າວ** 9
**1.17.** Did the COVID-19 pandemic over the past two years change how you could use government healthcare like hospitals and health centres?<br>ການລະບາດຂອງເຊື້ອພະຍາດ COVID-19 ໃນສອງປີທີ່ຜ່ານມາໄດ້ໄດ້ປ່ຽນແປງ ຫຼື ສົ່ງຜົນກະທົບຕໍ່ການເຂົ້າຮັບການປິ່ນປົວສຸຂະພາບ ຫຼື ບໍ່ ເຊັ່ນ ໂຮງໝໍ (ໂຮງໝໍລັດ) ຫຼື ສຸກສາລາ/ໂຮງໝໍນ້ອຍ?	Yes, positively 2<br>No 1<br>Yes, negatively 0<br>Don't know **ບໍ່ຮູ້** / prefer not to say **ບໍ່ຢາກກ່າວ** 9
**1.18.** [***if yes***] Can you please tell us how?<br>**[ຖ້າກະທົບ]**<br>ທ່ານສາມາດບອກພວກເຮົາໄດ້ບໍ່ວ່າກະທົບທ່ານແບບໃດ?	Explanation **ອະທິບາຍ**: ______________________

Module II: Ownership, Trust, and Trust Landscape [*Rounds 1 and 2*] ພາກທີ II: ການເປັນເຈົ້າຂອງ, ຄວາມໄວ້ເນື້ອເຊື່ອໃຈ ແລະ ເຂດຄວາມໄວ້ເນື້ອເຊື່ອໃຈ [ຮອບ 1 ແລະ 2]

2. Thank you for this. Now we come to a part where I will ask you some questions about health and health providers around here.
ຂໍຂອບໃຈສໍາລັບການຕອບຄໍາຖາມນີ້. ຕໍ່ໄປແມ່ນພວກເຮົາຈະມາຖາມບາງຄໍາຖາມກ່ຽວກັບສຸຂະພາບ ແລະ ຜູ້ສະໜອງການປິ່ນປົວໃນຂອບເຂດນີ້

2.1. First, I would like to ask you what healthcare choices you would recommend for other villagers.
ກ່ອນອື່ນໝົດ, ພວກເຮົາຢາກຖາມທ່ານກ່ຽວກັບທາງເລືອກຂອງການປິ່ນປົວສຸຂະພາບແບບໃດທີ່ທ່ານຢາກແນະນໍາໃຫ້ສະມາຊິກໃນໝູ່ບ້ານ

**2.1.1.** Where would you recommend that pregnant women in your village receive care during their pregnancy? [***mark all that apply***]<br>ສະຖານທີ່ໃດທີ່ທ່ານຢາກແນະນໍາໃຫ້ແມ່ຍິງທີ່ກໍາລັງຖືພາໃນບ້ານຂອງທ່ານໄດ້ຮັບການປິ່ນປົວ/ຮັກສາ ໃນຊ່ວງທີ່ພວກເຂົາກໍາລັງຖືພາ?<br>**[ທ່ານສາມາດເລືອກໄດ້ຫຼາຍກວ່າ 1 ຂໍ້]**	Self-care (sleep, rest, medicine at home) ປິ່ນປົວດ້ວຍຕົນເອງ (ນອນ, ພັກຜ່ອນ, ກິນຢາຢູ່ບ້ານ) 1<br>Care from family and friends ຮັບການເບິ່ງແຍງຈາກຄອບຄົວ & ໝູ່ 2<br>Shop / vendor selling drugs ຮ້ານຄ້າ/ຮ້ານຂາຍຢາທົ່ວໄປ 3<br>Traditional healer ໝໍພື້ນເມືອງ/ໝໍຜີ 4<br>Pharmacist ແພດການຢາ/ຄົນຂາຍຢາ 5<br>Private clinic/hospital ຄຣີນິກເອກະຊົນ/ໂຮງໝໍ 6<br>NGO clinic/service ຄຣີນິກຂອງອົງການຈັດຕັ້ງສາກົນ/ການບໍລິການ 7<br>Health centre ສຸກສາລາ/ໂຮງໝໍນ້ອຍ 8<br>Public hospital ໂຮງໝໍໃຫຍ່ (ໂຮງໝໍລັດ)/<br>Military hospital ໂຮງໝໍທະຫານ 9<br>Village health volunteer ອາສາສະໝັກສາທາລະນະສຸກບ້ານ (ອສບ) 10<br>Unregistered/local/informal/retired doctor ໝໍບໍ່ມີໃບຢັ້ງຢືນແພດ/ທ້ອງຖິ່ນ/ບໍ່ເປັນທາງການ/ໝໍບໍານານ 11<br>Traditional birth assistant ໝໍຕໍາແຍ 15<br>Cross-border care/ປະເທດໃກ້ຄຽງ 97<br>Other (incl. internet; specify) ອື່ນໆ (ລວມເຖິງ ອິນເຕີເນັດ, ລະບຸ) ____ 98<br>Don't know / prefer not to say / no opinion 99<br>ບໍ່ຮູ້ / ບໍ່ຢາກກ່າວ / ບໍ່ມີຄໍາເຫັນ
**2.1.2.** Where would you recommend that pregnant women deliver their babies? [***if more than one possibility, select the strongest general preference***]<br>ທ່ານຈະແນະນໍາແມ່ຍິງຖືພາໃຫ້ເກີດລູກຢູ່ໃສ?<br>[ຖ້າຫາກມີຫຼາຍກວ່າໜຶ່ງຂໍ້, ກະລຸນາເລືອກຂໍ້ທີ່ຈະແນະນໍາທີ່ສຸດ]	Public hospital ໂຮງໝໍໃຫຍ່ (ໂຮງໝໍລັດ) 1<br>Health centre ສຸກສາລາ/ໂຮງໝໍນ້ອຍ 2<br>Private clinic ຄຣີນິກເອກະຊົນ 3<br>Their own home ບ້ານຂອງຕົນເອງ 4<br>Somebody else's home ບ້ານຂອງຄົນອື່ນ 5<br>Cross-border care/ປະເທດໃກ້ຄຽງ 97<br>Other (specify): ອື່ນໆ (ລະບຸ)_______________ 98<br>Don't know / prefer not to say / no opinion<br>ບໍ່ຮູ້ / ບໍ່ຢາກກ່າວ / ບໍ່ມີຄໍາເຫັນ 99

**2.1.3.** Where would you recommend that somebody with an injury gets treatment? [***mark all that apply***]<br>ທ່ານຈະແນະນຳຄົນທີ່ໄດ້ຮັບບາດເຈັບເຂົ້າຮັບການຮັກສາຢູ່ໃສ?<br>[ເລືອກຫຼາຍໄດ້ກວ່າໜຶ່ງຂໍ້]	Self-care (sleep, rest, medicine at home) ປິ່ນປົວດ້ວຍຕົນເອງ (ນອນ, ພັກຜ່ອນ, ກິນຢາຢູ່ບ້ານ) 1<br>Care from family and friends ຮັບການເບິ່ງແຍງຈາກຄອບຄົວ & ໝູ່ 2<br>Shop / vendor selling drugs ຮ້ານຄ້າ/ຮ້ານຂາຍຢາທົ່ວໄປ 3<br>Traditional healer ໝໍພື້ນເມືອງ/ໝໍຜີ 4<br>Pharmacist ແພດການຢາ/ຄົນຂາຍຢາ 5<br>Private clinic/hospital ຄຣີນິກເອກະຊົນ/ໂຮງໝໍ 6<br>NGO clinic/service ຄຣີນິກຂອງອົງການຈັດຕັ້ງສາກົນ/ການບໍລິການ 7<br>Health centre ສຸກສາລາ/ໂຮງໝໍນ້ອຍ 8<br>Public hospital ໂຮງໝໍໃຫຍ່ (ໂຮງໝໍລັດ)/<br>Military hospital ໂຮງໝໍທະຫານ 9<br>Village health volunteer ອາສາສະໝັກສາທາລະນະສຸກບ້ານ (ອສບ) 10<br>Unregistered/local/informal/retired doctor<br>ໝໍບໍ່ມີໃບຢັ້ງຢືນແພດ/ທ້ອງຖິ່ນ/ບໍ່ເປັນທາງການ/ໝໍບຳນານ 11<br>Traditional birth assistant ໝໍຕຳແຍ 15<br>Cross-border care/ປະເທດໃກ້ຄຽງ 97<br>Other (incl. internet; specify) ອື່ນໆ (ລວມເຖິງ ອິນເຕີເນັດ, ລະບຸ) ____ 98<br>Don't know / prefer not to say / no opinion 99<br>ບໍ່ຮູ້ / ບໍ່ຢາກກ່າວ / ບໍ່ມີຄຳເຫັນ
**2.1.4.** Where would you recommend that somebody who has COVID gets treatment? [***mark all that apply***]<br>ທ່ານຈະແນະນຳໃຫ້ຄົນເປັນພະຍາດໂຄວິດເຂົ້າຮັບການປິ່ນປົວຢູ່ໃສ ? [ເລືອກໄດ້ຫຼາຍກວ່າໜຶ່ງຂໍ້]	Self-care (sleep, rest, medicine at home) ປິ່ນປົວດ້ວຍຕົນເອງ (ນອນ, ພັກຜ່ອນ, ກິນຢາຢູ່ບ້ານ) 1<br>Care from family and friends ຮັບການເບິ່ງແຍງຈາກຄອບຄົວ & ໝູ່ 2<br>Shop / vendor selling drugs ຮ້ານຄ້າ/ຮ້ານຂາຍຢາທົ່ວໄປ 3<br>Traditional healer ໝໍພື້ນເມືອງ/ໝໍຜີ 4<br>Pharmacist ແພດການຢາ/ຄົນຂາຍຢາ 5<br>Private clinic/hospital ຄຣີນິກເອກະຊົນ/ໂຮງໝໍ 6<br>NGO clinic/service ຄຣີນິກຂອງອົງການຈັດຕັ້ງສາກົນ/ການບໍລິການ 7<br>Health centre ສຸກສາລາ/ໂຮງໝໍນ້ອຍ 8<br>Public hospital ໂຮງໝໍໃຫຍ່ (ໂຮງໝໍລັດ)/<br>Military hospital ໂຮງໝໍທະຫານ 9<br>Village health volunteer ອາສາສະໝັກສາທາລະນະສຸກບ້ານ (ອສບ) 10<br>Unregistered/local/informal/retired doctor<br>ໝໍບໍ່ມີໃບຢັ້ງຢືນແພດ/ທ້ອງຖິ່ນ/ບໍ່ເປັນທາງການ/ໝໍບຳນານ 11<br>Traditional birth assistant ໝໍຕຳແຍ 15<br>Cross-border care/ປະເທດໃກ້ຄຽງ 97<br>Other (incl. internet; specify) ອື່ນໆ (ລວມເຖິງ ອິນເຕີເນັດ, ລະບຸ) ____ 98<br>Don't know / prefer not to say / no opinion 99<br>ບໍ່ຮູ້ / ບໍ່ຢາກກ່າວ / ບໍ່ມີຄຳເຫັນ

2.2. I will now ask you some questions about the local health centre here in the village.
ພວກເຮົາຈະຖາມບາງຄຳຖາມກ່ຽວກັບສຸກສາລາ/ໂຮງໝໍນ້ອຍໃນບ້ານຂອງທ່ານ

**2.2.1.** Are you aware that there is a health centre that serves this village?<br>ທ່ານຮູ້ບໍ່ວ່າມັນມີສຸກສາລາ/ໂຮງໝໍນ້ອຍໃນໝູ່ບ້ານແຫ່ງນີ້ ?	Yes ແມ່ນ<br>No ບໍ່ແມ່ນ<br>Don't know / prefer not to say ບໍ່ຮູ້ / ບໍ່ຢາກກ່າວ	1<br>0<br>9
**2.2.2.** [***If yes***] Have you ever been to the health centre?<br>ທ່ານເຄີຍໄປສຸກສາລາ ຫຼື ໂຮງໝໍນ້ອຍບໍ່?	Yes ແມ່ນ<br>No ບໍ່ແມ່ນ<br>Don't know / prefer not to say ບໍ່ຮູ້ / ບໍ່ຢາກກ່າວ	1<br>0<br>9

2.2.3. [***If 2.2.1 is yes***] I will now ask you a few questions to better understand your impression of the health centre. It does not matter if you have been to the health centre or not; we are interested in what you think and believe and what you have heard, even if it is not based on personal experience. ຕໍ່ໄປນີ້ ຂ້າພະເຈົ້າຈະຖາມຄຳຖາມຈຳນວນໜຶ່ງ ເພື່ອເຂົ້າໃຈເຖິງຄວາມປະທັບໃຈຂອງທ່ານຕໍ່ກັບສຸກສາລາ/ໂຮງໝໍນ້ອຍ. ບໍ່ຈຳເປັນວ່າທ່ານເຄີຍໃຊ້ບໍລິການສຸກສາລາ/ໂຮງໝໍນ້ອຍມາກ່ອນຫຼືບໍ່; ພວກເຮົາສົນໃຈໃນສິ່ງທ່ານຄິດ ແລະ ຄວາມເຊື່ອ ແລະ ເຄີຍໄດ້ຍິນມາ, ເຖິງແມ້ວ່າຈະບໍ່ໄດ້ອີງຕາມປະສົບການສ່ວນຕົວກໍຕາມ.

**2.2.3.1.** Can you talk comfortably with the staff at the health centre?<br>ທ່ານສາມາດລົມກັບພະນັກງານແພດປະຈຳໂຮງໝໍນ້ອຍໄດ້ຢ່າງເປັນກັນເອງ ແລະ ສະບາຍໃຈບໍ່?	Yes ແມ່ນ 1<br>No ບໍ່ແມ່ນ 0<br>Don't know / prefer not to say ບໍ່ຮູ້ / ບໍ່ຢາກກ່າວ 9
**2.2.3.2.** Can you give me an example of the kind of stories you heard about the health centre or its staff? (give examples through the answer categories)<br>ຈົ່ງຍົກຕົວຢ່າງເລື່ອງລາວທີ່ທ່ານເຄີຍໄດ້ຍິນມາກ່ຽວກັບສຸກສາລາ/"ໂຮງໝໍນ້ອຍ ຫຼື ກ່ຽວກັບພະນັກງານແພດ-ໝໍໃຫ້ຟັງໄດ້ບໍ່? (ໃຫ້ຕົວຢ່າງຜ່ານແຕ່ລະປະເພດຂອງຄຳຕອບ)	Bad (scolding, mean, extracting money, refusing service, closed when needed) ບໍ່ດີ (ຮ້ອງໃສ່, ໃຈຮ້າຍ, ຖາມເອົາເງິນ, ປະຕິເສດການປິ່ນປົວ, ປິດເມື່ອຕ້ອງການ 0<br>Neutral (descriptive, services being provided, location, opening times) ທຳມະດາ (ໄດ້ຮັບການບໍລິການ, ເວລາເປີດປົກກະຕິ, ການອະທິບາຍດີ) 1<br>Good (caring staff, solving problems, supportive, friendly) ດີ (ໝໍດູແລດີ, ແກ້ໄຂບັນຫາໄດ້, ສະໜັບສະໜູນ, ເປັນກັນເອງ) 2<br>Don't know / prefer not to say ບໍ່ຮູ້ / ບໍ່ຢາກກ່າວ 9<br>Story ອະທິບາຍເລື່ອງລາວ:<br>________________________________

**2.2.3.3.** Is the health centre a place where staff treat you badly?<br>ສຸກສາລາ/ໂຮງໝໍນ້ອຍແມ່ນສະຖານທີ່ທີ່ພະນັກງານປະຕິບັດກັບທ່ານບໍ່ດີ/ເອົາບໍ່ມ່ອນບໍ່?	Yes ແມ່ນ 1<br>No ບໍ່ແມ່ນ 0<br>Don't know / prefer not to say ບໍ່ຮູ້ / ບໍ່ຢາກກ່າວ 9
**2.2.3.4.** Have you ever experienced/ heard of any kind of discrimination at the health centre? ທ່ານເຄີຍມີປະສົບການ ຫຼື ໄດ້ຍິນກ່ຽວກັບການຈຳແນກການບໍລິການ/ເລືອກປະຕິບັດໃນໂຮງໝໍນ້ອຍບໍ່?	Yes ແມ່ນ 1<br>No ບໍ່ແມ່ນ 0<br>Don't know / prefer not to say ບໍ່ຮູ້ / ບໍ່ຢາກກ່າວ 9
2.2.3.4.1. [*Round 2 only and if yes*] Was this the case in the last three months?<br>[ຮອບສອງເທົ່ານັ້ນ] ມັນແມ່ນກໍລະນີທີ່ເກີດຂຶ້ນໃນໄລຍະສາມເດືອນທີ່ຜ່ານມາ?	Yes ແມ່ນ 1<br>No ບໍ່ແມ່ນ 0<br>Don't know / prefer not to say ບໍ່ຮູ້ / ບໍ່ຢາກກ່າວ 9
2.2.3.4.2. [***all Round 1 plus < Round 2 and if yes>***] Example if yes<br>ຖ້າແມ່ນຊ່ວຍຍົກຕົວຢ່າງ	Example if yes ຖ້າແມ່ນຊ່ວຍຍົກຕົວຢ່າງ:<br>______________________________
**2.2.3.5.** Have you ever been refused/ heard people being refused treatment from the staff at the health centre? ແພດໝໍເຄີຍປະຕິເສດການປິ່ນປົວ ຫຼື ເລືອກປະຕິບັດກັບທ່ານ ຫຼື ຄົນທີ່ທ່ານຮູ້ຈັກຫຼືບໍ່	Yes ແມ່ນ 1<br>No ບໍ່ແມ່ນ 0<br>Don't know / prefer not to say ບໍ່ຮູ້ / ບໍ່ຢາກກ່າວ 9
2.2.3.5.1. [*Round 2 only and if yes*] Was this the case in the last three months?<br>[ຮອບສອງເທົ່ານັ້ນ]<br>ມັນແມ່ນກໍລະນີທີ່ເກີດຂຶ້ນໃນໄລຍະສາມເດືອນທີ່ຜ່ານມາ?	Yes ແມ່ນ 1<br>No ບໍ່ແມ່ນ 0<br>Don't know / prefer not to say ບໍ່ຮູ້ / ບໍ່ຢາກກ່າວ 9
**2.2.3.6.** [***all Round 1 plus < Round 2 and if yes>***] If yes, what was the reason? ຖ້າເຄີຍ, ເຫດຜົນຍ້ອນຫຍັງ?	Response: ______________________________
**2.2.3.7.** If you went to the health centre right at this moment with a motorbike accident, do you think they would be able to provide you with primary treatment or first aid? ຖ້າຫາກທ່ານໄປສຸກສາລາ/ໂຮງໝໍນ້ອຍ ເມື່ອຕອນເກີດອຸບັດຕິເຫດລົດຈັກ ທ່ານຄິດວ່າພະນັກງານແພດໂຮງໝໍນ້ອຍ/ໂຮງໝໍເມືອງຈະສາມາດສະໜອງການປິ່ນປົວຂັ້ນທຳອິດໃຫ້ທ່ານໄດ້ ຫຼື ບໍ່?	Yes ແມ່ນ 1<br>No ບໍ່ແມ່ນ 0<br>Don't know / prefer not to say ບໍ່ຮູ້ / ບໍ່ຢາກກ່າວ 9
**2.2.3.8.** Do you feel confident in their medical skills?<br>ທ່ານຮູ້ສຶກໝັ້ນໃຈໃນຄວາມຊຳນິຊຳນານ/ປະສົບການໃນການປິ່ນປົວຂອງພະນັກງານແພດປະຈຳໂຮງໝໍນ້ອຍບໍ່?	Yes (confidently) ແມ່ນ (ຢ່າງໝັ້ນໃຈ) 1<br>So-so / no strong opinion ທຳມະດາ/ປານກາງ 3<br>No ບໍ່ແມ່ນ 0<br>Don't know / prefer not to say ບໍ່ຮູ້ / ບໍ່ຢາກກ່າວ 9
**2.2.3.9.** [DELETED]	
**2.2.3.10.** Do health staff ask for money for extra services, ask for money for services which should be free or to buy medicines from their houses?<br>ພະນັກງານສຸກສາລາໄດ້ຖາມເອົາເງິນສຳລັບການບໍລິການພິເສດບໍ່,<br>ຖາມເອົາເງິນຈາກການບໍລິການທີ່ບໍ່ຄວນເສຍຄ່າ ຫຼື<br>ພະນັກງານແພດເຄີຍແນະນຳໃຫ້ໄປຊື້ຢາທີ່ບ້ານຂອງເຂົາຫຼືບໍ່?	Yes ແມ່ນ 1<br>No ບໍ່ແມ່ນ 0<br>Don't know / prefer not to say ບໍ່ຮູ້ / ບໍ່ຢາກກ່າວ 9
2.2.3.10a. Explain if there is anything unusual to add (e.g. felt need to offer money or gifts for better care, or if you don't give extra money, do you get bad care?)<br>ອະທິບາຍຖ້າມີສິ່ງທີ່ຢາກເພີ່ມຕື່ມ:(ຕົວຢ:<br>ຮູ້ສຶກວ່າຕ້ອງໄດ້ເອົາເງິນໃຫ້ທ່ານໝໍຈຶ່ງຈະໄດ້ຮັບການບໍລິການດີຂຶ້ນ, ຫຼື ຖ້າບໍ່ໃຫ້ເງິນ,<br>ຈະໄດ້ຮັບການບໍລິການທີ່ບໍ່ດີ?	Explain if there is anything unusual to add<br>ອະທິບາຍຖ້າມີສິ່ງທີ່ຢາກເພີ່ມຕື່ມ: __________
2.2.3.10b. if you don't give extra money, do you get bad care?<br>ຖ້າທ່ານບໍ່ໄດ້ເອົາເງິນໃຫ້, ທ່ານຈະຖືກດູແລບໍ່ດີບໍ່?	Yes ແມ່ນ 1<br>No ບໍ່ແມ່ນ 0<br>Don't know / prefer not to say ບໍ່ຮູ້ / ບໍ່ຢາກກ່າວ 9
**2.2.3.11.** Overall, would you say you rather trust or distrust the healthcare centre for your personal healthcare? ໂດຍລວມແລ້ວ, ທ່ານມີຄວາມໄວ້ເນື້ອເຊື່ອໃຈໃນການເຂົ້າຮັບການບໍລິການປິ່ນປົວ/ດູແລສຸຂະພາບສ່ວນຕົວຂອງທ່ານກັບສຸກສາລາ/ໂຮງໝໍນ້ອຍ ຫຼື ບໍ່?	Rather trust ໄວ້ເນື້ອເຊື່ອໃຈ 2<br>Neutral / no opinion ໄວ້ເນື້ອເຊື່ອໃຈປານກາງ/ບໍ່ມີຄຳເຫັນ 1<br>Rather distrust ບໍ່ໄວ້ເນື້ອເຊື່ອໃຈ 0<br>Don't know / prefer not to say ບໍ່ຮູ້ / ບໍ່ຢາກກ່າວ 9

2.3. Let us know have a look at healthcare in your village more generally. ຕໍ່ໄປນີ້ ເຮົາຈະມາເບິ່ງສຸກສາລາ/ໂຮງໝໍນ້ອຍໃນໝູ່ບ້ານຂອງທ່ານ ຫຼື ໂດຍທົ່ວໄປ

**2.3.1.** Have healthcare services improved or worsened in the last three months?<br>ການບໍລິການຂອງສຸກສາລາ ຫຼື ໂຮງໝໍນ້ອຍໄດ້ປ່ຽນໄປໃນທາງທີ່ດີຂຶ້ນ ຫຼື ບໍ່ດີລົງກ່ອນເກົ່າໃນໄລຍະສາມເດືອນທີ່ຜ່ານມາ?	Improved ພັດທະນາຂຶ້ນ 2<br>No change / no opinion ບໍ່ມີການປ່ຽນແປງ/ບໍ່ມີຄຳຄິດເຫັນ 1<br>Worsened ບໍ່ດີກ່ອນເກົ່າ 0<br>Don't know / prefer not to say ບໍ່ຮູ້ / ບໍ່ຢາກກ່າວ 9
**2.3.2.** What is the most important change in healthcare in the village that you observed?<br>ການປ່ຽນແປງທີ່ສຳຄັນທີ່ສຸດໃນສຸກສາລາ/ໂຮງໝໍນ້ອຍໃນໝູ່ບ້ານທີ່ທ່ານສັງເກດເຫັນແມ່ນຫຍັງ?	Specify ລະບຸເຈາະຈົງ : ______________________
**2.3.3.** What do you think should improve most urgently?<br>ທ່ານຄິດວ່າສິ່ງໃດທີ່ຄວາມປັບປຸງໂດຍຮີບດ່ວນທີ່ສຸດ?	Specify ລະບຸເຈາະຈົງ : ______________________

2.4. Next, I would like to ask just a few questions specifically about your involvement in healthcare service provision in this village
ຕໍ່ໄປ, ພວກເຮົາຈະມາຖາມຕື່ມ 2-3 ຄຳຖາມ ທີ່ສະເພາະເຈາະຈົງກ່ຽວກັບການມີສ່ວນຮ່ວມຂອງທ່ານໃນການປັບປຸງບໍລິການສຸຂະພາບໃນໝູ່ບ້ານ

**2.4.1.** a) Do you think that you have a role in making this a “healthy village”?<br>ທ່ານຄິດວ່າທ່ານມີໜ້າທີ່ໃນການເຮັດໃຫ້ “ຊຸມຊົນມີສຸຂະພາບດີ” ຫຼືບໍ່?	Yes ແມ່ນ 1<br>No ບໍ່ແມ່ນ 0<br>Don't know / prefer not to say ບໍ່ຮູ້ / ບໍ່ຢາກກ່າວ 9
**2.4.2.** a) Do you think that you can contribute to making the village healthy?<br>ທ່ານຄິດວ່າທ່ານສາມາດປະກອບສ່ວນໃນການເຮັດໃຫ້ “ຊຸມຊົນມີສຸຂະພາບດີ” ຫຼືບໍ່?	Yes ແມ່ນ 1<br>No ບໍ່ແມ່ນ 0<br>Don't know / prefer not to say ບໍ່ຮູ້ / ບໍ່ຢາກກ່າວ 9
**2.4.3.** a) Would it be important for you if you could do that? ມັນຈະສຳຄັນກັບທ່ານຫຼືບໍ່ ຖ້າຫາກທ່ານສາມາດເຮັດໃຫ້ “ຊຸມຊົນມີສຸຂະພາບດີ” ໄດ້?	Yes ແມ່ນ 1<br>No ບໍ່ແມ່ນ 0<br>Don't know / prefer not to say ບໍ່ຮູ້ / ບໍ່ຢາກກ່າວ 9
2.4.1.b) Do you think that you have a role in making the health centre services better? ທ່ານຄິດວ່າທ່ານມີໜ້າທີ່ໃນການປະກອບສ່ວນເຮັດໃຫ້ການບໍລິການສຸຂະພາບຢູ່ສຸກສາລາດີຂຶ້ນ ຫຼືບໍ່?	Yes ແມ່ນ 1<br>No ບໍ່ແມ່ນ 0<br>Don't know / prefer not to say ບໍ່ຮູ້ / ບໍ່ຢາກກ່າວ 9
2.4.2.b) Do you think that you can contribute to making the health centre services better?<br>ທ່ານຄິດວ່າທ່ານສາມາດປະກອບສ່ວນໃນການເຮັດໃຫ້ການບໍລິການຢູ່ສຸກສາລາດີຂຶ້ນຫຼືບໍ່?	Yes ແມ່ນ 1<br>No ບໍ່ແມ່ນ 0<br>Don't know / prefer not to say ບໍ່ຮູ້ / ບໍ່ຢາກກ່າວ 9
2.4.3.b) Would it be important for you if you could do that? ມັນຈະສຳຄັນກັບທ່ານຫຼືບໍ່ ຖ້າຫາກທ່ານສາມາດປະກອບສ່ວນໃນການເຮັດໃຫ້ການບໍລິການຢູ່ສຸກສາລາດີຂຶ້ນໄດ້?	Yes ແມ່ນ 1<br>No ບໍ່ແມ່ນ 0<br>Don't know / prefer not to say ບໍ່ຮູ້ / ບໍ່ຢາກກ່າວ 9
**2.4.4.** Based on your impressions and experiences, do you think that the health centre offers the kind of care that this community needs? ອີງຕາມຄວາມປະທັບໃຈ ແລະ ປະສົບການຂອງທ່ານ,<br>ທ່ານຄິດວ່າສຸກສາລາ/ໂຮງໝໍນ້ອຍສະໜອງປະເພດຂອງການປິ່ນປົວທີ່ຊຸມຊົນນີ້ຕ້ອງການຫຼືບໍ່?	Yes ແມ່ນ 1<br>No ບໍ່ແມ່ນ 0<br>Don't know / prefer not to say ບໍ່ຮູ້ / ບໍ່ຢາກກ່າວ 9
**2.4.5.** Can you give me an example for your response to the previous question?<br>ທ່ານຈົ່ງຍົກຕົວຢ່າງຕໍ່ກັບຄຳຕອບຂອງທ່ານຂອງຄຳຖາມກ່ອນໜ້ານີ້ໄດ້ບໍ່?	Example: ___________________________
**2.5.** Next, I would like to ask just a few questions about other people’s involvement in healthcare service provision in this village. ຕໍ່ໄປ, ຂ້າພະເຈົ້າຂໍຖາມຕື່ມກ່ຽວກັບການມີສ່ວນຮ່ວມຂອງປະຊາຊົນໃນການໃຫ້ບໍລິການຂອງສຸກສາລາໃຫ້ບ້ານຂອງທ່ານ	
**2.5.1.** Do you know the name of the village chief or the deputy?<br>ທ່ານຮູ້ຈັກຊື່ນາຍບ້ານ ຫຼື ຮອງນາຍບ້ານບໍ່?	Yes ແມ່ນ 1<br>No ບໍ່ແມ່ນ 0<br>Not applicable ບໍ່ແມ່ນທັງໝົດ 8<br>Don't know / prefer not to say ບໍ່ຮູ້ / ບໍ່ຢາກກ່າວ 9
**2.5.2.** Do you know the name of the Village Health Volunteer?<br>ທ່ານຮູ້ຈັກຊື່ຂອງຫົວໜ້າທີມອາສາສະໝັກສາທາລະນະສຸກບ້ານ (ອສບ)<br>ໃນໝູ່ບ້ານບໍ່?	Yes ແມ່ນ 1<br>No ບໍ່ແມ່ນ 0<br>Not applicable ບໍ່ແມ່ນທັງໝົດ 8<br>Don't know / prefer not to say ບໍ່ຮູ້ / ບໍ່ຢາກກ່າວ 9
**2.5.3.** Do you know the name of the Women’s Union in the village?<br>ທ່ານຮູ້ຈັກຫົວໜ້າສະຫະພັນແມ່ຍິງໃນໝູ່ບ້ານບໍ່?	Yes ແມ່ນ 1<br>No ບໍ່ແມ່ນ 0<br>Not applicable ບໍ່ແມ່ນທັງໝົດ 8<br>Don't know / prefer not to say ບໍ່ຮູ້ / ບໍ່ຢາກກ່າວ 9
**2.5.4.** Do you know the name of the Lao Front in the village?<br>ທ່ານຮູ້ຈັກຊື່ຂອງຫົວໜ້າແນວລາວສ້າງຊາດໃນໝູ່ບ້ານບໍ່?	Yes ແມ່ນ 1<br>No ບໍ່ແມ່ນ 0<br>Not applicable ບໍ່ແມ່ນທັງໝົດ 8<br>Don't know / prefer not to say ບໍ່ຮູ້ / ບໍ່ຢາກກ່າວ 9
**2.5.5.** Do you know the name of the Lao Youth Union in the village?<br>ທ່ານຮູ້ຈັກຊື່ຂອງຫົວໜ້າສູນກາງຊາວໜຸ່ມໃນໝູ່ບ້ານບໍ່?	Yes ແມ່ນ 1<br>No ບໍ່ແມ່ນ 0<br>Not applicable ບໍ່ແມ່ນທັງໝົດ 8<br>Don't know / prefer not to say ບໍ່ຮູ້ / ບໍ່ຢາກກ່າວ 9
**2.5.6.** [***if yes to 2.5.1-2.5.5***] Have you seen any of the people in this village taking action for community health in past 3 months?<br>[**ຖ້າແມ່ນ ໃນຄຳຖາມ *2.5.1-2.5.5***]<br>ທ່ານເຄີຍເຫັນບຸກຄົນເຫຼົ່ານີ້ໃນໝູ່ບ້ານລົງມືປະຕິບັດເພື່ອສ້າງຊຸມຊົນໃຫ້ມີສຸຂະພາບດີ ພາຍໃນໄລຍະເວລາ 3 ເດືອນທີ່ຜ່ານມາບໍ່?	Village chief / deputy ນາຍບ້ານ/ຮອງນາຍບ້ານ 1<br>Village health volunteer ອາສາສະໝັກສາທາລະນະສຸກບ້ານ (ອສບ 2<br>Lao Women’s Union ສະຫະພັນແມ່ຍິງ 3<br>Lao Front ແນວລາວສ້າງຊາດ 4<br>Lao Youth Union ສູນກາງຊາວໜຸ່ມ 5<br>No one ບໍ່ມີໃຜ 8<br>Don’t know / prefer not to say ບໍ່ຮູ້ / ບໍ່ຢາກກ່າວ 9
**2.5.7.** [***if yes to any in 2.5.6***] Can you explain what they did? [**ຖ້າແມ່ນ ໃນຂໍ້ 2.5.6]**<br>ທ່ານຊ່ວຍອະທິບາຍສິ່ງທີ່ເຂົາເຮັດໄດ້ບໍ່?	Explanation ອະທິບາຍ: ______________________
**Module III: CONNECT Workshop** [***Round 2 only***] ພາກທີ III: ກອງປະຊຸມ CONNECT [ຮອບສອງເທົ່ານັ້ນ]<br>**3.** A 2-day workshop took place three months ago in your village, involving healthcare workers and villagers. ກອງປະຊຸມ 2 ມື້ ເກີດຂຶ້ນສາມເດືອນກ່ອນໜ້ານີ້ໃນໝູ່ບ້ານຂອງທ່ານ, ມີສ່ວນຮ່ວມໂດຍແພດໝໍ ແລະ ຊາວບ້ານ.	

**3.1.**	Are you aware of this workshop? [***provide slight help if respondent is not entirely sure***]<br>ທ່ານຮູ້ຈັກກອງປະຊຸມນີ້ບໍ່? [ຊ່ວຍຜູ້ຕອບແບບສອບຖາມເມື່ອບໍ່ແນ່ໃຈ]	Yes ແມ່ນ 1<br>No ບໍ່ແມ່ນ 0<br>Don't know / prefer not to say ບໍ່ຮູ້ / ບໍ່ຢາກກ່າວ 9
**3.2.**	[***if yes to 3.1***] Were you invited to participate in the workshop?<br>**[ຖ້າທ່ານຕອບ ແມ່ນ ໃນຂໍ້ 3.1]**<br>ທ່ານໄດ້ຖືກເຊີນໃຫ້ເຂົ້າຮ່ວມໃນງານກອງປະຊຸມ ຫຼື ບໍ່?	Yes ແມ່ນ 1<br>No ບໍ່ແມ່ນ 0<br>Don't know / prefer not to say ບໍ່ຮູ້ / ບໍ່ຢາກກ່າວ 9
**3.3.**	[***if yes to 3.1***] Did you attend the activities?<br>**[ຖ້າທ່ານຕອບ ແມ່ນ ໃນຂໍ້ 3.1]**<br>ທ່ານໄດ້ເຂົ້າຮ່ວມການເຮັດກິດຈະກຳເຫຼົ່ານັ້ນ ຫຼື ບໍ່?	Yes ແມ່ນ 2<br>Yes, but not throughout ເຂົ້າຮ່ວມ, ແຕ່ບໍ່ໄດ້ເຂົ້າຮ່ວມທຸກກິດຈະກຳ 1<br>No ບໍ່ແມ່ນ 0<br>Don't know / prefer not to say ບໍ່ຮູ້ / ບໍ່ຢາກກ່າວ 9
**3.4.**	[***if yes / yes but to 3.3***] Did you feel included in the activity?<br>**[ຖ້າທ່ານຕອບ ແມ່ນ ແຕ່ໃນຂໍ້ 3.3]**<br>ທ່ານຮູ້ສຶກມີສ່ວນຮ່ວມໃນກິດຈະກຳນັ້ນ ຫຼື ບໍ່?	Yes ແມ່ນ 1<br>No ບໍ່ແມ່ນ 0<br>Don't know / prefer not to say ບໍ່ຮູ້ / ບໍ່ຢາກກ່າວ 9
**3.5.**	[***if yes / yes but to 3.3***] Did you have the chance to contribute to the conversations?<br>**ຖ້າທ່ານຕອບ ແມ່ນ ແຕ່ໃນຂໍ້ 3.3]**<br>ທ່ານໄດ້ມີໂອກາດປະກອບສ່ວນໃນການສົນທະນາເຫຼົ່ານັ້ນ ຫຼື ບໍ່?	Yes ແມ່ນ 1<br>No ບໍ່ແມ່ນ 0<br>Don't know / prefer not to say ບໍ່ຮູ້ / ບໍ່ຢາກກ່າວ 9
**3.6.**	[***if yes / yes but to 3.3***] Did you feel that you were involved in the decisions of the workshop?<br>**ຖ້າທ່ານຕອບ ແມ່ນ ແຕ່ໃນຂໍ້ 3.3]**<br>ທ່ານຮູ້ສຶກວ່າທ່ານໄດ້ມີສ່ວນຮ່ວມໃນການຕັດສິນໃຈ ໃນກອງປະຊຸມນັ້ນ ຫຼື ບໍ່?	Yes ແມ່ນ 1<br>No ບໍ່ແມ່ນ 0<br>Don't know / prefer not to say ບໍ່ຮູ້ / ບໍ່ຢາກກ່າວ 9
**3.7.**	[***if yes to 3.1***] What, in your opinion, was the activity about?<br>**[ຖ້າທ່ານຕອບ ແມ່ນ ໃນຂໍ້ 3.1]** ໃນຄວາມຄິດເຫັນຂອງທ່ານ, ທ່ານຄິດວ່າກິດຈະກຳນີ້ແມ່ນຈັດກ່ຽວກັບຫຍັງ?	Response ຕອບ: ___________________________________
**3.8.**	Have you heard about the orange tree, decisions, or an action plan? [***mark all that apply***]<br>ທ່ານເຄີຍໄດ້ຍິນກ່ຽວກັບຕົ້ນໄມ້ຈຸດປະສົງ, ການຕັດສິນໃຈ, ຫຼື ແຜນວຽກຕົວຈິງບໍ່? **[ທ່ານສາມາດເລືອກໄດ້ຫຼາຍກວ່າ 1 ຂໍ້]**	Have not heard about it ບໍ່ເຄີຍໄດ້ຍິນມາກ່ອນ 0<br>Orange tree ຕົ້ນໄມ້ຈຸດປະສົງ/ oranges ຈຸດປະສົງ 1<br>Activity decisions ການຕັດສິນໃຈໃນກິດຈະກຳ 2<br>Action plan ແຜນວຽກຕົວຈິງ 3<br>Other description of action plan ຄວາມໝາຍອື່ນໆ ຂອງ ແຜນວຽກຕົວຈິງ 4<br>Don't know / prefer not to say ບໍ່ຮູ້ / ບໍ່ຢາກກ່າວ 9
**3.9.**	[***if 3.8 is 1-4***] What, in your opinion, does the orange tree/decisions / action plan say? [ຖ້າ ຂໍ້ 3.8 ແມ່ນ ຕົວເລືອກ 1-4]<br>ໃນຄວາມຄິດເຫັນຂອງທ່ານ ຕົ້ນໄມ້ຈຸດປະສົງ/ການຕັດສິນໃຈ/ແຜນການປະຕິບັດວຽກຕົວຈິງ ມີຄວາມໝາຍວ່າແນວໃດ?	Response ຄຳຕອບ: ___________________________________
**3.10.**	[***if 3.8 is 1-4***] Have you seen any of these actions being implemented?<br>[ຖ້າ ຂໍ້ 3.8 ແມ່ນ ຕົວເລືອກ 1-4]<br>ທ່ານເຄີຍພົບເຫັນກິດຈະກຳໃດໜຶ່ງນີ້ຖືກນຳໄປປະຕິບັດຕົວຈິງບໍ່?	Yes ແມ່ນ 1<br>No ບໍ່ແມ່ນ 0<br>Don't know / prefer not to say ບໍ່ຮູ້ / ບໍ່ຢາກກ່າວ 9
**3.11.**	In the earlier question ***2.3.1***, you mentioned that healthcare services had [***improved/not changed/worsened***] in the last three months. Do you think that the workshop activities were responsible for that?<br>ໃນຄຳຖາມ 2.3.1 ກ່ອນໜ້ານີ້, ທ່ານໄດ້ກ່າວເຖິງການບໍລິການຂອງສຸກສາລາ/ໂຮງໝໍນ້ອຍໄດ້ **[ມີການພັດທະນາຂຶ້ນ/ບໍ່ມີການປ່ຽນແປງ/ບໍ່ດີກ່ອນເກົ່າ]** ໃນໄລຍະເວລາ 3 ເດືອນທີ່ຜ່ານມາ. ທ່ານຄິດວ່າກິດຈະກຳຂອງກອງປະຊຸມເຫຼົ່ານີ້ໄດ້ມີຜົນກະທົບຫຍັງບໍ່ຕໍ່ຂໍ້ນັ້ນ?	Yes ແມ່ນ 1<br>No ບໍ່ແມ່ນ 0<br>Don't know / prefer not to say ບໍ່ຮູ້ / ບໍ່ຢາກກ່າວ 9
**Module IV: COVID impact, attitudes, testing and information seeking behaviour [*Rounds 1 and 2*]**<br>**ພາກທີ IV: ຜົນກະທົບຂອງໂຄວິດ, ທັດສະນະຄະຕິ, ການທົດສອບ ແລະ ພຶດຕິກຳໃນການຊອກຫາຂໍ້ມູນ [ຮອບທີ1 ແລະ 2]**		
**4.**	Thank you. Now I would like to ask you a few more questions about the COVID pandemic in particular.<br>ຂໍຂອບໃຈ. ຕໍ່ໄປພວກເຮົາຢາກຂໍຖາມຕື່ມ 2-3 ຄຳຖາມ ກ່ຽວກັບໂລກລະບາດຂອງພະຍາດໂຄວິດໂດຍສະເພາະ	
**4.1.**	Do you know anyone who has ever had COVID? (including yourself)<br>ທ່ານເຄີຍຮູ້ຈັກບຸກຄົນອື່ນທີ່ເຄີຍຕິດເຊື້ອພະຍາດໂຄວິດ ຫຼື ບໍ່?<br>(ລວມເຖິງຕົວທ່ານເອງ)	Yes ແມ່ນ 1<br>No ບໍ່ແມ່ນ 0<br>Don't know / prefer not to say ບໍ່ຮູ້ / ບໍ່ຢາກກ່າວ 9

**4.2.** From where do you receive information about COVID? [***mark all that apply***]<br>ໄດ້ຮັບຂໍ້ມູນກ່ຽວກັບໂຄວິດຈາກຊ່ອງທາງໃດ? **[ເລືອກໄດ້ຫຼາຍກວ່າ 1 ຂໍ້]**	Family members ສະມາຊິກໃນຄອບຄົວ 1<br>Other villagers ສະມາຊິກໃນກຸ່ມບ້ານ 2<br>Village leader / loudspeaker ຜູ້ນໍາຂັ້ນບ້ານ/ການປະກາດໂທລະໂຄ່ງ 3<br>Temple ວັດ 4<br>Church ໂບດ 5<br>Lao Front ແນວລາວສ້າງຊາດ 6<br>Lao Women's Union ສະຫະພັນແມ່ຍິງ 7<br>Lao Youth Union ສູນກາງຊາວໜຸ່ມ 8<br>Other authorities ແລະ ອໍານາດການປົກຄອງ ອື່ນໆ 9<br><br>Posters ໃບປິວ 10<br>Newspaper ໜັງສືພິມ 11<br>TV / radio ໂທລະພາບ/ວິທະຍຸ 12<br>Social media ສືສັງຄົມອອນລາຍ 13<br>Other online websites ເວັບໄຊອອນລາຍອື່ນໆ 14<br><br>Shop / vendor selling drugs ຮ້ານຄ້າ/ຮ້ານຂາຍຢາທົ່ວໄປ 20<br>Traditional healer ໝໍພື້ນເມືອງ/ໝໍຜີ 21<br>Pharmacist ແພດການຢາ 22<br>Private clinic/hospital ຄຣີນິກເອກະຊົນ/ໂຮງໝໍ 23<br>NGO clinic/service ຄຣີນິກຂອງອົງການຈັດຕັ້ງສາກົນ/ການບໍລິການ 24<br>Health centre ສຸກສາລາ/ໂຮງໝໍນ້ອຍ 25<br>Public hospital ໂຮງໝໍໃຫຍ່ (ໂຮງໝໍລັດ) 26<br>Village health volunteer ອາສາສະໝັກສາທາລະນະສຸກບ້ານ (ອສບ) 27<br>Unregistered/local/informal/retired doctor 28<br>ໝໍບໍ່ມີໃບຢັ້ງຢືນແພດ/ທ້ອງຖິ່ນ/ບໍ່ເປັນທາງການ/ໝໍບໍານານ<br>Traditional birth assistant ໝໍຕໍາແຍ 29<br>Others 99
**4.3.** Have you been vaccinated against COVID?<br>ທ່ານເຄີຍສັກຢາຕ້ານພະຍາດໂຄວິດບໍ່?	Yes ແມ່ນ 1<br>No ບໍ່ແມ່ນ 0<br>Don't know / prefer not to say ບໍ່ຮູ້ / ບໍ່ຢາກກ່າວ 9
**4.3.1.** [***if yes to 4.3***] How many vaccine doses did you receive? [ຖ້າແມ່ນ ຂໍ້4.3]ທ່ານໄດ້ຮັບວັກຊີນຈັກເຂັມແລ້ວ?	0 0<br>1 1<br>2 2<br>3 3<br>4 4<br>5 5<br>>5 6<br>Don't know / prefer not to say ບໍ່ຮູ້ / ບໍ່ຢາກກ່າວ 9
**4.3.2.** [***if no to 4.3***] Why were you not vaccinated? [ຖ້າບໍ່ແມ່ນ ໃນຂໍ້4.3] ເປັນຫຍັງທ່ານຈຶ່ງບໍ່ສັກຢາ?	Reason ເຫດຜົນ: ______________________
**4.3.3.** What would be the optimal number of COVID vaccine doses for you?<br>ຈໍານວນວັກຊີນ COVID ທີ່ດີທີ່ສຸດສໍາລັບເຈົ້າແມ່ນຈັກເຂັມ?	0 0<br>1 1<br>2 2<br>3 3<br>4 4<br>5 5<br>>5 6<br>Don't know / prefer not to say ບໍ່ຮູ້ / ບໍ່ຢາກກ່າວ 9
**4.4.** [DELETED]	
**4.4.1.** [DELETED]	
**4.4.2.** [DELETED]	
**4.5.** [DELETED]	
**4.6.** [DELETED]	
**4.7.** Are you worried to disclose your COVID-19 status with anyone if you get it in the future? ທ່ານກັງວົນທີ່ຈະເຜີຍແຜ່ສະຖານະຂອງຕົນເອງ ຫຼື ບໍ່ ຖ້າຫາກທ່ານເກີດຕິດເຊື້ອໄວຣັສ COVID-19?	Yes ແມ່ນ 1<br>No ບໍ່ແມ່ນ 0<br>Don't know / prefer not to say ບໍ່ຮູ້ / ບໍ່ຢາກກ່າວ 9

Module V: Maternal healthcare [*women in childbearing age*] (Rounds 1 & 2)

ພາກທີ 5: ການເບິ່ງແຍງສຸຂະພາບຂອງແມ່ [ແມ່ຍິງທີ່ຢູ່ໃນຊ່ວງເວລາການເກີດລູກ]

5. Thank you. Now I would like to ask you a few more questions that relate to maternal healthcare, but those are specific for women who are currently pregnant or recently gave birth.
ຂໍຂອບໃຈ. ຕໍ່ໄປພວກເຮົາຈະສອບຖາມຕື່ມ 2-3 ຄໍາຖາມທີ່ກ່ຽວຂ້ອງກັບ ການເບິ່ງແຍງສຸຂະພາບຂອງແມ່, ແຕ່ຄໍາຖາມເຫຼົ່ານັ້ນຈະເຈາະຈົງສະເພາະສໍາລັບແມ່ຍິງທີ່ເກີດລູກໃໝ່ກໍາລັງຖືພາ ຫຼື ເກີດລູກເມື່ອບໍ່ດົນມານີ້

**5.1.** [***If woman in childbearing age***] Did you deliver a baby in the past 12 months or are you currently pregnant? [***end module and go to Module VI if not currently or recently pregnant***]	Currently pregnant ກໍາລັງຖືພາ 1<br>Recently pregnant ຖືພາເມື່ອບໍ່ດົນມານີ້ 2

[ຖ້າຫາກແມ່ຍິງທີ່ຢູ່ໃນຊ່ວງເວລາໃນການເກີດລູກ] ທ່ານໄດ້ເກີດລູກເມື່ອ 12 ເດືອນ ທີ່ຜ່ານມາ,ຫຼື ຂະນະນີ້ໄດ້ກຳລັງຖືພາບໍ່? [ຜ່ານພາກທີ 5 ນີ້, ຖ້າຫາກບໍ່ໄດ້ຖືພາຢູ່ ຫຼື ຖືພາເມື່ອບໍ່ດົນມານີ້]	No ບໍ່ໄດ້ຖືພາ 0 **complete Module VI ຂ້າມໄປພາກທີ 6**<br>Don't know ບໍ່ຮູ້/ prefer not to say ບໍ່ຢາກກ່າວ 9
**5.2.** When did your most recent pregnancy start?<br>ການຖືພາຄັ້ງລ່າສຸດຂອງທ່ານເລີ່ມຕົ້ນຂຶ້ນເມື່ອໃດ?	Onset: ___ days / ______ weeks / _____ months / _____ years ago<br>ເລີ່ມມີອາການ/ອຸບັດຕິເຫດ: ______ ມື້ / ______ ອາທິດ / _____ ເດືອນ _____/ປີຜ່ານມາ<br>*[or]*<br>Date ____ m / _____ year ວັນ ____ ເດືອນ / _____ ປີ
5.2.a) [***For recently pregnant women***] When did you deliver your child?<br>**[ສຳລັບແມ່ຍິງທີ່ຫາກໍ່ຖືພາ]** ທ່ານເກີດລູກເມື່ອໃດ?	___ days / ______ weeks / _____ months / _____ years ago<br>______ ມື້ / ______ ອາທິດ / _____ ເດືອນ _____/ປີຜ່ານມາ<br>*[or]*<br>Date ____ m / _____ year ວັນ ____ ເດືອນ / _____ ປີ
**5.3.** [***For currently pregnant women***] In the last three months, did anyone advise you to seek specific kinds of care during your pregnancy? [ສຳລັບແມ່ຍິງທີ່ກຳລັງຖືພາ] ໃນຊ່ວງ 3ເດືອນ ທີ່ຜ່ານມາ, ເຄີຍມີຄົນໃຫ້ຄຳແນະນຳທ່ານຊອກຫາການຮັກສາແບບສະເພາະເຈາະຈົງບໍ່ ໃນຊ່ວງການຖືພາຂອງທ່ານ?	Yes ແມ່ນ 1<br>No ບໍ່ແມ່ນ 0<br>Don't know / prefer not to say ບໍ່ຮູ້ / ບໍ່ຢາກກ່າວ 9
**5.3.1.** [***if yes***] Who advised you? [***mark all that apply***]<br>[ຖ້າແມ່ນ] ຜູ້ໃດເປັນຄົນໃຫ້ຄຳແນະນຳທ່ານ?<br>[ສາມາດໝາຍໄດ້ຫຼາຍກວ່າໜຶ່ງຂໍ້]	Family member ສະມາຊິກໃນຄອບຄົວ 1<br>Friend / other villager ໝູ່/ຊາວບ້ານ 2<br>Traditional healer ໝໍພື້ນເມືອງ/ໝໍຜີ 3<br>Pharmacist ແພດການຢາ/ຄົນຂາຍຢາ 4<br>Village health volunteer ອາສາສະໝັກສາທາລະນະສຸກບ້ານ (ອສບ) 5<br>Auxiliary nurse ຜູ້ຊ່ວຍພະຍາບານ 6<br>Nurse or midwife ພະຍາບານ ຫຼື ຜະດຸງຄັນ 7<br>Other health centre staff ເຈົ້າໜ້າທີ່ສຸກສາລາ 8<br>Medical doctor (public hospital) ແພດ/ທ່ານໝໍ (ໂຮງໝໍລັດ) 9<br>Medical doctor (private hospital) ແພດ/ທ່ານໝໍ (ໂຮງໝໍເອກະຊົນ) 10<br>Unregistered/local/informal/retired doctor ໝໍບໍ່ມີໃບຢັ້ງຢືນແພດ/ທ້ອງຖິ່ນ/ບໍ່ເປັນທາງການ/ໝໍບຳນານ 11<br>Traditional birth assistant ໝໍຕຳແຍ 15<br><br>Village chief / deputy ນາຍບ້ານ/ຮອງນາຍບ້ານ 21<br>Lao Front ແນວລາວສ້າງຊາດ 22<br>Lao Women's Union ສະຫະພັນແມ່ຍິງ 23<br>Lao Youth Union ສູນກາງຊາວໜຸ່ມ 24<br>Other authorities ແລະ ອຳນາດການປົກຄອງ ອື່ນໆ 25<br>Other (specify) ອື່ນໆ (ລະບຸ): ________________________ 12<br>Don't know / prefer not to say ບໍ່ຮູ້ / ບໍ່ຢາກກ່າວ 99

5.4. I would like you to think back to the early stages of your pregnancy. Could you please walk me through the process from when you realised you were pregnant until the moment you delivered [recently pregnant] / until today [currently pregnant]? Take your time to remember, I would like to go through the full process with you. ຂ້າພະເຈົ້າຢາກໃຫ້ທ່ານຄິດຍ້ອນກັບໄປໃນຊ່ວຍຂັ້ນທຳອິດຂອງການຖືພາຂອງທ່ານ. ທ່ານຊ່ວຍພາເຮົາໄປຍັງຂະບວນການຕອນເມື່ອທ່ານຮູ້ແລ້ວວ່າຖືພາຈົນເຖິງເມື່ອທ່ານເກີດລູກ[ຫາກໍ່ເກີດລູກ]/ຈົນກະທັ່ງມື້ນີ້ [ກຳລັງຖືພາ]? ໃຊ້ເວລາໃນການຈື່, ຂ້າພະເຈົ້າຢາກຈະຜ່ານຊ່ວງເວລາຂອງການຖືພານີ້ໄປພ້ອມກັບທ່ານ.

	**5.4.1.** Source 1 ແຫຼ່ງ 1	Source n<br>ແຫຼ່ງ n

a) What kind of help or treatment did you get at this stage? [*if unsure, specify*] ການຊ່ວຍເຫຼືອ ຫຼື ການປິ່ນປົວປະເພດໃດທີ່ທ່ານໄດ້ຮັບໃນຂັ້ນນີ້? [ຖ້າບໍ່ແນ່ໃຈ, ຊ່ວຍລະບຸ]	Self-care (sleep, rest, medicine at home) ດູແລຕົວເອງ (ນອນ, ພັກຜ່ອນ, ກິນຢາຢູ່ບ້ານ) 1<br>Care from family and friends (full-time) ການເບິ່ງແຍງຈາກຄອບຄົວ ແລະ ໝູ່ເພື່ອນ (ເຕັມເວລາ) 2<br>Treated/cons. at shop / vendor selling drugs ໄດ້ຮັບການປິ່ນປົວຢູ່ຮ້ານຄ້າ/ຄົນຂາຍຢາ 3<br>Treated/consulted at a traditional healer ໄດ້ຮັບການປິ່ນປົວ/ປຶກສາຢູ່ໝໍພື້ນເມືອງ/ໝໍຜີ 4<br>Treated/cons. at a pharmacist ໄດ້ຮັບການປິ່ນປົວ/ປຶກສາກັບແພດຂາຍຢາ 5<br>Treated/cons. at priv. clinic/hospital ໄດ້ຮັບການປິ່ນປົວ/ປຶກສາທີ່ຄຣີນິກ/ໂຮງໝໍ 6<br>Treated/cons. at NGO clinic/service ໄດ້ຮັບການປິ່ນປົວ/ປຶກສາທີ່ຄຣີນິກບໍ່ສະແຫວງຫາຜົນກຳໄລ/ໂຮງໝໍ 7<br>Treated/cons. at health centre ໄດ້ຮັບການປິ່ນປົວ/ປຶກສາທີ່ສຸກສາລາ/ໂຮງໝໍນ້ອຍ 8<br>Treated/cons. at a public hospital ໄດ້ຮັບການປິ່ນປົວ/ປຶກສາທີ່ໂຮງໝໍເອກະຊົນ 9<br>Treated/cons. by a village health volunteer ໄດ້ຮັບການປິ່ນປົວ/ອາສາສະໝັກສາທາລະນະສຸກບ້ານ (ອສບ) 10<br>Treated/cons. by an unregistered/local/informal/retired doctor ໄດ້ຮັບການປິ່ນປົວ/ປຶກສາໂດຍໝໍທີ່ບໍ່ມີໃບຢັ້ງຢືນ/ທ້ອງຖິ່ນ/ບໍ່ເປັນທາງການ/ບຳນານ 11<br>Treated/consulted at a traditional birth assistant ໄດ້ຮັບການປິ່ນປົວ/ປຶກສາຢູ່ໝໍຕຳແຍ 15<br>Cross-border care/ປະເທດໃກ້ຄຽງ 97<br>Other (incl. internet; specify)ອື່ນໆ (ປະກອບທັງ ອິນເຕີເນັດ; ລະບຸ ___ 99	1<br>2<br>3<br>4<br>5<br>6<br>7<br>8<br>9<br>10<br>11<br>15<br>99
b) What was the purpose of getting help or treatment from this source? ຈຸດປະສົງຂອງການໄດ້ຮັບການຊ່ວຍເຫຼືອ ຫຼື ປິ່ນປົວຈາກແຫຼ່ງນັ້ນແມ່ນຫຍັງ?	Purpose ຈຸດປະສົງ: ________________________________	___
c) [*if not ignore/self-care*] How did the provider talk to you? [*mark all that apply*] [ຖ້າບໍ່ແມ່ນ, ຂ້າມ] ຜູ້ຊ່ວຍໄດ້ເວົ້າຈາໂອ້ລົມແບບໃດກັບທ່ານ? [ໝາຍໄດ້ຫຼາຍກວ່າໜຶ່ງຂໍ້]	Supportive / understanding / praising ສະໜັບສະໜູນ/ເຂົ້າໃຈ/ຍົກຍ້ອງ 1<br>Nice / kind / friendly / positive ດີ/ໃຈດີ/ເມດຕາ/ແງ່ດີ 2<br>Neutral / descriptive / unremarkable ປານກາງ/ພໍບັນລະຍາຍໄດ້/ບໍ່ປະທັບໃຈປານໃດ 3<br>Criticising / negative ວິຈານ/ແງ່ລົບ 4<br>Scolding / raising their voice / yelling ບ້ອຍດ່າ/ຂຶ້ນສຽງໃສ່/ຮ້ອງໃສ່ 5<br>Other (specify)ອື່ນໆ (ລະບຸ): ___________ 6<br><br>Not applicable ບໍ່ແມ່ນທັງໝົດ 8<br>Don't know / prefer not to say ບໍ່ຮູ້/ບໍ່ຢາກກ່າວ 9	1<br>2<br>3<br>4<br>5<br>6 __<br><br>8<br>9
d) [*if not ignore/self-care*] How well did you feel taken care of by the provider? [ຖ້າບໍ່ແມ່ນ, ຂ້າມ] ທ່ານຮູ້ສຶກດີສໍ່າໃດສໍາລັບການເບິ່ງແຍດູແລຈາກຜູ້ຊ່ວຍນັ້ນ?	Very well ດີຫຼາຍ 1<br>Well ດີ 2<br>So-so / indifferent / no opinion ປານກາງ/ບໍ່ແຕກຕ່າງ/ບໍ່ມີຄວາມຄິດເຫັນ 3<br>Not well ບໍ່ດີ 4<br>Unacceptably badly ບໍ່ສາມາດຮັບໄດ້ 5<br><br>Not relevant ບໍ່ກ່ຽວຂ້ອງ 8<br>Prefer not to say ບໍ່ຢາກກ່າວ 9	1<br>2<br>3<br>4<br>5<br><br>8<br>9

**5.4.1.1.** Has any healthcare provider supported you to develop a birth plan? If so, who? ມີຜູ້ສະໜອງການປິ່ນປົວຄົນໃດຊ່ວຍເຫຼືອທ່ານໃນການພັດທະນາແຜນການເກີດບໍ່? ຖ້າມີ, ແມ່ນໃຜ?	Family member ສະມາຊິກໃນຄອບຄົວ 1<br>Friend / other villager ໝູ່/ຊາວບ້ານ 2<br>Traditional healer ໝໍພື້ນເມືອງ/ໝໍຜີ 3<br>Pharmacist ແພດການຢາ/ຄົນຂາຍຢາ 4<br>Village health volunteer ອາສາສະໝັກສາທາລະນະສຸກບ້ານ (ອສບ) 5<br>Auxiliary nurse ຜູ້ຊ່ວຍພະຍາບານ 6<br>Nurse or midwife ພະຍາບານ ຫຼື ຜະດຸງຄັນ 7<br>Other health centre staff ເຈົ້າໜ້າທີ່ສຸກສາລາ 8<br>Medical doctor (public hospital) ແພດ/ທ່ານໝໍ (ໂຮງໝໍລັດ) 9<br>Medical doctor (private hospital) ແພດ/ທ່ານໝໍ (ໂຮງໝໍເອກະຊົນ) 10<br>Unregistered/local/informal/retired doctor ໝໍບໍ່ມີໃບຢັ້ງຢືນແພດ/ທ້ອງຖິ່ນ/ບໍ່ເປັນທາງການ/ໝໍບໍານານ 11<br>Traditional birth assistant ໝໍຕໍາແຍ 15<br><br>Village chief / deputy ນາຍບ້ານ/ຮອງນາຍບ້ານ 21<br>Lao Front ແນວລາວສ້າງຊາດ 22<br>Lao Women's Union ສະຫະພັນແມ່ຍິງ 23<br>Lao Youth Union ສູນກາງຊາວໜຸ່ມ 24<br>Other authorities ແລະ ອໍານາດການປົກຄອງ ອື່ນໆ 25<br>Other (specify) ອື່ນໆ (ລະບຸ): ______________________ 12<br><br>No one ບໍ່ມີໃຜ 98<br>Don't know / prefer not to say ບໍ່ຮູ້ / ບໍ່ຢາກກ່າວ 99
**5.5.** In total, do you remember how many ante-natal care visits you had at the health centre during your (current/recent) pregnancy? ໂດຍລວມແລ້ວ, ທ່ານໄປຝາກຄັນຈັກເທື່ອຢູ່ສຸກສາລາ/ໂຮງໝໍນ້ອຍລະຫວ່າງ (ປະຈຸບັນ/ລ່າສຸດ) ຂອງການຖືພາຂອງທ່ານ?	0 0<br>1 1<br>2 2<br>3 3<br>4 4<br>5 5<br>>5 6<br>Don't know / prefer not to say ບໍ່ຮູ້ / ບໍ່ຢາກກ່າວ 9
[***if 5.5 > 0***] [ຖ້າ***5.5 > 0***] As part of your antenatal care at health centres during this pregnancy, were any of the following procedures done at least once? ໃນສ່ວນຂອງການຝາກຄັນຂອງສຸກສາລາ/ໂຮງໝໍນ້ອຍໃນໄລຍະການຖືພານີ້, ມີການດໍາເນີນການໃນແດ່ໃນຄໍາຖາມຕໍ່ໄປນີ້ທີ່ເກີດຂຶ້ນຢ່າງໜ້ອຍໜຶ່ງຄັ້ງ	
**5.5.1.** Was your blood pressure measured? ທ່ານໄດ້ວັດແທກຄວາມດັນເລືອດບໍ່?	Yes ແມ່ນ 1<br>No ບໍ່ແມ່ນ 0<br>Don't know / prefer not to say ບໍ່ຮູ້ / ບໍ່ຢາກກ່າວ 9
5.5.1.b) Did the health centre staff check your baby's heartbeat? ພະນັກງານແພດໝໍຢູ່ສຸກສາລາໄດ້ກວດການເຕັ້ນຂອງຫົວໃຈຂອງລູກທ່ານ ຫຼືບໍ່?	Yes ແມ່ນ 1<br>No ບໍ່ແມ່ນ 0<br>Don't know / prefer not to say ບໍ່ຮູ້ / ບໍ່ຢາກກ່າວ 9
5.5.1.c) Did you receive an iron supplement? ທ່ານໄດ້ກິນຢາບໍາລຸງໄລຍະຖືພາບໍ່?	Yes ແມ່ນ 1<br>No ບໍ່ແມ່ນ 0<br>Don't know / prefer not to say ບໍ່ຮູ້ / ບໍ່ຢາກກ່າວ 9
**5.5.2.** [DELETED]	
**5.5.3.** [DELETED]	
**5.5.4.** [DELETED]	
**5.5.4.1.** [DELETED]	
5.5.b) Has anyone encouraged you to receive ante-natal care specifically from the health centre? ມີໃຜຊຸກຍູ້ໃຫ້ທ່ານໄປຝາກທ້ອງເປັນພິເສດທີ່ສຸກສາລາບໍ່?	Family member ສະມາຊິກໃນຄອບຄົວ 1<br>Friend / other villager ໝູ່/ຊາວບ້ານ 2<br>Traditional healer ໝໍພື້ນເມືອງ/ໝໍຜີ 3<br>Pharmacist ແພດການຢາ/ຄົນຂາຍຢາ 4<br>Village health volunteer ອາສາສະໝັກສາທາລະນະສຸກບ້ານ (ອສບ) 5<br>Auxiliary nurse ຜູ້ຊ່ວຍພະຍາບານ 6<br>Nurse or midwife ພະຍາບານ ຫຼື ຜະດຸງຄັນ 7<br>Other health centre staff ເຈົ້າໜ້າທີ່ສຸກສາລາ 8<br>Medical doctor (public hospital) ແພດ/ທ່ານໝໍ (ໂຮງໝໍລັດ) 9<br>Medical doctor (private hospital) ແພດ/ທ່ານໝໍ (ໂຮງໝໍເອກະຊົນ) 10<br>Unregistered/local/informal/retired doctor ໝໍບໍ່ມີໃບຢັ້ງຢືນແພດ/ທ້ອງຖິ່ນ/ບໍ່ເປັນທາງການ/ໝໍບໍານານ 11<br>Traditional birth assistant ໝໍຕໍາແຍ 15<br><br>Village chief / deputy ນາຍບ້ານ/ຮອງນາຍບ້ານ 21<br>Lao Front ແນວລາວສ້າງຊາດ 22<br>Lao Women's Union ສະຫະພັນແມ່ຍິງ 23<br>Lao Youth Union ສູນກາງຊາວໜຸ່ມ 24

	Other authorities ແລະ ອຳນາດການປົກຄອງ ອື່ນໆ 25<br>Other (specify) ອື່ນໆ (ລະບຸ): ________________________ 12<br><br>No one ບໍ່ມີໃຜ 98<br>Don't know / prefer not to say ບໍ່ຮູ້ / ບໍ່ຢາກກ່າວ 99
**5.6.** [***Rounds 1 & 2: Currently pregnant women***] Where do you plan to give birth? [ຄັ້ງທີ1 & 2: ແມ່ຍິງທີ່ກຳລັງຖືພາ] ທ່ານວາງແຜນທີ່ຈະເກີດລູກຢູ່ໃສ?<br>[***Recently pregnant women***] Where did you give birth? [ແມ່ຍິງທີ່ເກີດລູກເມື່ອບໍ່ດົນມານີ້] ທ່ານໄດ້ເກີດລູກຢູ່ສະຖານທີ່ໃດ?	Public hospital ໂຮງໝໍລັດ 1<br>Health centre ສຸກສາລາ/ໂຮງໝໍນ້ອຍ 2<br>Private clinic ຄລີນິກເອກະຊົນ 3<br>Own home ບ້ານຂອງຕົນເອງ 4<br>Other home ບ້ານອື່ນ 5<br>Cross-border care/ປະເທດໃກ້ຄຽງ 97<br>Other ອື່ນໆ(specify) ລະບຸ: ______________ 98<br>Don't know / prefer not to say ບໍ່ຮູ້ / ບໍ່ຢາກກ່າວ 99
**5.6.1.** If at home, who would you expect to be / was present to help? [***mark all that apply***] ຖ້າຢູ່ບ້ານຂອງຕົນເອງ, ທ່ານຄາດຫວັງໃຫ້ໃຜເປັນ/ຫຼືຕາງໜ້າໃນການທີ່ຈະຊ່ວຍທ່ານເກີດລູກ? [ທ່ານສາມາດເລືອກໄດ້ຫຼາຍກວ່າ 1 ຂໍ້]	Family member ສະມາຊິກໃນຄອບຄົວ 1<br>Friend / other villager ໝູ່/ຊາວບ້ານ 2<br>Traditional healer ໝໍພື້ນເມືອງ/ໝໍຜີ 3<br>Pharmacist ແພດການຢາ/ຄົນຂາຍຢາ 4<br>Health volunteer ແພດອາສາ 5<br>Auxiliary nurse ຜູ້ຊ່ວຍພະຍາບານ 6<br>Nurse or midwife ພະຍາບານ ຫຼື ຜະດຸງຄັນ 7<br>Other health centre staff ເຈົ້າໜ້າທີ່ສຸກສາລາ 8<br>Medical doctor (public hospital) ແພດ/ທ່ານໝໍ (ໂຮງໝໍລັດ) 9<br>Medical doctor (private hospital) ແພດ/ທ່ານໝໍ (ໂຮງໝໍເອກະຊົນ) 10<br>Unregistered/local/informal/retired doctor ໝໍບໍ່ມີໃບຢັ້ງຢືນແພດ/ທ້ອງຖິ່ນ/ບໍ່ເປັນທາງການ/ໝໍບໍານານ 11<br>Traditional birth assistant ໝໍຕຳແຍ 15<br>Other (specify) ອື່ນໆ (ລະບຸ): ________________________ 12<br>Don't know / prefer not to say ບໍ່ຮູ້ / ບໍ່ຢາກກ່າວ 99
**5.6.2.** What is/was the main reason for your choice? ສິ່ງໃດເປັນເຫດຜົນຫຼັກໃນການເລືອກຂອງທ່ານ?	Reason ເຫດຜົນ: _______________________
**5.6.3.** Has anyone encouraged you to give birth at that place? ມີໃຜແນະນຳໃຫ້ທ່ານເກີດລູກຢູ່ສະຖານທີ່ນັ້ນບໍ່?	Family member ສະມາຊິກໃນຄອບຄົວ 1<br>Friend / other villager ໝູ່/ຊາວບ້ານ 2<br>Traditional healer ໝໍພື້ນເມືອງ/ໝໍຜີ 3<br>Pharmacist ແພດການຢາ/ຄົນຂາຍຢາ 4<br>Village health volunteer ອາສາສະໝັກສາທາລະນະສຸກບ້ານ (ອສບ) 5<br>Auxiliary nurse ຜູ້ຊ່ວຍພະຍາບານ 6<br>Nurse or midwife ພະຍາບານ ຫຼື ຜະດຸງຄັນ 7<br>Other health centre staff ເຈົ້າໜ້າທີ່ສຸກສາລາ 8<br>Medical doctor (public hospital) ແພດ/ທ່ານໝໍ (ໂຮງໝໍລັດ) 9<br>Medical doctor (private hospital) ແພດ/ທ່ານໝໍ (ໂຮງໝໍເອກະຊົນ) 10<br>Unregistered/local/informal/retired doctor ໝໍບໍ່ມີໃບຢັ້ງຢືນແພດ/ທ້ອງຖິ່ນ/ບໍ່ເປັນທາງການ/ໝໍບໍານານ 11<br>Traditional birth assistant ໝໍຕຳແຍ 15<br><br>Village chief / deputy ນາຍບ້ານ/ຮອງນາຍບ້ານ 21<br>Lao Front ແນວລາວສ້າງຊາດ 22<br>Lao Women's Union ສະຫະພັນແມ່ຍິງ 23<br>Lao Youth Union ສູນກາງຊາວໜຸ່ມ 24<br>Other authorities ແລະ ອຳນາດການປົກຄອງ ອື່ນໆ 25<br>Other (specify) ອື່ນໆ (ລະບຸ): ________________________ 12<br><br>No one ບໍ່ມີໃຜ 98<br>Don't know / prefer not to say ບໍ່ຮູ້ / ບໍ່ຢາກກ່າວ 99

**5.6.4.** [*if 5.6 did not include "health centre"*] Has anyone encouraged you to give birth <u>specifically at the health centre</u>?<br>**[ຖ້າ 5.6 ບໍ່ໄດ້ມີ "ສຸກສາລາ"]**<br>ມີໃຜຊຸກຍູ້ໃຫ້ທ່ານເກີດລູກ<u>ຢູ່ສຸກສາລາໂດຍສະເພາະບໍ່?</u>	Family member ສະມາຊິກໃນຄອບຄົວ 1<br>Friend / other villager ໝູ່/ຊາວບ້ານ 2<br>Traditional healer ໝໍພື້ນເມືອງ/ໝໍຜີ 3<br>Pharmacist ແພດການຢາ/ຄົນຂາຍຢາ 4<br>Village health volunteer ອາສາສະໝັກສາທາລະນະສຸກບ້ານ (ອສບ) 5<br>Auxiliary nurse ຜູ້ຊ່ວຍພະຍາບານ 6<br>Nurse or midwife ພະຍາບານ ຫຼື ຜະດຸງຄັນ 7<br>Other health centre staff ເຈົ້າໜ້າທີ່ສຸກສາລາ 8<br>Medical doctor (public hospital) ແພດ/ທ່ານໝໍ (ໂຮງໝໍລັດ) 9<br>Medical doctor (private hospital) ແພດ/ທ່ານໝໍ (ໂຮງໝໍເອກະຊົນ) 10<br>Unregistered/local/informal/retired doctor ໝໍບໍ່ມີໃບຢັ້ງຢືນແພດ/ທ້ອງຖິ່ນ/ບໍ່ເປັນທາງການ/ໝໍບໍານານ 11<br>Traditional birth assistant ໝໍຕໍາແຍ 15<br><br>Village chief / deputy ນາຍບ້ານ/ຮອງນາຍບ້ານ 21<br>Lao Front ແນວລາວສ້າງຊາດ 22<br>Lao Women's Union ສະຫະພັນແມ່ຍິງ 23<br>Lao Youth Union ສູນກາງຊາວໜຸ່ມ 24<br>Other authorities ແລະ ອໍານາດການປົກຄອງ ອື່ນໆ 25<br>Other (specify) ອື່ນໆ (ລະບຸ): ________________________ 12<br><br>No one ບໍ່ມີໃຜ 98<br>Don't know / prefer not to say ບໍ່ຮູ້ / ບໍ່ຢາກກ່າວ 99
**5.6.5.** [*for recently pregnant women*] Can you describe the experience of giving birth at this place? How was it going into labour, did you feel safe?<br>[ແມ່ຍິງທີ່ເກີດລູກເມື່ອບໍ່ດົນມານີ້] ຊ່ວຍໃຫ້ທ່ານອະທິບາຍປະສົບການໃນການເກີດຂຶ້ນຢູ່ສະຖານທີ່ນີ້ໄດ້ບໍ່? ຕອນກໍາລັງເກີດລູກເປັນແນວໃດ? ທ່ານຮູ້ສຶກປອດໄພບໍ່?	Explanation ອະທິບາຍ: ______________________________
**5.6.6.** [*for recently pregnant women*] I would like to ask a few more questions about your experience and care from the doctors, nurses or other helpers at the place where you delivered your baby.<br>[ສໍາລັບແມ່ຍິງທີ່ກໍາເກີດລູກ] ຂ້າພະເຈົ້າຢາກຖາມທ່ານຄໍາຖາມຈໍານວນໜຶ່ງກ່ຽວກັບປະສົບການຂອງທ່ານ ແລະ ການເບິ່ງແຍງຈາກທ່ານໝໍ, ແພດ ຫຼື ຜູ້ສະໜອງການປິ່ນປົວອື່ນໆຢູ່ທີ່ບ່ອນທ່ານໃຫ້ກໍາເນີດລູກຂອງທ່ານ.	
**5.6.6.1.** Did they treat you in a friendly manner? ["they' is the doctors, nurses or other helpers at the place of delivery]<br>ພວກເຂົາໄດ້ປະຕິບັດກັບທ່ານດີຫຼືບໍ່? ["ພວກເຂົາ" ແມ່ນທ່ານໝໍ, ແພດ, ຫຼື ຜູ້ສະໜອງການປິ່ນປົວອື່ນໆທີ່ບ່ອນໃຫ້ປະສູດ ຫຼືໃຫ້ກໍາເນີດລູກ]	Yes ແມ່ນ 1<br>No ບໍ່ແມ່ນ 0<br>Don't know / prefer not to say ບໍ່ຮູ້ / ບໍ່ຢາກກ່າວ 9
**5.6.6.2.** During examinations (e.g. in the labor room), were you covered up with a cloth or blanket or screened with a curtain so that you did not feel exposed?<br>ໃນລະຫວ່າງການໃຫ້ກໍາເນີດລູກ (ເຊັ່ນວ່າ ທີ່ຫ້ອງປະສູດ), ທ່ານໝໍໄດ້ຄຸມຜ້າຫົ່ມ ຫຼື ເສື່ອຜ້າ ຫຼື ບິດບັງບ່ອນທີ່ບໍ່ໄດ້ຖືກເປີດເຜີຍບໍ່?	Yes ແມ່ນ 1<br>No ບໍ່ແມ່ນ 0<br>Don't know / prefer not to say ບໍ່ຮູ້ / ບໍ່ຢາກກ່າວ 9
**5.6.6.3.** Did you feel like they involved you in decisions about your care?<br>ທ່ານຮູ້ສຶກວ່າພວກເຂົາໄດ້ໃຫ້ທ່ານມີສ່ວນຮ່ວມໃນການຕັດສິນໃຈໃນການປິ່ນປົວຂອງທ່ານຫຼືບໍ່?	Yes ແມ່ນ 1<br>No ບໍ່ແມ່ນ 0<br>Don't know / prefer not to say ບໍ່ຮູ້ / ບໍ່ຢາກກ່າວ 9
**5.6.6.4.** Did they explain to you why they were carrying out examinations or procedures?<br>ພວກເຂົາໄດ້ອະທິບາຍໃຫ້ທ່ານບໍ່ວ່າເປັນຫຍັງເຂົາຈຶ່ງທໍາການກວດ ຫຼື ດໍາເນີນການກວດ?	Yes ແມ່ນ 1<br>No ບໍ່ແມ່ນ 0<br>Don't know / prefer not to say ບໍ່ຮູ້ / ບໍ່ຢາກກ່າວ 9
**5.6.6.5.** Did they explain to you why they were giving you any medicine?<br>ພວກເຂົາໄດ້ອະທິບາຍໃຫ້ທ່ານບໍ່ວ່າເປັນຫຍັງເຂົາຈຶ່ງເອົາຢາໃຫ້ທ່ານ?	Yes ແມ່ນ 1<br>No ບໍ່ແມ່ນ 0<br>Don't know / prefer not to say ບໍ່ຮູ້ / ບໍ່ຢາກກ່າວ 9
**5.6.6.6.** Did you feel you could ask them any questions you had?<br>ທ່ານຮູ້ສຶກບໍ່ວ່າທ່ານສາມາດຖາມຄໍາຖາມໃດໆກໍໄດ້ທີ່ທ່ານມີ?	Yes ແມ່ນ 1<br>No ບໍ່ແມ່ນ 0<br>Don't know / prefer not to say ບໍ່ຮູ້ / ບໍ່ຢາກກ່າວ 9

**5.6.6.7.** When you needed help, did you feel they paid attention?<br>ເມື່ອທ່ານຕ້ອງການຄວາມຊ່ວຍເຫຼືອ, ທ່ານຮູ້ສຶກວ່າພວກເຂົາໃສ່ໃຈ ຫຼື ໃຫ້ຄວາມສົນໃຈບໍ່?	Yes ແມ່ນ 1<br>No ບໍ່ແມ່ນ 0<br>Don't know / prefer not to say ບໍ່ຮູ້ / ບໍ່ຢາກກ່າວ 9
**5.6.6.8.** Did you feel they took the best care of you?<br>ທ່ານຮູ້ສຶກວ່າພວກເຂົາໄດ້ດູແລ ຫຼື ປິ່ນປົວທ່ານດີທີ່ສຸດຫຼືບໍ່?	Yes ແມ່ນ 1<br>No ບໍ່ແມ່ນ 0<br>Don't know / prefer not to say ບໍ່ຮູ້ / ບໍ່ຢາກກ່າວ 9
**5.7.** [***Recently pregnant women***] I now would like to ask you some questions specifically to the most recent birth. **[ສໍາລັບແມ່ຍິງທີ່ຫາກໍ່ຖືພາ]** ນະຕອນນີ້ຂ້າພະເຈົ້າຢາກຈະຖາມທ່ານກ່ຽວກັບການໃຫ້ກໍາເກີດຄັ້ງລ່າສຸດເປັນພິເສດ	
**5.7.1.** [DELETED]	
**5.7.2.** [DELETED]	
**5.7.3.** [DELETED]	
**5.7.4.** [DELETED]	
5.7.b) Did your child receive a birth registry?<br>ລູກຂອງທ່ານໄດ້ຈົດທະບຽນການເກີດບໍ່?<br>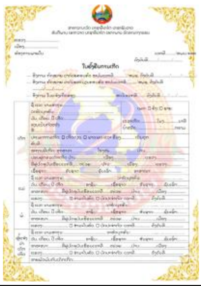	Yes ແມ່ນ 1<br>No ບໍ່ແມ່ນ 0<br>Don't know / prefer not to say ບໍ່ຮູ້ / ບໍ່ຢາກກ່າວ 9<br><br>[enumerators can take photograph of birth registry to avoid confusion]
5.7.5.a) Immediately after the birth, was the baby put directly on the bare skin of your chest?<br>ທັນທີຫຼັງຈາກເກີດລູກ, ລູກຂອງທ່ານໄດ້ຢູ່ໃນອ້ອມກອດຂອງທ່ານເລີຍບໍ່?	Yes ແມ່ນ 1<br>No ບໍ່ແມ່ນ 0<br>Don't know / prefer not to say ບໍ່ຮູ້ / ບໍ່ຢາກກ່າວ 9
5.7.5.b) How long after birth did you first put your baby to the breast? [If less than 1 hour, record '00' hours]<br>ດົນປານໃດທີ່ທ່ານໄດ້ໃຫ້ນົມລູກເປັນຄັ້ງທໍາອິດຫຼັງຈາກເກີດລູກ? [ຖ້າໜ້ອຍກວ່າ 1 ຊົ່ວໂມງ, ບັນທຶກ '00' ຊົ່ວໂມງ]	Specify time (hours or days) ລະບຸເວລາ (ຊົ່ວໂມງ ຫຼືມື້) 1<br>____ hours / ___ days<br>____ ຊົ່ວໂມງ / ______ ມື້<br><br>Did not breastfeed ບໍ່ໄດ້ໃຫ້ນົມລູກ 98<br>Don't know / prefer not to say ບໍ່ຮູ້ / ບໍ່ຢາກກ່າວ 99
5.7.5.c) Did the baby receive a Hepatitis B vaccination – that is an injection on the outside of the thigh to prevent Hepatitis B disease – within the first 24 hours after birth?<br>ລູກຂອງທ່ານໄດ້ສັກວັກຊີນພະຍາດຕັບອັກເສບບີ - ທີ່ສັກຢູ່ກົກຂາເພື່ອກັນພະຍາດຕັບອັກເສບບີ - ພາຍໃນ 24 ຊົ່ວໂມງທໍາອິດຫຼັງຈາກເກີດລູກຫຼືບໍ່?	Yes ແມ່ນ 1<br>No ບໍ່ແມ່ນ 0<br>Don't know / prefer not to say ບໍ່ຮູ້ / ບໍ່ຢາກກ່າວ 9
**5.7.5.** [***if yes to any of 5.7.5.a-c***] Where did these activities take place? [***mark all that apply***] [ຖ້າແມ່ນໃນຂໍ້ 5.7.5.a-c] ກິດຈະກໍາຕ່າງໆເຫຼົ່ານີ້ເກີດຂຶ້ນຢູ່ໃສ? [ສາມາດໝາຍໄດ້ຫຼາຍກວ່າໜຶ່ງຂໍ້]	Public hospital ໂຮງໝໍລັດ 1<br>Health centre ສຸກສາລາ/ໂຮງໝໍນ້ອຍ 2<br>Private clinic ຄລີນິກເອກະຊົນ 3<br>Own home ບ້ານຂອງຕົນເອງ 4<br>Other home ບ້ານຄົນອື່ນ 5<br>Other (specify): ອື່ນໆ (ລະບຸ)_______________ 8<br>Cross-border care/ປະເທດໃກ້ຄຽງ 97<br>Don't know / prefer not to say ບໍ່ຮູ້/ບໍ່ຢາກກ່າວ 9
**5.7.6.** [***if yes to any of 5.7.5.a-c***] Who performed those checks? [***mark all that apply***]<br>[ຖ້າແມ່ນໃນຂໍ້ 5.7.5.a-c] ໃຜເປັນຜູ້ກວດໃຫ້? [ສາມາດໝາຍໄດ້ຫຼາຍກວ່າໜຶ່ງຂໍ້]	Family member ສະມາຊິກໃນຄອບຄົວ 1<br>Friend / other villager ໝູ່/ຊາວບ້ານ 2<br>Traditional healer ໝໍພື້ນເມືອງ/ໝໍຜີ 3<br>Pharmacist ແພດການຢາ/ຄົນຂາຍຢາ 4<br>Village health volunteer ອາສາສະໝັກສາທາລະນະສຸກບ້ານ (ອສບ) 5<br>Auxiliary nurse ຜູ້ຊ່ວຍພະຍາບານ 6<br>Nurse or midwife ພະຍາບານ ຫຼື ຜະດຸງຄັນ 7<br>Other health centre staff ເຈົ້າໜ້າທີ່ສຸກສາລາ 8<br>Medical doctor (public hospital) ແພດ/ທ່ານໝໍ (ໂຮງໝໍລັດ) 9<br>Medical doctor (private hospital) ແພດ/ທ່ານໝໍ (ໂຮງໝໍເອກະຊົນ) 10<br>Unregistered/local/informal/retired doctor ໝໍບໍ່ມີໃບຢັ້ງຢືນແພດ/ທ້ອງຖິ່ນ/ບໍ່ເປັນທາງການ/ໝໍບໍານານ 11<br>Traditional birth assistant ໝໍຕໍາແຍ 15<br>Other (specify) ອື່ນໆ (ລະບຸ): _________________________ 12<br>Don't know / prefer not to say ບໍ່ຮູ້ / ບໍ່ຢາກກ່າວ 99

**5.8.** [***Recently pregnant women***] Did you experience any post-delivery complications? [ແມ່ມານທີ່ຫາກໍເກີດລູກ] ທ່ານໄດ້ປະສົບກັບພະຍາດແຊກຊ້ອນໃດໆບໍ່?	Yes ແມ່ນ 1<br>No ບໍ່ແມ່ນ 0<br>Don't know / prefer not to say ບໍ່ຮູ້ / ບໍ່ຢາກກ່າວ 9	
**5.8.1.** [***if yes***] Can you please tell me what you experienced? [***mark all that apply***] [ຖ້າແມ່ນ] ທ່ານສາມາດບອກໄດ້ບໍ່ວ່າທ່ານເຄີຍພົບມາແບບໃດ? [ສາມາດເລືອກໄດ້ຫຼາຍກວ່າ 1ຂໍ້]	High fever ໄຂ້ສູງ 1<br>Lower abdominal pain ເຈັບທ້ອງນ້ອຍ 2<br>Foul smelling vaginal discharge ລົງຂາວມີກິ່ນເໝັນ 3<br>Excessive bleeding ເລືອດອອກຫຼາຍເກີນໄປ 4<br>Convulsion ມີອາການຊັກ 5<br>Severe headache ເຈັບຫົວຮຸນແຮງ 6<br>Otherອື່ນໆ (specify ລະບຸ: ____________________) 7<br>Don't know ບໍ່ຮູ້/ prefer not to say ບໍ່ຢາກກ່າວ 9	
[***if any; if multiple episodes, focus on for the respondent most significant episode***] Can you please explain the stages of the treatment? I will ask you step-by-step what you did, starting from the moment you first experienced a discomfort [ຖ້າມີ, ຖ້າຫຼາຍຕອນ, ແໜ້ນໃສ່ຕອນທີ່ສຳຄັນທີ່ສຸດຂອງຜູ້ຕອບ] ທ່ານຊ່ວຍອະທິບາຍຂັ້ນຕອນຂອງການປິ່ນປົວໄດ້ບໍ່? ຂ້າພະເຈົ້າຈະຖາມເທື່ອລະຂັ້ນໃນສິ່ງທີ່ທ່ານເຮັດ, ເລີ່ມຈາກຊ່ວງເວລາທີ່ທ່ານປະສົບກັບຄວາມບໍ່ສະບາຍໃຈ	**5.8.2.** Step 1 (detection) ຂັ້ນຕອນ 1 ການກວດສອບ	Step n
a) What kind of help or treatment did you get at this stage? [***if unsure, specify***] ທ່ານໄດ້ຮັບການຊ່ວຍເຫຼືອ ຫຼື ປິ່ນປົວຫຍັງໃນຂັ້ນນີ້?	Self-care (sleep, rest, medicine at home) ດູແລຕົວເອງ (ນອນ, ພັກຜ່ອນ, ກິນຢາຢູ່ບ້ານ) 1<br>Care from family and friends (full-time) ການເບິ່ງແຍງຈາກຄອບຄົວ ແລະ ໝູ່ເພື່ອນ (ເຕັມເວລາ) 2<br>Treated/cons. at shop / vendor selling drugs ໄດ້ຮັບການປິ່ນປົວຢູ່ຮ້ານຄ້າ/ຄົນຂາຍຢາ 3<br>Treated/consulted at a traditional healer ໄດ້ຮັບການປິ່ນປົວ/ປຶກສາຢູ່ໝໍພື້ນເມືອງ/ໝໍຜີ 4<br>Treated/cons. at a pharmacist ໄດ້ຮັບການປິ່ນປົວ/ປຶກສາກັບແພດຂາຍຢາ 5<br>Treated/cons. at priv. clinic/hospital ໄດ້ຮັບການປິ່ນປົວ/ປຶກສາທີ່ຄລີນິກ/ໂຮງໝໍ 6<br>Treated/cons. at NGO clinic/service ໄດ້ຮັບການປິ່ນປົວ/ປຶກສາທີ່ຄລີນິກບໍ່ສະແຫວງຫາຜົນກຳໄລ/ໂຮງໝໍ 7<br>Treated/cons. at health centre ໄດ້ຮັບການປິ່ນປົວ/ປຶກສາທີ່ສຸກສາລາ/ໂຮງໝໍນ້ອຍ 8<br>Treated/cons. at a public hospital ໄດ້ຮັບການປິ່ນປົວ/ປຶກສາທີ່ໂຮງໝໍເອກະຊົນ 9<br>Treated/cons. by a village health volunteer ໄດ້ຮັບການປິ່ນປົວ/ ອາສາສະໝັກສາທາລະນະສຸກບ້ານ (ອສບ) 10<br>Treated/cons. by an unregistered/local/informal/retired doctor ໄດ້ຮັບການປິ່ນປົວ/ປຶກສາໂດຍໝໍທີ່ບໍ່ມີໃບຢັ້ງຢືນ/ທ້ອງຖິ່ນ/ບໍ່ເປັນທາງການ/ບໍານານ 11<br>Treated/consulted at a traditional birth assistant ໄດ້ຮັບການປິ່ນປົວ/ປຶກສາຢູ່ໝໍຕຳແຍ 15<br>Cross-border care/ປະເທດໃກ້ຄຽງ 97<br>Other (incl. internet; specify)ອື່ນໆ (ປະກອບທັງ ອິນເຕີເນັດ; ລະບຸ _____ 99	1<br>2<br>3<br>4<br>5<br>6<br>7<br>8<br>9<br>10<br>11<br>15<br>99
b) [***if not ignore/self-care***] How did the provider talk to you? [***mark all that apply***] [ຖ້າບໍ່ແມ່ນ ບໍ່ສົນໃຈ/ເບິ່ງແຍງຕົນເອງ] ຜູ້ສະໜອງການປິ່ນປົວໄດ້ໂອ້ລົມກັບທ່ານແບບໃດ?	Supportive / understanding / praising ສະໜັບສະໜູນ/ເຂົ້າໃຈ/ຍົກຍ້ອງ 1<br>Nice / kind / friendly / positive ດີ/ໃຈດີ/ເມດຕາ/ແງ່ດີ 2<br>Neutral / descriptive / unremarkable ປານກາງ/ພັນລະນາໄດ້/ບໍ່ປະທັບໃຈປານໃດ 3<br>Criticising / negative ວິຈານ/ແງ່ລົບ 4<br>Scolding / raising their voice / yelling ບ້ອຍດ່າ/ຂຶ້ນສຽງໃສ່/ຮ້ອງໃສ່ 5<br>Other (specify)ອື່ນໆ (ລະບຸ): ___________ 6<br><br>Not applicable ບໍ່ແມ່ນທັງໝົດ 8<br>Don't know / prefer not to say ບໍ່ຮູ້/ບໍ່ຢາກກ່າວ 9	1<br>2<br>3<br>4<br>5<br>6 __<br><br>8<br>9
c) [***if not ignore/self-care***] How well did you feel taken care of by the provider? [ຖ້າບໍ່ແມ່ນ ບໍ່ສົນໃຈ/ເບິ່ງແຍງຕົນເອງ] ທ່ານຮູ້ສຶກວ່າໄດ້ຮັບການຮັກສາຈາກຜູ້ສະໜອງການປິ່ນປົວໄດ້ດີປານໃດ?	Very well ດີຫຼາຍ 1<br>Well ດີ 2<br>So-so / indifferent / no opinion ປານກາງ/ບໍ່ແຕກຕ່າງ/ບໍ່ມີຄວາມຄິດເຫັນ 3<br>Not well ບໍ່ດີ 4<br>Unacceptably badly ບໍ່ສາມາດຮັບໄດ້ 5<br>Not relevant ບໍ່ກ່ຽວຂ້ອງ 8<br>Prefer not to say ບໍ່ຢາກກ່າວ 9	1<br>2<br>3<br>4<br>5<br><br>8<br>9
**5.8.3.** Have you now recovered from the complications? ຕອນນີ້ທ່ານຫາຍດີຈາກອາການຂ້າງຄຽງແລ້ວ ຫຼື ບໍ່?	Yes ແມ່ນ 1<br>No ບໍ່ແມ່ນ 0<br>Don't know / prefer not to say ບໍ່ຮູ້ / ບໍ່ຢາກກ່າວ 9	
**5.9.** [***Recently pregnant women***] Are you exclusively breastfeeding? **[ແມ່ຍິງທີ່ຫາກໍຖືພາ]** ທ່ານໄດ້ໃຫ້ນົມລູກພຽງແຕ່ຢ່າງດຽວບໍ່?	Yes ແມ່ນ 1<br>No ບໍ່ແມ່ນ 0<br>Don't know / prefer not to say ບໍ່ຮູ້ / ບໍ່ຢາກກ່າວ 9	

**5.9.1.** [*if no to 5.9*] What is the reason? **[ຖ້າບໍ່ ໃນ 5.9]** ເຫດຜົນແມ່ນຫຍັງ?	Explanation ອະທິບາຍ: ______________________________
**5.9.2.** Did someone help you to raise the baby only with breast milk? [if yes, indicate who]<br>ມີຄົນຊ່ວຍເຫຼືອທ່ານລ້ຽງລູກ ດ້ວຍນ້ຳນົມຈາກແມ່ແຕ່ຢ່າງດຽວບໍ່? [ຖ້າແມ່ນ, ລະບຸວ່າແມ່ນໃຜ]	Family member ສະມາຊິກໃນຄອບຄົວ 1<br>Friend / other villager ໝູ່/ຊາວບ້ານ 2<br>Traditional healer ໝໍພື້ນເມືອງ/ໝໍຜີ 3<br>Pharmacist ແພດການຢາ/ຄົນຂາຍຢາ 4<br>Village health volunteer ອາສາສະໝັກສາທາລະນະສຸກບ້ານ (ອສບ) 5<br>Auxiliary nurse ຜູ້ຊ່ວຍພະຍາບານ 6<br>Nurse or midwife ພະຍາບານ ຫຼື ພະດຸງຄັນ 7<br>Other health centre staff ເຈົ້າໜ້າທີ່ສຸກສາລາ 8<br>Medical doctor (public hospital) ແພດ/ທ່ານໝໍ (ໂຮງໝໍລັດ) 9<br>Medical doctor (private hospital) ແພດ/ທ່ານໝໍ (ໂຮງໝໍເອກະຊົນ) 10<br>Unregistered/local/informal/retired doctor ໝໍບໍ່ມີໃບຢັ້ງຢືນແພດ/ທ້ອງຖິ່ນ/ບໍ່ເປັນທາງການ/ໝໍບຳນານ 11<br>Traditional birth assistant ໝໍຕຳແຍ 15<br><br>Village chief / deputy ນາຍບ້ານ/ຮອງນາຍບ້ານ 21<br>Lao Front ແນວລາວສ້າງຊາດ 22<br>Lao Women's Union ສະຫະພັນແມ່ຍິງ 23<br>Lao Youth Union ສູນກາງຊາວໜຸ່ມ 24<br>Other authorities ແລະ ອຳນາດການປົກຄອງ ອື່ນໆ 25<br>Other (specify) ອື່ນໆ (ລະບຸ): ________________________ 12<br><br>No one ບໍ່ມີໃຜ 98<br>Don't know / prefer not to say ບໍ່ຮູ້ / ບໍ່ຢາກກ່າວ 99

Module VI: Healthcare Seeking ພາກທີ VI: ການຊອກຫາການເບິ່ງແຍງສຸຂະພາບ

Thank you for this. Now we come to the last part, where I will ask you some questions about health and health providers around here. ຂອບໃຈສຳລັບການຕອບຄຳຖາມ. ດຽວນີ້ເຮົາໄດ້ມາຮອດພາກສ່ວນສຸດທ້າຍແລ້ວ, ທີ່ຂ້າພະເຈົ້າຈະຖາມຄຳຖາມຈຳນວນໜຶ່ງກ່ຽວກັບສຸຂະພາບ ແລະ ຜູ້ສະໜອງ (ແພດໝໍ) ທີ່ຢູ່ນີ້.

**6.** [***if not pregnant or recently pregnant woman***] Did you or a child in your household (<16 years) under your supervision have an acute illness (not a chronic, long-term condition that comes again and again) or an accident in the last 3 months? If yes, I will ask you about these illnesses one-by-one. ໃນໄລຍະສາມເດືອນທີ່ຜ່ານມາ ທ່ານ ຫຼື ເດັກນ້ອຍໃນຄອບຄົວຂອງທ່ານ ມີອາການປ່ວຍ (ບໍ່ລວມກັບພະຍາດຊຳເຮື້ອ ຫຼື ພະຍາດທີ່ເປັນແລ້ວເປັນຊ້ຳໆ) ຫຼື ອຸບັດຕິເຫດ ຫຼືບໍ່? ຖ້າມີ, ເຮົາຂໍອະນຸຍາດເອົາລາຍລະອຽດຂອງແຕ່ລະອັນ ເລີ່ມຕົ້ງແຕ່ການປ່ວຍເທື່ອທຳອິດ	No ບໍ່ແມ່ນ 0 → [*go to end of questionnaire*] ຂ້າມໄປພາກສ່ວນສຸດທ້າຍຂອງແບບສອບຖາມ<br>Yes ແມ່ນ 1
[***if yes***:] [***ຖ້າຄຳຕອບຄື ແມ່ນ:***]<br>**6.1.** [Confirm if this episode is for respondent or child]<br>ຢັ້ງຢືນວ່າການເຈັບເປັນນີ້ເກີດຂຶ້ນກັບຜູ້ຖືກສຳພາດເອງ ຫຼື ເກີດຂຶ້ນກັບເດັກນ້ອຍພາຍໃຕ້ການດູແລເບິ່ງແຍງຂອງລາວ	Respondent ຕົນເອງ 1 → [***Q 6.1***]<br>Child ເດັກນ້ອຍ 2
**6.1.1.** [*if child*] How old is the child? ອາຍຸ	Age in years: _______ ອາຍຸ [ປີ]: _______
**6.1.2.** [*if child*] Is the child female or male ເພດ	Female ຍິງ 1<br>Male ຊາຍ 0
**6.2.** Can you please describe the symptoms or problem in your own words? ກະລຸນາອະທິບາຍອາການ ຫຼື ບັນຫາສຸຂະພາບຂອງເດັກໃນແບບທີ່ທ່ານເຂົ້າໃຈ	Description of condition: _________________<br>ອະທິບາຍອາການ: ______________________
**6.3.** When did [you / the child] experience the accident/discomfort (for the first time) ເລີ່ມມີອາການ ຫຼື ໄດ້ຮັບອຸບັດຕິເຫດນັ້ນໆ ໃນຕອນໃດ	Onset: ___ days / ______ weeks / _____ months ago<br>ເລີ່ມມີອາການ/ອຸບັດຕິເຫດ: ______ ມື້ / ______ ອາທິດ / _____ ເດືອນຜ່ານມາ
**6.4.** Would you describe the illness/accident as "mild," "moderate," or "severe"?<br>ທ່ານຄິດວ່າອາການເຈັບເປັນຫຼືອຸບັດຕິເຫດຄັ້ງນັ້ນຢູ່ຂັ້ນ "ເບົາ", "ປານກາງ", ຫຼື "ໜັກ"	Mild ເບົາ 1<br>Moderate ປານກາງ 2<br>Severe ໜັກ 3

6.5. Can you please explain the stages of the treatment? I will ask you step-by-step what you did, starting from the moment [you / the child] first experienced a discomfort. ທ່ານຊ່ວຍອະທິບາຍຂັ້ນຕອນໃນການຮັກສາການເຈັບເປັນຄັ້ງນັ້ນໃຫ້ເຮົາໄດ້ບໍ່? ເຮົາຈະຂໍຮັບຟັງໄປເປັນແຕ່ລະຂັ້ນຕອນ, ເລີ່ມຈາກເທື່ອທຳອິດທີ່ເລີ່ມຮູ້ສຶກບໍ່ສະບາຍ ຫຼື ໄດ້ຮັບອຸບັດຕິເຫດ

	**6.5.1.** Step 1 (detection) ຂັ້ນທີ 1 (ເມື່ອເລີ່ມຮູ້ສຶກບໍ່ສະບາຍ)	Step n

a) What kind of help or treatment did you get at this stage?<br>[***if unsure, specify***]<br>ທ່ານມີວິທີການຮັກສາແນວໃດໃນຂັ້ນຕອນນີ້<br>[***ຖ້າບໍ່ໝັ້ນໃຈໃຫ້ຂຽນລາຍລະອຽດໃນໝວດ "ອື່ນໆ"***]	Self-care (sleep, rest, medicine at home) ດູແລຕົວເອງ (ນອນ, ພັກຜ່ອນ, ກິນຢາຢູ່ບ້ານ) 1<br>Care from family and friends (full-time) ການເບິ່ງແຍງຈາກຄອບຄົວ ແລະ ໝູ່ເພື່ອນ (ເຕັມເວລາ) 2<br>Treated/cons. at shop / vendor selling drugs ໄດ້ຮັບການປິ່ນປົວຢູ່ຮ້ານຄ້າ/ຄົນຂາຍຢາ 3<br>Treated/consulted at a traditional healer ໄດ້ຮັບການປິ່ນປົວ/ປຶກສາຢູ່ໝໍພື້ນເມືອງ/ໝໍຜີ 4<br>Treated/cons. at a pharmacist ໄດ້ຮັບການປິ່ນປົວ/ປຶກສາກັບແພດຂາຍຢາ 5<br>Treated/cons. at priv. clinic/hospital ໄດ້ຮັບການປິ່ນປົວ/ປຶກສາທີ່ຄຣີນິກ/ໂຮງໝໍ 6<br>Treated/cons. at NGO clinic/service ໄດ້ຮັບການປິ່ນປົວ/ປຶກສາທີ່ຄຣີນິກບໍ່ສະແຫວງຫາຜົນກຳໄລ/ໂຮງໝໍ 7<br>Treated/cons. at health centre ໄດ້ຮັບການປິ່ນປົວ/ປຶກສາທີ່ສຸກສາລາ/ໂຮງໝໍນ້ອຍ 8<br>Treated/cons. at a public hospital ໄດ້ຮັບການປິ່ນປົວ/ປຶກສາທີ່ໂຮງໝໍເອກະຊົນ 9<br>Treated/cons. by a village health volunteer ໄດ້ຮັບການປິ່ນປົວ/ ອາສາສະໝັກສາທາລະນະສຸກບ້ານ (ອສບ) 10<br>Treated/cons. by an unregistered/local/informal/retired doctor ໄດ້ຮັບການປິ່ນປົວ/ປຶກສາໂດຍໝໍທີ່ບໍ່ມີໃບຢັ້ງຢືນ/ທ້ອງຖິ່ນ/ບໍ່ເປັນທາງການ/ບຳນານ 11<br>Treated/consulted at a traditional birth assistant ໄດ້ຮັບການປິ່ນປົວ/ປຶກສາຢູ່ໝໍຕຳແຍ 15<br>Cross-border care/ປະເທດໃກ້ຄຽງ 97<br>Other (incl. internet; specify)ອື່ນໆ (ປະກອບທັງ ອິນເຕີເນັດ; ລະບຸ _____ 99	1<br>2<br>3<br>4<br>5<br>6<br>7<br>8<br>9<br>10<br>11<br>15<br>99
b) Can you please name or describe all the medicines that you received or were prescribed during this step?<br>[***include medicine stored at home if "self-care at home"***]<br>[***continue for all medicines received, then complete Questions g to k for each medicine individually***]<br>ທ່ານໄດ້ໃຊ້ຢາຈັກຊະນິດໃນການປິ່ນປົວຂັ້ນຕອນນີ້?<br>[ຮວມຢາທີ່ມີເກັບຢູ່ເຮືອນດ້ວຍ ຖ້າຫາກຜູ້ໃຫ້ສຳພາດປົວຕົນເອງຢູ່ເຮືອນ] [ບັນທຶກຢາທຸກຊະນິດທີ່ຜູ້ໃຫ້ສຳພາດໄດ້ຮັບ ຈາກນັ້ນຕື່ມຂໍ້ມູນໃສ່ໃນຄຳຖາມ g ເຖິງ k ສຳລັບຢາແຕ່ລະຊະນິດ]<br>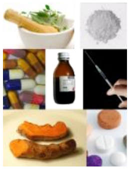	Medicine 1: Name/description: _____<br>Medicine n: Name/description: _____<br><br>ຢາຊະນິດທີ່ 1: ຊື່/ຄຳອະທິບາຍ: _____<br>ຢາຊະນິດທີ່ n: ຊື່/ຄຳອະທິບາຍ: _____	Medicine 1<br>Medicine n<br><br>ຢາຊະນິດທີ່ 1<br>ຢາຊະນິດທີ່ n
c) [***if not ignore/self-care***] How did the provider talk to you? [***mark all that aapply***] [ຖ້າບໍ່, ຂ້າມ] ຜູ້ສະໜອງການປິ່ນປົວໄດ້ໂອ້ລົມແນວໃດກັບທ່ານ? [ສາມາດໝາຍໄດ້ຫຼາຍກວ່າໜຶ່ງຂໍ້]	Supportive / understanding / praising ສະໜັບສະໜູນ/ເຂົ້າໃຈ/ຍົກຍ້ອງ 1<br>Nice / kind / friendly / positive 2 ດີ/ໃຈດີ/ເມດຕາ/ແງ່ດີ<br>Neutral / descriptive / unremarkable 3 ປານກາງ/ພໍບັນລະຍາຍໄດ້/ບໍ່ປະທັບໃຈປານໃດ<br>Criticising / negative ວິຈານ/ແງ່ລົບ 4<br>Scolding / raising their voice / yelling 5 ປ້ອຍດ່າ/ຂຶ້ນສຽງໃສ່/ຮ້ອງໃສ່<br>Other (specify) ອື່ນໆ (ລະບຸ): ___________ 6<br><br>Not applicable ບໍ່ແມ່ນທັງໝົດ 8<br>Don't know / prefer not to say ບໍ່ຮູ້/ບໍ່ຢາກກ່າວ 9	1<br>2<br>3<br>4<br>5<br>6 __<br><br>8<br>9
d) [***if not ignore/self-care***] How well did you feel taken care of by the provider? [ຖ້າບໍ່, ຂ້າມ]<br>ທ່ານຮູ້ສຶກດີສໍ່າໃດໃນການໄດ້ຮັບການເບິ່ງແຍງ/ປິ່ນປົວຈາກຜູ້ສະໜອງການປິ່ນປົວ?	Very well ດີຫຼາຍ 1<br>Well ດີ 2<br>So-so / indifferent / no opinion ປານກາງ/ບໍ່ແຕກຕ່າງ/ບໍ່ມີຄວາມຄິດເຫັນ 3<br>Not well ບໍ່ດີ 4<br>Unacceptably badly ບໍ່ສາມາດຮັບໄດ້ 5<br>Not relevant ບໍ່ກ່ຽວຂ້ອງ 8<br>Prefer not to say ບໍ່ຢາກກ່າວ 9	1<br>2<br>3<br>4<br>5<br><br>8<br>9
**6.6.** [Have you / has the child] now recovered from the illness/accident? ທ່ານດີຈາກອາການເຈັບເປັນ/ອຸບັດຕິເຫດແລ້ວບໍ່?	Yes ແມ່ນ 1<br>No ບໍ່ແມ່ນ 0<br>Don't know / prefer not to say ບໍ່ຮູ້ / ບໍ່ຢາກກ່າວ 9	
**6.7.** Was anybody of your personal relationships involved in providing advice or help <u>during the illness</u>? ທ່ານໄດ້ຮັບຄຳປຶກສາ ຫຼື ຄວາມຊ່ວຍເຫຼືອໃນດ້ານໃດໆ ກໍ່ຕາມຈາກບຸກຄົນທີ່ກ່ຽວຂ້ອງກັບທ່ານ <u>ລະຫວ່າງການເຈັບເປັນ</u>ຄັ້ງນີ້ ຫຼືບໍ່?	Yes ແມ່ນ 1<br>No ບໍ່ແມ່ນ 0<br>Don't know / prefer not to say ບໍ່ຮູ້ / ບໍ່ຢາກກ່າວ 9	
**6.7.1.** [***if yes***] How are these people related to you? [***Mark all that apply***] ຄວາມສຳພັນກັບທ່ານ [***ເລືອກໄດ້ຫຼາຍກວ່າ 1 ຄຳຕອບ***]	Spouse ຄູ່ແຕ່ງດອງ/ແຟນ 1<br>Parent ພໍ່ແມ່ 2<br>Child ລູກ 3<br>Sibling ອ້າຍນ້ອງ 4<br>Other relative ພີ່ນ້ອງ 5<br>Neighbour ເພື່ອນບ້ານ 6<br>Friend (if not neighbour) ໝູ່ (ທີ່ບໍ່ແມ່ນເພື່ອນບ້ານ) 7<br><br>Village chief / deputy ນາຍບ້ານ/ຮອງນາຍບ້ານ 21<br>Lao Front ແນວລາວສ້າງຊາດ 22<br>Lao Women's Union ສະຫະພັນແມ່ຍິງ 23<br>Lao Youth Union ຊາວໜຸ່ມ 24<br>Other authorities ແລະ ອຳນາດການປົກຄອງ ອື່ນໆ 25<br>Other villager ຊາວບ້ານຄົນອື່ນໆ 98	

	Other (specify) ____ ອື່ນໆ (ກະລຸນາບອກ)	99
**6.7.2.** [***if yes***] What kind of support did they provide? [***Mark all that apply***] ທ່ານໄດ້ຮັບການຊ່ວຍເຫຼືອຈາກບຸກຄົນເຫຼົ່ານີ້ແບບໃດ [***ເລືອກໄດ້ຫຼາຍກ່ວາ 1 ຄຳຕອບ***]	Providing healthcare/attending ໃຫ້ການປິ່ນປົວ/ເບິ່ງແຍງ	11
	Providing advice ໃຫ້ຄຳປຶກສາ	12
	Providing medicine ໃຫ້ຢາ	13
	Lending/granting money ໃຫ້ເງິນ ຫຼື ໃຫ້ຢືມເງິນ	21
	Transportation/Lending vehicle ອຳນວຍຄວາມສະດວກດ້ານການເດີນທາງ/ໃຫ້ຢືມລົດ	22
	Contacting family/friends ຕິດຕໍ່ຄອບຄົວ/ໝູ່	23
	Providing food ສະໜັບສະໜູນອາຫານ	31
	Helping with children/housework ຊ່ວຍເບິ່ງແຍງລູກຫລານຫຼືເດັກນ້ອຍ/ວຽກເຮືອນ	32
	Helping with jobs/agriculture (feeding animals/tending crops/covering shifts, etc.) ຊ່ວຍເຮັດວຽກ/ການກະສິກຳ (ຊ່ວຍເບິ່ງແຍງສັດລ້ຽງ/ເຮັດໄຮ່ເຮັດນາ/ເຮັດວຽກແທນ)	33
	Other (specify) _ ອື່ນໆ (ກະລຸນາບອກ) _____	99
**6.8.** [DELETED]		
xi. Interview end time ເວລາສິ້ນສຸດການສຳພາດ	[***time entered automatically***] [***ເວລາຖືກບັນທຶກໂດຍອັດຕະໂນມັດ***]	

Thank you very much for participating in this survey. ຂໍຂອບໃຈທ່ານທີ່ເຂົ້າຮ່ວມການສຳພາດໃນຄັ້ງນີ້

Part VII: Interviewer observations [to be completed by interviewer after interview]
ພາກທີ VII: ບັນທຶກຈາກການສັງເກດການຂອງຜູ້ສຳພາດ [*ຜູ້ສຳພາດຕື່ມຂໍ້ມູນສ່ວນນີ້ຫຼັງຈາກການສຳພາດ*]

xii. Was someone else present during the interview? [***mark all that apply***] ໃນລະຫວ່າງການສຳພາດໄດ້ມີບຸກຄົນອື່ນໆ ຢູ່ໃນບໍລິເວນສຳພາດ ຫຼືບໍ່? [ຕອບໄດ້ຫຼາຍກ່ວາ 1 ຕົວເລືອກ]	Survey supervisor ຫົວໜ້າທີມເກັບຂໍ້ມູນ	1
	Survey team member ສະມາຊິກທີມເກັບຂໍ້ມູນ	2
	Translator ນັກແປພາສາ	3
	Other household or family member ສະມາຊິກຄົນອື່ນໆ ໃນຄົວເຮືອນຫຼືຄອບຄົວ	4
	Medical practitioner ໝໍ	5
	Government officer ບຸກຄົນຈາກພາກລັດ	6
	Village chief / deputy ນາຍບ້ານ/ຮອງນາຍບ້ານ	21
	Lao Front ແນວລາວສ້າງຊາດ	22
	Lao Women's Union ສະຫະພັນແມ່ຍິງ	23
	Lao Youth Union ສູນກາງຊາວໜຸ່ມ	24
	Other authorities ແລະ ອຳນາດການປົກຄອງ ອື່ນໆ	25
	Other (specify) ____ ອື່ນໆ (ກະລຸນາບອກ) ____	7
	No one ບໍ່ມີບຸກຄົນອື່ນໃນບໍລິເວນສຳພາດລະຫວ່າງການສຳພາດ	0
xiii. What is your evaluation of the accuracy and trustworthiness of the informant's answers? ປະເມີນຄວາມຖືກຕ້ອງ ແລະ ຄວາມໜ້າເຊື່ອຖືຂອງຄຳຕອບຈາກການສຳພາດນີ້	Very good ດີຫຼາຍ	1
	Satisfactory ໜ້າເພິ່ງພໍໃຈ	2
	Doubtful ໜ້າສົງໄສ	3
	Very low ຕ່ຳຫຼາຍ	4
xiv. Were there any unusual circumstances during the interview? ມີເຫດການທີ່ບໍ່ປົກກະຕິໃດໜຶ່ງເກີດຂຶ້ນລະຫວ່າງການສຳພາດ ຫຼືບໍ່?	Please describe: ____________________________________<br>ກະລຸນາບອກເຫດການທີ່ບໍ່ປົກກະຕິນັ້ນໆ:___________________	

INTERVIEW GUIDES – COGNITIVE INTERVIEWS FOR SURVEY TESTING
ບົດສຳພາດ - ການສຳພາດຂະບວນການຄິດເພື່ອປັບປຸງແບບສອບຖາມ

Objective ຈຸດປະສົງ

To understand how survey participants interpret the survey questions and arrive at their answers. ເພື່ອເຂົ້າໃຈວ່າຜູ້ເຂົ້າຮ່ວມມີຄວາມເຂົ້າໃຈຄຳຖາມທີ່ໃຊ້ໃນແບບສອບຖາມແນວໃດ ແລະ ມີຄວາມຄິດແນວໃດກ່ອນຕັດສິນໃຈຕໍ່ຄຳຖາມແຕ່ລະຂໍ້

Notes ໝາຍເຫດ

- Total duration of each interview to be less than 45 minutes, up to 50 interviews. ໄລຍະເວລາທັງໝົດຂອງການສຳພາດແຕ່ລະຄັ້ງຈະໃຊ້ເວລາໜ້ອຍກວ່າ 45 ນາທີ ແລະ ການສຳພາດປະມານ 50 ການສຳພາດ/ຄັ້ງ.
- The interview format is semi-structured, meaning that it is a <u>conversation</u> around the survey questions. Probes are provided to guide the conversation.
  ຮູບແບບໃນການສຳພາດແມ່ນ ແບບເຄິ່ງໂຄງສ້າງ, ເຊິ່ງໝາຍຄວາມວ່າມັນເປັນການສົນທະນາທີ່ສອດຄ່ອງກັບຄຳຖາມຂອງການສຳຫຼວດ.
- Different community groups will be recruited into the interviews (i.e. the interview team should find those groups specifically) across 3-4 provinces: ກຸ່ມຊຸມຊົນຕ່າງໆຈະຖືກຄັດເລືອກເຂົ້າຮ່ວມໃນການສຳພາດ (ເຊັ່ນ: ທີມສຳພາດຄວນຄົ້ນຫາກຸ່ມເຫຼົ່ານັ້ນໂດຍສະເພາະ) ໃນ 3-4 ແຂວງ
    - (Recently) pregnant women (10-15) (ລ່າສຸດ) ແມ່ຍິງຖືພາ (10-15 ຄົນ)
    - Men/women of varying ages / education / ethnic groups / economic status (10-15) ຜູ້ຊາຍ/ແມ່ຍິງໃນກຸ່ມອາຍຸທີ່ແຕກຕ່າງກັນ/ການສຶກສາ/ກຸ່ມຊົນເຜົ່າ/ສະຖານະທາງເສດຖະກິດ (10-15 ຄົນ)
    - Healthcare workers who live in the village (5-10) ພະນັກງານແພດທີ່ອາໄສຢູ່ໃນບ້ານນັ້ນໆ (5-10 ຄົນ)
    - Village leaders (no more than 5) ຜູ້ນຳກຸ່ມບ້ານ (ບໍ່ເກີນ 5 ຄົນ)
    - As above, but after CONNECT M2 workshop (10-20) ຕາມທີ່ລະບຸດ້ານເທິງນັ້ນ, ແຕ່ຈະເປັນພາຍຫຼັງ CONNECT ເວີກຊ໋ອບ M2 (10-20)
- As a semi-structured interview, the interviewer can choose to select key questionnaire items / probing questions to focus the conversation (as long as it supports the objective). ອີງຕາມການສຳພາດແບບເຄິ່ງໂຄງສ້າງ, ຜູ້ສຳພາດສາມາດເລືອກເອົາຄຳຖາມທີ່ສຳຄັນ/ຄຳຖາມສຳຫຼວດເພື່ອສຸມໃສ່ການສົນທະນາ (ຕາບໃດທີ່ມັນສະໜັບສະໜູນຈຸດປະສົງ)
- Not all sections are relevant for everyone – e.g. M2 workshop module and questions or pregnancy. Feel free to focus interviews by relevance. ບໍ່ແມ່ນທຸກສ່ວນທີ່ຈະກ່ຽວກັບທຸກໆຄົນ-ເຊັ່ນ: ໂມດູນຂອງເວີກຊ໋ອບ M2 ແລະ ຄຳຖາມ ຫຼື ກ່ຽວກັບການຖືພາ. ຢ່າລັງເລທີ່ຈະສຸມໃສ່ການສຳພາດທີ່ມີຄວາມກ່ຽວຂ້ອງ.

Introduction ບົດນຳ

<Obtaining verbal informed consent in accordance with Oral Consent Script> <ດຳເນີນການຂໍຄຳຍິນຍອມໂດຍໃຊ້ຂໍ້ຄວາມສຳລັບອະທິບາຍວຽກຄົ້ນຄ້ວາ ແລະ ຂໍຄຳຍິນຍອມເຂົ້າຮ່ວມການຄົ້ນຄ້ວາດ້ວຍວາຈາ>

Thank you for agreeing to participate in this study. ຂອບໃຈທ່ານສຳລັບການຕົກລົງເຂົ້າຮ່ວມການຄົ້ນຄ້ວາໃນຄັ້ງນີ້

1. Let us start with a few questions about yourself. ເຮົາຈະເລີ່ມໂດຍຖາມຄຳຖາມກ່ຽວກັບຂໍ້ມູນສ່ວນຕົວຂອງທ່ານ
    a) How old are you? ອາຍຸທ່ານ
    b) [*record as observed*] Sex [ບັນທຶກຈາກວິຈາລະນະຍານຂອງຜູ້ສຳພາດ] ເພດ
    c) What is your level of education? ການສຶກສາສູງສຸດຂອງທ່ານ
    d) What is your ethnicity? ເຊື້ອຊາດ
    e) Are you aware that there is a health centre that serves this village? ທ່ານຮູ້ບໍ່ວ່າມີສຸກສາລາ/ໂຮງໝໍນ້ອຍໃນໝູ່ບ້ານແຫ່ງນີ້?

f) [If yes] Have you ever been to the health centre? [ຖ້າແມ່ນ] ທ່ານເຄີຍໄປສຸກສາລາ ຫຼື ໂຮງໝໍນ້ອຍບໍ່?

2. I will now ask you a few questions to better understand your impression of the health centre. It does not matter if you have been to the health centre or not; we are interested in what you think and believe and what you have heard, even if it is not based on personal experience. ຕໍ່ໄປນີ້ ຂ້າພະເຈົ້າຈະຖາມຄໍາຖາມຈໍານວນໜຶ່ງ ເພື່ອເຂົ້າໃຈເຖິງຄວາມປະທັບໃຈຂອງທ່ານຕໍ່ກັບສຸກສາລາ/ໂຮງໝໍນ້ອຍ.
ບໍ່ຈໍາເປັນວ່າທ່ານເຄີຍໃຊ້ບໍລິການສຸກສາລາ/ໂຮງໝໍນ້ອຍມາກ່ອນຫຼືບໍ່; ພວກເຮົາສົນໃຈໃນສິ່ງທ່ານຄິດ ແລະ ຄວາມເຊື່ອ ແລະ ເຄີຍໄດ້ຍິນມາ, ເຖິງແມ້ວ່າຈະບໍ່ໄດ້ອີງຕາມປະສົບການສ່ວນຕົວກໍຕາມ.

What are you thinking about when you hear the following questions? ທ່ານມີຄວາມຄິດຫຍັງແດ່ເມື່ອທ່ານໄດ້ຍິນຄໍາຖາມຕໍ່ໄປນີ້
[Note to interviewer: discuss each question to see how participants understands it and what their answer means – probes are below. Use these quotes to stimulate the conversation while going through the survey items in the table below.] [ໝາຍເຫດເຖິງຜູ້ສໍາພາດ: ສົນທະນາແຕ່ລະຄໍາຖາມເພື່ອສັງເກດເບິ່ງວ່າຜູ້ເຂົ້າຮ່ວມເຂົ້າໃຈແນວໃດ ແລະ ຄໍາຕອບຂອງພວກເຂົາໝາຍຄວາມວ່າແນວໃດ-ການຖາມຕອບດ້ານລຸ່ມນີ້. ໃຊ້ຄໍາເວົ້າເຫຼົ່ານີ້ເພື່ອກະຕຸ້ນການສົນທະນາ ໃນຂະນະທີ່ເຮັດແບບສໍາຫຼວດຕາມຕາຕະລາງໃນດ້ານລຸ່ມນີ້.]

a) Do you feel that these questions give us a good idea of how much you trust the local health centre? ທ່ານຮູ້ສຶກວ່າຄໍາຖາມເຫຼົ່ານີ້ເຮັດໃຫ້ທ່ານເຂົ້າໃຈດີວ່າ ທ່ານໄວ້ເນື້ອເຊື່ອໃຈໂຮງໝໍນ້ອຍໃນທ້ອງຖິ່ນໜ້ອຍຫຼາຍປານໃດ?
b) Were some of these questions difficult or not really appropriate? ທ່ານພົບວ່າຄໍາຖາມເຫຼົ່ານີ້ມີຄວາມຍາກ - ບໍ່ເໝາະສົມ ຫຼື ບໍ່?
c) Did you think about specific situations when answering the question? What did you relate to? ທ່ານໄດ້ຄິດເຖິງສະຖານະການໃດໜຶ່ງເປັນການສະເພາະ ຫຼື ບໍ່ ໃນຂະນະທີ່ທ່ານຕອບຄໍາຖາມ? ທ່ານຄິດເຫັນເຫດການໃດ?
d) What time period were you thinking of? ທ່ານຄິດເຫັນເຖິງຊ່ວງເວລາໃດ?
e) Can you explain the idea of "trust" towards the health centre from your perspective, and what really matters for you? ທ່ານສາມາດອະທິບາຍແນວຄວາມຄິດຂອງ "ຄວາມໄວ້ເນື້ອເຊື່ອໃຈ" ຈາກທັດສະນະຄະຕິຂອງທ່ານທີ່ມີຕໍ່ໂຮງໝໍນ້ອຍ ແລະ ອັນໃດທີ່ມີຄວາມສໍາຄັນສໍາລັບທ່ານ?
f) Would you generally say that you do trust the health centre, or rather not? Why is that so, from your perspective? ໂດຍທົ່ວໄປແລ້ວທ່ານຈະບອກວ່າທ່ານມີຄວາມໄວ້ເນື້ອເຊື່ອໃຈຕໍ່ໂຮງໝໍນ້ອຍ ຫຼື ບໍ່?ເປັນຫຍັງຄືວ່າແນວນັ້ນ, ອີງຕາມຄວາມຄິດຂອງທ່ານ?
g) As I have said before, these survey questions are not intended to measure your knowledge. Did you feel tested or pressured being asked these questions at all? ຕາມທີ່ພວກເຮົາໄດ້ແຈ້ງທ່ານມາແລ້ວທໍາອິດກ່ຽວກັບຄໍາຖາມເຫຼົ່ານີ້ທີ່ບໍ່ໄດ້ມີຈຸດປະສົງເພື່ອວັດແທກຄວາມຮູ້ຂອງທ່ານ . ແຕ່ເຖິງຢ່າງໃດກໍຕາມ, ທ່ານຮູ້ສຶກຖືກທົດສອບ ຫຼື ຮູ້ສຶກກົດດັນໃນເວລາຕອບຄໍາຖາມເຫຼົ່ານີ້ບໍ່?

**1.**	Can you talk comfortably with the staff at the health centre?<br>ທ່ານສາມາດລົມກັບພະນັກງານແພດປະຈໍາໂຮງໝໍນ້ອຍໄດ້ຢ່າງເປັນກັນເອງ ແລະ ສະບາຍໃຈບໍ່?	Yes ແມ່ນ 1<br>No ບໍ່ແມ່ນ 0<br>Don't know / prefer not to say ບໍ່ຮູ້ / ບໍ່ຢາກກ່າວ 9
**2.**	Can you give me an example of the kind of stories you heard about the health centre or its staff? (give examples through the answer categories)<br>ຈົ່ງຍົກຕົວຢ່າງເລື່ອງລາວທີ່ທ່ານເຄີຍໄດ້ຍິນມາກ່ຽວກັບສຸກສາລາ/"ໂຮງໝໍນ້ອຍ ຫຼື ກ່ຽວກັບພະນັກງານແພດ-ໝໍໃຫ້ຟັງໄດ້ບໍ່? (ໃຫ້ຕົວຢ່າງຜ່ານແຕ່ລະປະເພດຂອງຄໍາຕອບ)	Bad (scolding, mean, extracting money, refusing service, closed when needed) ບໍ່ດີ (ຮ້ອງໃສ່, ໃຈຮ້າຍ, ຖາມເອົາເງິນ, ປະຕິເສດການປິ່ນປົວ, ປິດເມື່ອຕ້ອງການ 0<br>Neutral (descriptive, services being provided, location, opening times) ທໍາມະດາ (ໄດ້ຮັບການບໍລິການ, ເວລາເປີດປົກກະຕິ, ການອະທິບາຍດີ) 1<br>Good (caring staff, solving problems, supportive, friendly) ດີ (ໝໍດູແລດີ, ແກ້ໄຂບັນຫາໄດ້, ສະໜັບສະໜູນ, ເປັນກັນເອງ) 2<br>Don't know / prefer not to say ບໍ່ຮູ້ / ບໍ່ຢາກກ່າວ 9<br>Story ອະທິບາຍເລື່ອງລາວ:<br>___________________________________

**3.** Is the health centre a place where staff treat you badly? ສຸກສາລາ/ໂຮງໝໍນ້ອຍແມ່ນສະຖານທີ່ທີ່ພະນັກງານປະຕິບັດກັບທ່ານບໍ່ດີ/ເວົ້າບໍ່ມ່ວນບໍ່?	Yes ແມ່ນ 1<br>No ບໍ່ແມ່ນ 0<br>Don't know / prefer not to say ບໍ່ຮູ້ / ບໍ່ຢາກກ່າວ 9
**4.** Have you ever experienced/ heard of any kind of discrimination at the health centre? ທ່ານເຄີຍມີປະສົບການ ຫຼື ໄດ້ຍິນກ່ຽວກັບການຈໍາແນກການບໍລິການ/ເລືອກປະຕິບັດໃນໂຮງໝໍນ້ອຍບໍ່?	Yes ແມ່ນ 1<br>No ບໍ່ແມ່ນ 0<br>Don't know / prefer not to say ບໍ່ຮູ້ / ບໍ່ຢາກກ່າວ 9
**4.1.** [***Round 2 only and if yes***] Was this the case in the last three months? [ຮອບສອງເທົ່ານັ້ນ] ມັນແມ່ນກໍລະນີທີ່ເກີດຂຶ້ນໃນໄລຍະສາມເດືອນທີ່ຜ່ານມາ?	Yes ແມ່ນ 1<br>No ບໍ່ແມ່ນ 0<br>Don't know / prefer not to say ບໍ່ຮູ້ / ບໍ່ຢາກກ່າວ 9
**4.2.** [***all Round 1 plus < Round 2 and if yes>***] Example if yes ຖ້າແມ່ນຊ່ວຍຍົກຕົວຢ່າງ	Example if yes ຖ້າແມ່ນຊ່ວຍຍົກຕົວຢ່າງ: ______________________________
**5.** Have you ever been refused/ heard people being refused treatment from the staff at the health centre? ແພດໝໍເຄີຍປະຕິເສດການປິ່ນປົວ ຫຼື ເລືອກປະຕິບັດກັບທ່ານ ຫຼື ຄົນທີ່ທ່ານຮູ້ຈັກຫຼືບໍ່	Yes ແມ່ນ 1<br>No ບໍ່ແມ່ນ 0<br>Don't know / prefer not to say ບໍ່ຮູ້ / ບໍ່ຢາກກ່າວ 9
**5.1.** [***Round 2 only and if yes***] Was this the case in the last three months? [ຮອບສອງເທົ່ານັ້ນ] ມັນແມ່ນກໍລະນີທີ່ເກີດຂຶ້ນໃນໄລຍະສາມເດືອນທີ່ຜ່ານມາ?	Yes ແມ່ນ 1<br>No ບໍ່ແມ່ນ 0<br>Don't know / prefer not to say ບໍ່ຮູ້ / ບໍ່ຢາກກ່າວ 9
**5.2.** [***all Round 1 plus < Round 2 and if yes>***] If yes, what was the reason? ຖ້າເຄີຍ, ເຫດຜົນຍ້ອນຫຍັງ?	Response: ______________________________
**6.** If you went to the health centre right at this moment with a motorbike accident, do you think they would be able to provide you with primary treatment or first aid? ຖ້າຫາກທ່ານໄປສຸກສາລາ/ໂຮງໝໍນ້ອຍ ເມື່ອຕອນເກີດອຸບັດຕິເຫດລົດຈັກ ທ່ານຄິດວ່າພະນັກງານແພດໂຮງໝໍນ້ອຍ/ໂຮງໝໍເມືອງຈະສາມາດສະໜອງການປິ່ນປົວຂັ້ນທໍາອິດໃຫ້ທ່ານໄດ້ ຫຼື ບໍ່?	Yes ແມ່ນ 1<br>No ບໍ່ແມ່ນ 0<br>Don't know / prefer not to say ບໍ່ຮູ້ / ບໍ່ຢາກກ່າວ 9
**7.** Do you feel confident in their medical skills? ທ່ານຮູ້ສຶກໝັ້ນໃຈໃນຄວາມຊໍານິຊໍານານ/ປະສົບການໃນການປິ່ນປົວຂອງພະນັກງານແພດປະຈໍາໂຮງໝໍນ້ອຍບໍ່?	Yes (confidently) ແມ່ນ (ຢ່າງໝັ້ນໃຈ) 1<br>So-so / no strong opinion ທໍາມະດາ/ປານກາງ 3<br>No ບໍ່ແມ່ນ 0<br>Don't know / prefer not to say ບໍ່ຮູ້ / ບໍ່ຢາກກ່າວ 9
**8.** Do health staff ask for money for extra services, ask for money for services which should be free or to buy medicines from their houses? ພະນັກງານສຸກສາລາໄດ້ຖາມເອົາເງິນສໍາລັບການບໍລິການພິເສດບໍ່, ຖາມເອົາເງິນຈາກການບໍລິການທີ່ບໍ່ຄວນເສຍຄ່າ ຫຼື ພະນັກງານແພດເຄີຍແນະນໍາໃຫ້ໄປຊື້ຢາທີ່ບ້ານຂອງເຂົາຫຼືບໍ່?	Yes ແມ່ນ 1<br>No ບໍ່ແມ່ນ 0<br>Don't know / prefer not to say ບໍ່ຮູ້ / ບໍ່ຢາກກ່າວ 9
**8.1.** Explain if there is anything unusual to add (e.g. felt need to offer money or gifts for better care, or if you don't give extra money, do you get bad care?) ອະທິບາຍຖ້າມີສິ່ງທີ່ຢາກເພີ່ມຕື່ມ:(ຕຍ: ຮູ້ສຶກວ່າຕ້ອງໄດ້ເອົາເງິນໃຫ້ທ່ານໝໍຈຶ່ງຈະໄດ້ຮັບການບໍລິການດີຂຶ້ນ, ຫຼື ຖ້າບໍ່ໃຫ້ເງິນ, ຈະໄດ້ຮັບການບໍລິການທີ່ບໍ່ດີ?	Explain if there is anything unusual to add<br>ອະທິບາຍຖ້າມີສິ່ງທີ່ຢາກເພີ່ມຕື່ມ: __________
**9.** If you don't give extra money, do you get bad care? ຖ້າທ່ານບໍ່ໄດ້ເອົາເງິນໃຫ້, ທ່ານຈະຖືກດູແລບໍ່ດີບໍ່?	Yes ແມ່ນ 1<br>No ບໍ່ແມ່ນ 0<br>Don't know / prefer not to say ບໍ່ຮູ້ / ບໍ່ຢາກກ່າວ 9
**10.** Overall, would you say you rather trust or distrust the healthcare centre for your personal healthcare? ໂດຍລວມແລ້ວ, ທ່ານມີຄວາມໄວ້ເນື້ອເຊື່ອໃຈໃນການເຂົ້າຮັບການບໍລິການປິ່ນປົວ/ດູແລສຸຂະພາບສ່ວນຕົວຂອງທ່ານກັບສຸກສາລາ/ໂຮງໝໍນ້ອຍ ຫຼື ບໍ່?	Rather trust ໄວ້ເນື້ອເຊື່ອໃຈ 2<br>Neutral / no opinion ໄວ້ເນື້ອເຊື່ອໃຈປານກາງ/ບໍ່ມີຄໍາເຫັນ 1<br>Rather distrust ບໍ່ໄວ້ເນື້ອເຊື່ອໃຈ 0<br>Don't know / prefer not to say ບໍ່ຮູ້ / ບໍ່ຢາກກ່າວ 9

3. I would now like to ask you a few questions specifically about your involvement in the healthcare services in this village. As before, it would really help us to understand what you think about when answering our questions. ຂ້າພະເຈົ້າຢາກສອບຖາມຕື່ມ 2-3 ຄໍາຖາມໂດຍເຈາະຈົງກ່ຽວກັບການມີສ່ວນຮ່ວມຂອງທ່ານໃນການບໍລິການດ້ານສຸຂະພາບພາຍໃນບ້ານ. ດັ່ງທີ່ຜ່ານມາ, ມັນຈະຊ່ວຍເຮັດໃຫ້ພວກເຮົາເຂົ້າໃຈໃນສິ່ງທີ່ທ່ານຄິດກ່ຽວໃນເວລາທີ່ຕອບຄໍາຖາມຂອງພວກເຮົາ.

[Note to interviewer: discuss each question to see how participants understands it and what their answer means – probes are below. Use these quotes to stimulate the conversation while going through the survey items in the table below.] [ໝາຍເຫດເຖິງຜູ້ສໍາພາດ: ສົນທະນາແຕ່ລະຄໍາຖາມເພື່ອສັງເກດເບິ່ງວ່າຜູ້ເຂົ້າຮ່ວມເຂົ້າໃຈແນວໃດ ແລະ ຄໍາຕອບຂອງພວກເຂົາໝາຍຄວາມວ່າແນວໃດ-ການຖາມຕອບດ້ານລຸ່ມນີ້. ໃຊ້ຄໍາເວົ້າເຫຼົ່ານີ້ເພື່ອກະຕຸ້ນການສົນທະນາໃນຂະນະທີ່ເຮັດແບບສໍາຫຼວດຕາມຕາຕະລາງໃນດ້ານລຸ່ມນີ້.]

a) Can you please explain your answers to me? ທ່ານຊ່ວຍອະທິບາຍຄໍາຕອບຂອງທ່ານໃຫ້ເຮົາຟັງໄດ້ບໍ່?
b) Were there questions that you found difficult or not really appropriate? ທ່ານພົບວ່າຄໍາຖາມເຫຼົ່ານີ້ມີຄວາມຍາກ - ບໍ່ເໝາະສົມ ຫຼື ບໍ່?
c) Did you think about specific situations when answering the question? What did you relate to? ທ່ານໄດ້ຄິດເຖິງສະຖານະການໃດໜຶ່ງເປັນການສະເພາະ ຫຼື ບໍ່ ໃນຂະນະທີ່ທ່ານຕອບຄໍາຖາມ? ທ່ານຄິດເຫັນເຫດການໃດ?
d) What time period were you thinking of? ທ່ານຄິດເຫັນເຖິງຊ່ວງເວລາໃດ?
e) Do you think it matters for villagers to have some involvement in local healthcare provision? ທ່ານຄິດວ່າມັນເປັນເລື່ອງສໍາຄັນບໍ່ທີ່ຈະໃຫ້ປະຊາຊົນໃນບ້ານມີສ່ວນຮ່ວມໃນການສະໜອງການປິ່ນປົວຢູ່ໂຮງໝໍນ້ອຍໃນທ້ອງຖິ່ນ?

1.a) Do you think that you have a role in making this a "healthy village"? ທ່ານຄິດວ່າທ່ານມີໜ້າທີ່ໃນການເຮັດໃຫ້ "ຊຸມຊົນມີສຸຂະພາບດີ" ຫຼືບໍ່?	Yes ແມ່ນ 1<br>No ບໍ່ແມ່ນ 0<br>Don't know / prefer not to say ບໍ່ຮູ້ / ບໍ່ຢາກກ່າວ9
2.a) Do you think that you can contribute to making the village healthy? ທ່ານຄິດວ່າທ່ານສາມາດປະກອບສ່ວນໃນການເຮັດໃຫ້ "ຊຸມຊົນມີສຸຂະພາບດີ" ຫຼືບໍ່?	Yes ແມ່ນ 1<br>No ບໍ່ແມ່ນ 0<br>Don't know / prefer not to say ບໍ່ຮູ້ / ບໍ່ຢາກກ່າວ9
3.a) Would it be important for you if you could do that? ມັນຈະສໍາຄັນກັບທ່ານຫຼືບໍ່ ຖ້າຫາກທ່ານສາມາດເຮັດໃຫ້ "ຊຸມຊົນມີສຸຂະພາບດີ" ໄດ້?	Yes ແມ່ນ 1<br>No ບໍ່ແມ່ນ 0<br>Don't know / prefer not to say ບໍ່ຮູ້ / ບໍ່ຢາກກ່າວ9
1.b) Do you think that you have a role in making the health centre services better? ທ່ານຄິດວ່າທ່ານມີໜ້າທີ່ໃນການປະກອບສ່ວນເຮັດໃຫ້ການບໍລິການສຸຂະພາບຢູ່ສຸກສາລາດີຂຶ້ນ ຫຼືບໍ່?	Yes ແມ່ນ 1<br>No ບໍ່ແມ່ນ 0<br>Don't know / prefer not to say ບໍ່ຮູ້ / ບໍ່ຢາກກ່າວ9
2.b) Do you think that you can contribute to making the health centre services better? ທ່ານຄິດວ່າທ່ານສາມາດປະກອບສ່ວນໃນການເຮັດໃຫ້ການບໍລິການຢູ່ສຸກສາລາດີຂຶ້ນຫຼືບໍ່?	Yes ແມ່ນ 1<br>No ບໍ່ແມ່ນ 0<br>Don't know / prefer not to say ບໍ່ຮູ້ / ບໍ່ຢາກກ່າວ9
3.b) Would it be important for you if you could do that? ມັນຈະສໍາຄັນກັບທ່ານຫຼືບໍ່ ຖ້າຫາກທ່ານສາມາດປະກອບສ່ວນໃນການເຮັດໃຫ້ການບໍລິການຢູ່ສຸກສາລາດີຂຶ້ນໄດ້?	Yes ແມ່ນ 1<br>No ບໍ່ແມ່ນ 0<br>Don't know / prefer not to say ບໍ່ຮູ້ / ບໍ່ຢາກກ່າວ9
4. Based on your impressions and experiences, do you think that the health centre offers the kind of care that this community needs? ອີງຕາມຄວາມປະທັບໃຈ ແລະ ປະສົບການຂອງທ່ານ, ທ່ານຄິດວ່າສຸກສາລາ/ໂຮງໝໍນ້ອຍສະໜອງປະເພດຂອງການປິ່ນປົວທີ່ຊຸມຊົນນີ້ຕ້ອງການຫຼືບໍ່?	Yes ແມ່ນ 1<br>No ບໍ່ແມ່ນ 0<br>Don't know / prefer not to say ບໍ່ຮູ້ / ບໍ່ຢາກກ່າວ9
5. Can you give me an example for your response to the previous question?ທ່ານຈົ່ງຍົກຕົວຢ່າງຕໍ່ກັບຄໍາຕອບຂອງທ່ານຂອງຄໍາຖາມກ່ອນໜ້ານີ້ໄດ້ບໍ່?	Example: ___________________________

4. Thank you. Now I would like to ask you a few more questions about the COVID pandemic in particular. ຂໍຂອບໃຈ. ຕໍ່ໄປພວກເຮົາຢາກຂໍຖາມຕື່ມ 2-3 ຄໍາຖາມ ກ່ຽວກັບໂລກລະບາດຂອງພະຍາດໂຄວິດໂດຍສະເພາະ

[Note to interviewer: discuss each question to see how participants understands it and what their answer means – probes are below. Use these quotes to stimulate the conversation while going through the survey items in the table below.] [ໝາຍເຫດເຖິງຜູ້ສໍາພາດ: ສົນທະນາແຕ່ລະຄໍາຖາມເພື່ອສັງເກດເບິ່ງວ່າຜູ້ເຂົ້າຮ່ວມເຂົ້າໃຈແນວໃດ ແລະ ຄໍາຕອບຂອງພວກເຂົາໝາຍຄວາມວ່າແນວໃດ-ການຖາມຕອບດ້ານລຸ່ມນີ້. ໃຊ້ຄໍາເວົ້າເຫຼົ່ານີ້ເພື່ອກະຕຸ້ນການສົນທະນາ ໃນຂະນະທີ່ເຮັດແບບສໍາຫຼວດຕາມຕາຕະລາງໃນດ້ານລຸ່ມນີ້.]

a) Can you please explain your answers to me? ທ່ານຊ່ວຍອະທິບາຍຄໍາຕອບຂອງທ່ານໃຫ້ເຮົາຟັງໄດ້ບໍ່?
b) Were there questions that you found difficult or not really appropriate? ທ່ານພົບວ່າຄໍາຖາມເຫຼົ່ານັ້ນມີຄວາມຍາກ - ບໍ່ເໝາະສົມ ຫຼື ບໍ່?
c) Did you think about specific situations when answering the question? What did you relate to? ທ່ານໄດ້ຄິດເຖິງສະຖານະການໃດໜຶ່ງເປັນການສະເພາະ ຫຼື ບໍ່ ໃນຂະນະທີ່ທ່ານຕອບຄໍາຖາມ? ທ່ານຄິດເຫັນເຫດການໃດ?
d) What time period were you thinking of? ທ່ານຄິດເຫັນເຖິງຊ່ວງເວລາໃດ?
e) Did the COVID-19 pandemic over the past two years change how you could use government healthcare like hospitals and health centres? If so, can you please tell us how? ການລະບາດຂອງເຊື້ອພະຍາດ COVID-19

ໃນສອງປີທີ່ຜ່ານມາໄດ້ປ່ຽນແປງ ຫຼື ສິ່ງຜົນກະທົບຕໍ່ກັບການເຂົ້າຮັບການປິ່ນປົວຢູ່ສະຖາບັນການປິ່ນປົວຂອງລັດ ເຊັ່ນວ່າ ໂຮງໝໍ ຫຼື ສຸກສາລາ/ໂຮງໝໍນ້ອຍຫຼືບໍ່? ຖ້າກະທົບ ທ່ານສາມາດບອກພວກເຮົາໄດ້ບໍ່ວ່າກະທົບທ່ານແບບໃດ?

f) Could you explain a little bit what COVID and the pandemic has meant to you personally? ທ່ານຊ່ວຍອະທິບາຍໜ້ອຍໜຶ່ງໄດ້ບໍ່ວ່າສຳລັບຕົວທ່ານແລ້ວ ພະຍາດໂຄວິດ ແລະ ໂລກລະບາດໃຫຍ່ມີຄວາມໝາຍຄືແນວໃດ? (ພະຍາດໂຄວິດແມ່ນຫຍັງ?)

g) Could you explain your experience in receiving and following government health advice about COVID over the last year? ໃຫ້ທ່ານຊ່ວຍອະທິບາຍປະສົບການໃນການຮັບຄຳແນະນຳ ແລະ ປະຕິບັດຕາມຄຳແນະນຳດ້ານສຸຂະພາບຂອງລັດຖະບານ ກ່ຽວກັບພະຍາດໂຄວິດໃນປີທີ່ຜ່ານມາໄດ້ ຫຼື ບໍ່?

**1.**	Do you know anyone who has ever had COVID? (including yourself)<br>ທ່ານເຄີຍຮູ້ຈັກບຸກຄົນອື່ນທີ່ເຄີຍຕິດເຊື້ອພະຍາດໂຄວິດ ຫຼື ບໍ່? (ລວມເຖິງຕົວທ່ານເອງ)	Yes ແມ່ນ 1<br>No ບໍ່ແມ່ນ 0<br>Don't know / prefer not to say ບໍ່ຮູ້ / ບໍ່ຢາກກ່າວ 9
**2.**	From where do you receive information about COVID? [***mark all that apply***]<br>ໄດ້ຮັບຂໍ້ມູນກ່ຽວກັບໂຄວິດຈາກຊ່ອງທາງໃດ? **[ເລືອກໄດ້ຫຼາຍກວ່າ 1 ຂໍ້]**	Family members ສະມາຊິກໃນຄອບຄົວ 1<br>Other villagers ສະມາຊິກໃນກຸ່ມບ້ານ 2<br>Village leader / loudspeaker ຜູ້ນຳຂັ້ນບ້ານ/ການປະກາດໂທລະໂຄ່ງ 3<br>Temple ວັດ 4<br>Church ໂບດ 5<br>Lao Front ແນວລາວສ້າງຊາດ 6<br>Lao Women's Union ສະຫະພັນແມ່ຍິງ 7<br>Lao Youth Union ຊາວໜຸ່ມ 8<br>Other authorities ແລະ ອຳນາດການປົກຄອງ ອື່ນໆ 9<br><br>Posters ໃບປິວ 10<br>Newspaper ໜັງສືພິມ 11<br>TV / radio ໂທລະພາບ/ວິທະຍຸ 12<br>Social media ສື່ສັງຄົມອອນລາຍ 13<br>Other online websites ເວັບໄຊອອນລາຍອື່ນໆ 14<br><br>Shop / vendor selling drugs **ຮ້ານຄ້າ/ຮ້ານຂາຍຢາທົ່ວໄປ** 20<br>Traditional healer **ໝໍພື້ນເມືອງ/ໝໍຜີ** 21<br>Pharmacist **ແພດການຢາ** 22<br>Private clinic/hospital **ຄຣີນິກເອກະຊົນ/ໂຮງໝໍ** 23<br>NGO clinic/service **ຄຣີນິກຂອງອົງການຈັດຕັ້ງສາກົນ/ການບໍລິການ** 24<br>Health centre **ສຸກສາລາ/ໂຮງໝໍນ້ອຍ** 25<br>Public hospital **ໂຮງໝໍໃຫຍ່ (ໂຮງໝໍລັດ)** 26<br>Village health volunteer ອາສາສະໝັກສາທາລະນະສຸກບ້ານ (ອສບ) 27<br>Unregistered/local/informal/retired doctor 28<br>**ໝໍບໍ່ມີໃບຢັ້ງຢືນແພດ/ທ້ອງຖິ່ນ/ບໍ່ເປັນທາງການ/ໝໍບໍານານ**<br>Traditional birth assistant **ໝໍຕຳແຍ** 29<br>Others 99
**3.**	Have you been vaccinated against COVID?<br>ທ່ານເຄີຍສັກຢາຕ້ານພະຍາດໂຄວິດບໍ່?	Yes ແມ່ນ 1<br>No ບໍ່ແມ່ນ 0<br>Don't know / prefer not to say ບໍ່ຮູ້ / ບໍ່ຢາກກ່າວ 9
**3.1.**	[***if yes to 4.3***] How many vaccine doses did you receive? [ຖ້າແມ່ນ ຂໍ້4.3]ທ່ານໄດ້ຮັບວັກຊີນຈັກເຂັມແລ້ວ?	0 | 1 | 2 | 3 | 4 | 5 | >5<br>Don't know / prefer not to say ບໍ່ຮູ້ / ບໍ່ຢາກກ່າວ 9
**3.2.**	[***if no to 4.3***] Why were you not vaccinated? [ຖ້າບໍ່ແມ່ນ ໃນຂໍ້4.3] ເປັນຫຍັງທ່ານຈຶ່ງບໍ່ສັກຢາ?	Reason ເຫດຜົນ: ______________________
**3.3.**	What would be the optimal number of COVID vaccine doses for you? ຈຳນວນວັກຊີນ COVID ທີ່ດີທີ່ສຸດສຳລັບເຈົ້າແມ່ນຈັກເຂັມ?	0 | 1 | 2 | 3 | 4 | 5 | >5<br>Don't know / prefer not to say ບໍ່ຮູ້ / ບໍ່ຢາກກ່າວ 9
**4.**	Are you worried to disclose your COVID-19 status with anyone if you get it in the future? ທ່ານກັງວົນທີ່ຈະເຜີຍແຜ່ສະຖານະຂອງຕົນເອງ ຫຼື ບໍ່ ຖ້າຫາກທ່ານເກີດຕິດເຊື້ອໄວຣັສ COVID-19?	Yes ແມ່ນ 1<br>No ບໍ່ແມ່ນ 0<br>Don't know / prefer not to say ບໍ່ຮູ້ / ບໍ່ຢາກກ່າວ 9

5. [women in childbearing age] Thank you. Now I would like to ask you a few more questions that relate to maternal healthcare, but those are specific for women who are currently pregnant or recently gave birth. [ແມ່ຍິງທີ່ຢູ່ໃນຊ່ວງເວລາການເກີດລູກ] ຂໍຂອບໃຈ. ຕໍ່ໄປພວກເຮົາຈະສອບຖາມຕື່ມ 2-3 ຄຳຖາມທີ່ກ່ຽວຂ້ອງກັບການເບິ່ງແຍງສຸຂະພາບຂອງແມ່, ແຕ່ຄຳຖາມເຫຼົ່ານັ້ນຈະເຈາະຈົງສະເພາະ ສຳລັບແມ່ຍິງທີ່ເກີດລູກໃໝ່ກຳລັງຖືພາ ຫຼື ເກີດລູກເມື່ອບໍ່ດົນມານີ້

Firstly, did you deliver a baby in the past 12 months or are you currently pregnant? ທ່ານໄດ້ເກີດລູກເມື່ອ 12 ເດືອນທີ່ຜ່ານມາ ຫຼື ຂະນະນີ້ໄດ້ກຳລັງຖືພາບໍ່? [if not, go to next section on M2 workshop or end interview]

[Note to interviewer: discuss each question to see how participants understands it and what their answer means – probes are below. Use these quotes to stimulate the conversation while going through the survey items in the table below.] [ໝາຍເຫດເຖິງຜູ້ສຳພາດ: ສົນທະນາແຕ່ລະຄຳຖາມເພື່ອສັງເກດເບິ່ງວ່າຜູ້ເຂົ້າຮ່ວມເຂົ້າໃຈແນວໃດ ແລະ ຄຳຕອບຂອງພວກເຂົາໝາຍຄວາມວ່າແນວໃດ-ການຖາມຕອບດ້ານລຸ່ມນີ້. ໃຊ້ຄຳເວົ້າເຫຼົ່ານີ້ເພື່ອກະຕຸ້ນການສົນທະນາ ໃນຂະນະທີ່ເຮັດແບບສຳຫຼວດຕາມຕາຕະລາງໃນດ້ານລຸ່ມນີ້.]

a) Can you please explain your answers to me? ທ່ານສາມາດອະທິບາຍຄຳຕອບຂອງທ່ານໃຫ້ເຮົາຟັງໄດ້ບໍ່?
b) Were there questions that you found difficult or not really appropriate? ທ່ານພົບວ່າຄຳຖາມເຫຼົ່ານັ້ນມີຄວາມຍາກ - ບໍ່ເໝາະສົມ ຫຼື ບໍ່?
c) Did you feel comfortable talking about your pregnancy? ທ່ານໄດ້ຮູ້ສຶກສະບາຍໃຈທີ່ຈະລົມກ່ຽວກັບການຖືພາຂອງທ່ານ ຫຼື ບໍ່?
d) Did you think about specific situations when answering the question? What did you relate to? ທ່ານໄດ້ຄິດເຖິງສະຖານະການໃດໜຶ່ງເປັນການສະເພາະ ຫຼື ບໍ່ ໃນຂະນະທີ່ທ່ານຕອບຄຳຖາມ? ທ່ານຄິດເຫັນເຫດການໃດ?
e) Can you explain to me who advised and accompanied you during your pregnancy? What was the process like? ທ່ານຊ່ວຍອະທິບາຍໃຫ້ເຮົາໄດ້ບໍ່ວ່າໃຜເປັນຜູ້ໃຫ້ຄຳແນະນຳ ແລະ ຕິດຕາມທ່ານໃນຊ່ວງເວລາທີ່ທ່ານຖືພາຢູ່? ມີຂັ້ນຕອນແນວໃດແນ່?

<table>
<tr><td colspan="3">1. I would like you to think back to the early stages of your pregnancy. Could you please walk me through the process <u>from when you realised you were pregnant until the moment you delivered [recently pregnant] / until today [currently pregnant]</u>? Take your time to remember, I would like to go through the full process with you. ຂ້າພະເຈົ້າຢາກໃຫ້ທ່ານຄິດຍ້ອນກັບໄປໃນຊ່ວຍຂັ້ນທຳອິດຂອງການຖືພາຂອງທ່ານ. ທ່ານຊ່ວຍພາເຮົາໄປຍັງຂະບວນການຕອນເມື່ອທ່ານຮູ້ແລ້ວວ່າຖືພາຈົນເຖິງເມືອທ່ານເກີດລູກ[ຫາກໍເກີດລູກ]/ຈົນກະທັ່ງມື້ນີ້ [ກຳລັງຖືພາ]? ໃຊ້ເວລາໃນການຈື່, ຂ້າພະເຈົ້າຢາກຈະຜ່ານຊ່ວງເວລາຂອງການຖືພານີ້ໄປພ້ອມກັບທ່ານ.</td></tr>
<tr><td></td><td>Source 1 ແຫຼ່ງ 1</td><td>Source n ແຫຼ່ງ n</td></tr>
<tr><td>a) What kind of help or treatment did you get at this stage? [if unsure, specify] ການຊ່ວຍເຫຼືອ ຫຼື ການປິ່ນປົວປະເພດໃດທີ່ທ່ານໄດ້ຮັບໃນຂັ້ນນີ້? [ຖ້າບໍ່ແນ່ໃຈ, ຊ່ວຍລະບຸ]</td><td>Self-care (sleep, rest, medicine at home) ດູແລຕົວເອງ (ນອນ, ພັກຜ່ອນ, ກິນຢາຢູ່ບ້ານ) 1
Care from family and friends (full-time) ການເບິ່ງແຍງຈາກຄອບຄົວ ແລະ ໝູ່ເພື່ອນ (ເຕັມເວລາ) 2
Treated/cons. at shop / vendor selling drugs ໄດ້ຮັບການປິ່ນປົວຢູ່ຮ້ານຄ້າ/ຄົນຂາຍຢາ 3
Treated/consulted at a traditional healer ໄດ້ຮັບການປິ່ນປົວ/ປຶກສາຢູ່ໝໍພື້ນເມືອງ/ໝໍຜີ 4
Treated/cons. at a pharmacist ໄດ້ຮັບການປິ່ນປົວ/ປຶກສາກັບແພດຂາຍຢາ 5
Treated/cons. at priv. clinic/hospital ໄດ້ຮັບການປິ່ນປົວ/ປຶກສາທີ່ຄຣີນິກ/ໂຮງໝໍ 6
Treated/cons. at NGO clinic/service ໄດ້ຮັບການປິ່ນປົວ/ປຶກສາທີ່ຄຣີນິກບໍ່ສະແຫວງຫາຜົນກຳໄລ/ໂຮງໝໍ 7
Treated/cons. at health centre ໄດ້ຮັບການປິ່ນປົວ/ປຶກສາທີ່ສຸກສາລາ/ໂຮງໝໍນ້ອຍ 8
Treated/cons. at a public hospital ໄດ້ຮັບການປິ່ນປົວ/ປຶກສາທີ່ໂຮງໝໍເອກະຊົນ 9
Treated/cons. by a village health volunteer ໄດ້ຮັບການປິ່ນປົວ/ອາສາສະໝັກສາທາລະນະສຸກບ້ານ (ອສບ) 10
Treated/cons. by an unregistered/local/informal/retired doctor ໄດ້ຮັບການປິ່ນປົວ/ປຶກສາໂດຍໝໍທີ່ບໍ່ມີໃບຢັ້ງຢືນ/ທ້ອງຖິ່ນ/ບໍ່ເປັນທາງການ/ບຳນານ 11
Treated/consulted at a traditional birth assistant ໄດ້ຮັບການປິ່ນປົວ/ປຶກສາຢູ່ໝໍຕຳແຍ 15
Cross-border care/ປະເທດໃກ້ຄຽງ 97
Other (incl. internet; specify)ອື່ນໆ (ປະກອບທັງ ອິນເຕີເນັດ; ລະບຸ ___ 99</td><td>1
2
3
4
5
6
7
8
9
10
11
15
99</td></tr>
<tr><td>b) What was the purpose of getting help or treatment from this source? ຈຸດປະສົງຂອງການໄດ້ຮັບການຊ່ວຍເຫຼືອ ຫຼື ປິ່ນປົວຈາກແຫຼ່ງນັ້ນແມ່ນຫຍັງ?</td><td>Purpose ຈຸດປະສົງ: ___________________________________</td><td>___</td></tr>
</table>

c) [***if not ignore/self-care***] How did the provider talk to you?<br>[***mark all that apply***] [ຖ້າບໍ່ແມ່ນ, ຂ້າມ]<br>ຜູ້ຊ່ວຍໄດ້ເວົ້າຈາໂອ້ລົມແນວໃດກັບທ່ານ? [ໝາຍໄດ້ຫຼາຍກວ່າໜຶ່ງຂໍ້]	Supportive / understanding / praising ສະໜັບສະໜູນ/ເຂົ້າໃຈ/ຍົກຍ້ອງ 1<br>Nice / kind / friendly / positive ດີ/ໃຈດີ/ເມດຕາ/ແງ່ດີ 2<br>Neutral / descriptive / unremarkable<br>ປານກາງ/ພັນລະນາອະທິບາຍໄດ້/ບໍ່ປະທັບໃຈປານໃດ 3<br>Criticising / negative ວິຈານ/ແງ່ລົບ 4<br>Scolding / raising their voice / yelling ປ້ອຍດ່າ/ຂຶ້ນສຽງໃສ່/ຮ້ອງໃສ່ 5<br>Other (specify)ອື່ນໆ (ລະບຸ): ___________ 6<br><br>Not applicable ບໍ່ແມ່ນທັງໝົດ 8<br>Don't know / prefer not to say ບໍ່ຮູ້/ບໍ່ຢາກກ່າວ 9	1<br>2<br>3<br>4<br>5<br>6 __<br><br>8<br>9
d) [***if not ignore/self-care***] How well did you feel taken care of by the provider? [ຖ້າບໍ່ແມ່ນ, ຂ້າມ]<br>ທ່ານຮູ້ສຶກດີສໍ່າໃດສໍາລັບການເບິ່ງແຍດູແລຈາກຜູ້ຊ່ວຍນັ້ນ?	Very well ດີຫຼາຍ 1<br>Well ດີ 2<br>So-so / indifferent / no opinion ປານກາງ/ບໍ່ແຕກຕ່າງ/ບໍ່ມີຄວາມຄິດເຫັນ 3<br>Not well ບໍ່ດີ 4<br>Unacceptably badly ບໍ່ສາມາດຮັບໄດ້ 5<br><br>Not relevant ບໍ່ກ່ຽວຂ້ອງ 8<br>Prefer not to say ບໍ່ຢາກກ່າວ 9	1<br>2<br>3<br>4<br>5<br><br>8<br>9
**2.** Has any healthcare provider supported you to develop a birth plan? If so, who?<br>ມີຜູ້ສະໜອງການປິ່ນປົວຄົນໃດຊ່ວຍເຫຼືອທ່ານໃນການພັດທະນາແຜນການເກີດບໍ່? ຖ້າມີ, ແມ່ນໃຜ?	Family member ສະມາຊິກໃນຄອບຄົວ 1<br>Friend / other villager ໝູ່/ຊາວບ້ານ 2<br>Traditional healer **ໝໍພື້ນເມືອງ/ໝໍຜີ** 3<br>Pharmacist ແພດການຢາ/ຄົນຂາຍຢາ 4<br>Village health volunteer ອາສາສະໝັກສາທາລະນະສຸກບ້ານ (ອສບ) 5<br>Auxiliary nurse ຜູ້ຊ່ວຍພະຍາບານ 6<br>Nurse or midwife ພະຍາບານ ຫຼື ຜະດຸງຄັນ 7<br>Other health centre staff ເຈົ້າໜ້າທີ່ສຸກສາລາ 8<br>Medical doctor (public hospital) ແພດ/ທ່ານໝໍ (ໂຮງໝໍລັດ) 9<br>Medical doctor (private hospital) ແພດ/ທ່ານໝໍ (ໂຮງໝໍເອກະຊົນ) 10<br>Unregistered/local/informal/retired doctor 11<br>**ໝໍບໍ່ມີໃບຢັ້ງຢືນແພດ/ທ້ອງຖິ່ນ/ບໍ່ເປັນທາງການ/ໝໍບໍານານ**<br>Traditional birth assistant **ໝໍຕໍາແຍ** 15<br><br>Village chief / deputy ນາຍບ້ານ/ຮອງນາຍບ້ານ 21<br>Lao Front ແນວລາວສ້າງຊາດ 22<br>Lao Women's Union ສະຫະພັນແມ່ຍິງ 23<br>Lao Youth Union ສູນກາງຊາວໜຸ່ມ 24<br>Other authorities ແລະ ອໍານາດການປົກຄອງ ອື່ນໆ25<br>Other (specify) ອື່ນໆ (ລະບຸ): ________________________ 12<br><br>No one ບໍ່ມີບຸກຄົນອື່ນໃນບໍລິເວນສໍາພາດລະຫ່ວາງການສໍາພາດ 98<br>Don't know / prefer not to say ບໍ່ຮູ້ / ບໍ່ຢາກກ່າວ99	
**3.** [***Currently pregnant women***] Where do you plan to give birth? [ຄັ້ງທີ1 & 2: ແມ່ຍິງທີ່ກໍາລັງຖືພາ]<br>ທ່ານວາງແຜນທີ່ຈະເກີດລູກຢູ່ໃສ?<br>[***Recently pregnant women***] Where did you give birth?<br>[ແມ່ຍິງທີ່ເກີດລູກເມື່ອບໍ່ດົນມານີ້] ທ່ານໄດ້ເກີດລູກຢູ່ສະຖານທີ່ໃດ?	Public hospital ໂຮງໝໍລັດ 1<br>Health centre ສຸກສາລາ/ໂຮງໝໍນ້ອຍ 2<br>Private clinic ຄຣີນິກເອກະຊົນ 3<br>Own home ບ້ານຂອງຕົນເອງ 4<br>Other home ບ້ານອື່ນ 5<br>Cross-border care/ປະເທດໃກ້ຄຽງ 97<br>Other ອື່ນໆ(specify) ລະບຸ: _______________ 98<br>Don't know / prefer not to say ບໍ່ຮູ້ / ບໍ່ຢາກກ່າວ99	

**3.1.** If at home, who would you expect to be / was present to help? [***mark all that apply***]<br>ຖ້າຢູ່ບ້ານຂອງຕົນເອງ, ທ່ານຄາດຫວັງໃຫ້ໃຜເປັນ/ຫຼືຕາງໜ້າໃນການທີ່ຈະຊ່ວຍທ່ານເກີດລູກ? [ທ່ານສາມາດເລືອກໄດ້ຫຼາຍກວ່າ 1 ຂໍ້]	Family member ສະມາຊິກໃນຄອບຄົວ 1<br>Friend / other villager ໝູ່/ຊາວບ້ານ 2<br>Traditional healer **ໝໍພື້ນເມືອງ/ໝໍຜີ** 3<br>Pharmacist ແພດການຢາ/ຄົນຂາຍຢາ 4<br>Health volunteer ແພດອາສາ 5<br>Auxiliary nurse ຜູ້ຊ່ວຍພະຍາບານ 6<br>Nurse or midwife ພະຍາບານ ຫຼື ຜະດຸງຄັນ 7<br>Other health centre staff ເຈົ້າໜ້າທີ່ສຸກສາລາ 8<br>Medical doctor (public hospital) ແພດ/ທ່ານໝໍ (ໂຮງໝໍລັດ) 9<br>Medical doctor (private hospital) ແພດ/ທ່ານໝໍ (ໂຮງໝໍເອກະຊົນ) 10<br>Unregistered/local/informal/retired doctor 11<br>**ໝໍບໍ່ມີໃບຢັ້ງຢືນແພດ/ທ້ອງຖິ່ນ/ບໍ່ເປັນທາງການ/ໝໍບໍານານ**<br>Traditional birth assistant **ໝໍຕໍາແຍ** 15<br>Other (specify) ອື່ນໆ (ລະບຸ): ________________________ 12<br>Don't know / prefer not to say ບໍ່ຮູ້ / ບໍ່ຢາກກ່າວ99
**3.2.** What is/was the main reason for your choice?<br>ສິ່ງໃດເປັນເຫດຜົນຫຼັກໃນການເລືອກຂອງທ່ານ?	Reason ເຫດຜົນ: _______________________
**3.3.** Has anyone encouraged you to give birth at that place?<br>ມີໃຜຊຸກຍູ້ໃຫ້ທ່ານເກີດລູກຢູ່ສະຖານທີ່ນັ້ນບໍ່?	Family member ສະມາຊິກໃນຄອບຄົວ 1<br>Friend / other villager ໝູ່/ຊາວບ້ານ 2<br>Traditional healer **ໝໍພື້ນເມືອງ/ໝໍຜີ** 3<br>Pharmacist ແພດການຢາ/ຄົນຂາຍຢາ 4<br>Village health volunteer ອາສາສະໝັກສາທາລະນະສຸກບ້ານ (ອສບ) 5<br>Auxiliary nurse ຜູ້ຊ່ວຍພະຍາບານ 6<br>Nurse or midwife ພະຍາບານ ຫຼື ຜະດຸງຄັນ 7<br>Other health centre staff ເຈົ້າໜ້າທີ່ສຸກສາລາ 8<br>Medical doctor (public hospital) ແພດ/ທ່ານໝໍ (ໂຮງໝໍລັດ) 9<br>Medical doctor (private hospital) ແພດ/ທ່ານໝໍ (ໂຮງໝໍເອກະຊົນ) 10<br>Unregistered/local/informal/retired doctor 11<br>**ໝໍບໍ່ມີໃບຢັ້ງຢືນແພດ/ທ້ອງຖິ່ນ/ບໍ່ເປັນທາງການ/ໝໍບໍານານ**<br>Traditional birth assistant **ໝໍຕໍາແຍ** 15<br><br>Village chief / deputy ນາຍບ້ານ/ຮອງນາຍບ້ານ 21<br>Lao Front ແນວລາວສ້າງຊາດ 22<br>Lao Women's Union ສະຫະພັນແມ່ຍິງ 23<br>Lao Youth Union ຊາວໜຸ່ມ 24<br>Other authorities ແລະ ອໍານາດການປົກຄອງ ອື່ນໆ25<br>Other (specify) ອື່ນໆ (ລະບຸ): ________________________ 12<br><br>No one ບໍ່ມີບຸກຄົນອື່ນໃນບໍລິເວນສໍາພາດລະຫ່ວາງການສໍາພາດ 98<br>Don't know / prefer not to say ບໍ່ຮູ້ / ບໍ່ຢາກກ່າວ99

**3.4.** [***if Question 3 did not include "health centre"***] Has anyone encouraged you to give birth specifically at the health centre?<br>[**ຖ້າ 3 ບໍ່ໄດ້ມີ "ສຸກສາລາ"**]<br>ມີໃຜຊຸກຍູ້ໃຫ້ທ່ານເກີດລູກຢູ່ສຸກສາລາໂດຍສະເພາະບໍ່?	Family member ສະມາຊິກໃນຄອບຄົວ 1<br>Friend / other villager ໝູ່/ຊາວບ້ານ 2<br>Traditional healer **ໝໍພື້ນເມືອງ/ໝໍຜີ** 3<br>Pharmacist ແພດການຢາ/ຄົນຂາຍຢາ 4<br>Village health volunteer ອາສາສະໝັກສາທາລະນະສຸກບ້ານ (ອສບ) 5<br>Auxiliary nurse ຜູ້ຊ່ວຍພະຍາບານ 6<br>Nurse or midwife ພະຍາບານ ຫຼື ຜະດຸງຄັນ 7<br>Other health centre staff ເຈົ້າໜ້າທີ່ສຸກສາລາ 8<br>Medical doctor (public hospital) ແພດ/ທ່ານໝໍ (ໂຮງໝໍລັດ) 9<br>Medical doctor (private hospital) ແພດ/ທ່ານໝໍ (ໂຮງໝໍເອກະຊົນ) 10<br>Unregistered/local/informal/retired doctor 11<br>**ໝໍບໍ່ມີໃບຢັ້ງຢືນແພດ/ທ້ອງຖິ່ນ/ບໍ່ເປັນທາງການ/ໝໍບໍານານ**<br>Traditional birth assistant **ໝໍຕໍາແຍ** 15<br><br>Village chief / deputy ນາຍບ້ານ/ຮອງນາຍບ້ານ 21<br>Lao Front ແນວລາວສ້າງຊາດ 22<br>Lao Women's Union ສະຫະພັນແມ່ຍິງ 23<br>Lao Youth Union ສູນກາງຊາວໜຸ່ມ 24<br>Other authorities ແລະ ອໍານາດການປົກຄອງ ອື່ນໆ25<br>Other (specify) ອື່ນໆ (ລະບຸ): ________________________ 12<br><br>No one ບໍ່ມີບຸກຄົນອື່ນໃນບໍລິເວນສໍາພາດລະຫ່ວາງການສໍາພາດ 98<br>Don't know / prefer not to say ບໍ່ຮູ້ / ບໍ່ຢາກກ່າວ99
**4.** [***for recently pregnant women***] Can you describe the experience of giving birth at this place? How was it going into labour, did you feel safe?<br>[ແມ່ຍິງທີ່ເກີດລູກເມື່ອບໍ່ດົນມານີ້]<br>ຊ່ວຍໃຫ້ທ່ານອະທິບາຍປະສົບການໃນການເກີດຂຶ້ນຢູ່ສະຖານທີ່ນີ້ໄດ້ບໍ່? ຕອນກໍາລັງເກີດລູກເປັນແນວໃດ? ທ່ານຮູ້ສຶກປອດໄພບໍ່?	Explanation ອະທິບາຍ: ______________________________

[*after M2 workshop has been delivered*] A 2-day workshop took place recently (three months ago) in your village, involving healthcare workers and villagers. Are you aware of this workshop? [provide slight help if respondent is not entirely sure] ເວີກຊ໋ອບ 2 ມື້ ເກີດຂຶ້ນ ສາມເດືອນກ່ອນໜ້ານີ້ໃນໝູ່ບ້ານຂອງທ່ານ, ມີສ່ວນຮ່ວມໂດຍແພດໝໍ ແລະ ຊາວບ້ານ. ທ່ານຮູ້ຈັກເວີກຊ໋ອບນີ້ບໍ່? [ຊ່ວຍຜູ້ຕອບແບບສອບຖາມເມື່ອບໍ່ແນ່ໃຈ] If so, I would like to ask you a few final questions.

[Note to interviewer: discuss each question to see how participants understands it and what their answer means – probes are below. Use these quotes to stimulate the conversation while going through the survey items in the table below.] [ໝາຍເຫດເຖິງຜູ້ສໍາພາດ: ສົນທະນາແຕ່ລະຄໍາຖາມເພື່ອສັງເກດເບິ່ງວ່າຜູ້ເຂົ້າຮ່ວມເຂົ້າໃຈແນວໃດ ແລະ ຄໍາຕອບຂອງພວກເຂົາໝາຍຄວາມວ່າແນວໃດ-ການຖາມຕອບດ້ານລຸ່ມນີ້. ໃຊ້ຄໍາເວົ້າເຫຼົ່ານີ້ເພື່ອກະຕຸ້ນການສົນທະນາ ໃນຂະນະທີ່ເຮັດແບບສໍາຫຼວດຕາມຕາຕະລາງໃນດ້ານລຸ່ມນີ້.]

a. Can you please explain your answers to me? ທ່ານສາມາດອະທິບາຍຄໍາຕອບຂອງທ່ານໃຫ້ເຮົາຟັງໄດ້ບໍ່?
b. Were there questions that you found difficult or not really appropriate? ທ່ານພົບວ່າຄໍາຖາມເຫຼົ່ານັ້ນມີຄວາມຍາກ - ບໍ່ເໝາະສົມ ຫຼື ບໍ່?
c. Did you think about specific situations when answering the question? What did you relate to? ທ່ານໄດ້ຄິດເຖິງສະຖານະການໃດໜຶ່ງເປັນການສະເພາະ ຫຼື ບໍ່ ໃນຂະນະທີ່ທ່ານຕອບຄໍາຖາມ? ທ່ານຄິດເຫັນເຫດການໃດ?
d. Can you explain your experience and expectations of the workshop? ທ່ານສາມາດອະທິບາຍໄດ້ບໍ່ວ່າປະສົບການ ແລະ ຄວາມຄາດຫວັງຂອງທ່ານຕໍ່ກອງປະຊຸມນີ້ແນວໃດ?
e. Have you seen anything in the village change as a result of the workshop? What was that? ທ່ານໄດ້ເຫັນການປ່ຽນແປງພາຍໃນບ້ານບໍ່ ພາຍຫຼັງກອງປະຊຸມນີ້ສໍາເລັດແລ້ວ? ການປ່ຽນແປງທີ່ວ່ານັ້ນແມ່ນຫຍັງ?
f. Do you think that any such changes could last? ທ່ານຄິດວ່າການປ່ຽນແປງດັ່ງກ່າວຈະຍືນຍົງບໍ່?

**1.**	Were you invited to participate in the workshop?<br>ທ່ານໄດ້ຖືກເຊີນໃຫ້ເຂົ້າຮ່ວມໃນງານເວີກຊ໋ອບ ຫຼື ບໍ່?	Yes ແມ່ນ 1<br>No ບໍ່ແມ່ນ 0<br>Don't know / prefer not to say ບໍ່ຮູ້ / ບໍ່ຢາກກ່າວ 9
**2.**	Did you attend the activities?<br>ທ່ານໄດ້ເຂົ້າຮ່ວມການເຮັດກິດຈະກຳເຫຼົ່ານັ້ນ ຫຼື ບໍ່?	Yes ແມ່ນ 2<br>Yes, but not throughout ເຂົ້າຮ່ວມ, ແຕ່ບໍ່ໄດ້ເຂົ້າຮ່ວມທຸກກິດຈະກຳ 1<br>No ບໍ່ແມ່ນ 0<br>Don't know / prefer not to say ບໍ່ຮູ້ / ບໍ່ຢາກກ່າວ 9
**3.**	[*if yes / yes but to Question 2*] Did you feel included in the activity?<br>[ຖ້າທ່ານຕອບ ແມ່ນ ແຕ່ໃນຂໍ້ 2] ທ່ານຮູ້ສຶກມີສ່ວນຮ່ວມໃນກິດຈະກຳນັ້ນ ຫຼື ບໍ່?	Yes ແມ່ນ 1<br>No ບໍ່ແມ່ນ 0<br>Don't know / prefer not to say ບໍ່ຮູ້ / ບໍ່ຢາກກ່າວ 9
**4.**	[*if yes / yes but to Question 2*] Did you have the chance to contribute to the conversations? [ຖ້າທ່ານຕອບ ແມ່ນ ແຕ່ໃນຂໍ້ 2]<br>ທ່ານໄດ້ມີໂອກາດປະກອບສ່ວນໃນການສົນທະນາເຫຼົ່ານັ້ນ ຫຼື ບໍ່?	Yes ແມ່ນ 1<br>No ບໍ່ແມ່ນ 0<br>Don't know / prefer not to say ບໍ່ຮູ້ / ບໍ່ຢາກກ່າວ 9
**5.**	[*if yes / yes but to Question 2*] Did you feel that you were involved in the decisions of the workshop?<br>[ຖ້າທ່ານຕອບ ແມ່ນ ແຕ່ໃນຂໍ້ 2]<br>ທ່ານຮູ້ສຶກວ່າທ່ານໄດ້ມີສ່ວນຮ່ວມໃນການຕັດສິນໃຈ ໃນງານເວີກຊ໋ອບນັ້ນ ຫຼື ບໍ່?	Yes ແມ່ນ 1<br>No ບໍ່ແມ່ນ 0<br>Don't know / prefer not to say ບໍ່ຮູ້ / ບໍ່ຢາກກ່າວ 9
**6.**	What, in your opinion, was the activity about?<br>ໃນຄວາມຄິດເຫັນຂອງທ່ານ, ທ່ານຄິດວ່າກິດຈະກຳນີ້ແມ່ນຈັດກ່ຽວກັບຫຍັງ?	Response ຕອບ: ________________________________
**7.**	Have you heard about the orange tree, decisions, or an action plan? [*mark all that apply*]<br>ທ່ານເຄີຍໄດ້ຍິນກ່ຽວກັບຕົ້ນໄມ້ຈຸດປະສົງ, ການຕັດສິນໃຈ, ຫຼື ແຜນວຽກຕົວຈິງບໍ່?<br>[ທ່ານສາມາດເລືອກໄດ້ຫຼາຍກວ່າ 1 ຂໍ້]	Have not heard about it ບໍ່ເຄີຍໄດ້ຍິນມາກ່ອນ 0<br>Orange tree ຕົ້ນໄມ້ຈຸດປະສົງ/ oranges ຈຸດປະສົງ 1<br>Activity decisions ການຕັດສິນໃຈໃນກິດຈະກຳ 2<br>Action plan ແຜນວຽກຕົວຈິງ 3<br>Other description of action plan ຄວາມໝາຍອື່ນໆ ຂອງ ແຜນວຽກຕົວຈິງ 4<br>Don't know / prefer not to say ບໍ່ຮູ້ / ບໍ່ຢາກກ່າວ 9
**8.**	[*if Question 7 is 1-4*] What, in your opinion, does the orange tree/decisions / action plan say? [ຖ້າ ຂໍ້ 7 ແມ່ນ ຕົວເລືອກ 1-4]<br>ໃນຄວາມຄິດເຫັນຂອງທ່ານ ຕົ້ນໄມ້ຈຸດປະສົງ/ການຕັດສິນໃຈ/ ແຜນການປະຕິບັດວຽກຕົວຈິງ ມີຄວາມໝາຍວ່າແນວໃດ?	Response ຄຳຕອບ: ________________________________
**9.**	[*if Question 7 is 1-4*] Have you seen any of these actions being implemented? [ຖ້າ ຂໍ້ 7 ແມ່ນ ຕົວເລືອກ 1-4]<br>ທ່ານເຄີຍພົບເຫັນກິດຈະກຳໃດໜຶ່ງນີ້ຖືກນຳໄປປະຕິບັດຕົວຈິງບໍ່?	Yes ແມ່ນ 1<br>No ບໍ່ແມ່ນ 0<br>Don't know / prefer not to say ບໍ່ຮູ້ / ບໍ່ຢາກກ່າວ 9
**10.**	In the earlier question, you mentioned that healthcare services had [*improved/not changed/worsened*] in the last three months. Do you think that the workshop activities were responsible for that?<br>ໃນຄຳຖາມ ກ່ອນໜ້ານີ້, ທ່ານໄດ້ກ່າວເຖິງການບໍລິການຂອງສຸກສາລາ/ ໂຮງໝໍນ້ອຍໄດ້ [ມີການພັດທະນາຂຶ້ນ/ ບໍ່ມີການປ່ຽນແປງ/ ບໍ່ດີກ່ອນເກົ່າ] ໃນໄລຍະເວລາ 3 ເດືອນທີ່ຜ່ານມາ.<br>ທ່ານຄິດວ່າກິດຈະກຳຂອງງານເວີກຊ໋ອບເຫຼົ່ານີ້ໄດ້ມີຜົນກະທົບຫຍັງບໍ່ຕໍ່ຂໍ້ນັ້ນ?	Yes ແມ່ນ 1<br>No ບໍ່ແມ່ນ 0<br>Don't know / prefer not to say ບໍ່ຮູ້ / ບໍ່ຢາກກ່າວ 9

This is the end of our interview. Thank you so much for sharing your views with me. Your responses have been very helpful. ການສຳພາດນີ້ສິ້ນສຸດແລ້ວ ຄຳຕອບທີ່ທ່ານໃຫ້ກັບເຮົາຈະເປັນປະໂຫຍດກັບການຄົ້ນຄ້ວາຂອງເຮົາຫຼາຍ ຂອບໃຈສຳລັບຄຳຄິດເຫັນທີ່ທ່ານແບ່ງປັນກັບເຮົາ

INTERVIEW GUIDES – KEY INFORMANT INTERVIEWS
ບົດສໍາພາດ - ບົດສໍາພາດຂອງຜູ້ໃຫ້ຂໍ້ມູນ

Overview

The Goal of CONNECT is to "Enhance local health governance for Primary Health Care (PHC) beyond COVID-19 in alignment with Samsang to achieve national assembly health goals, Universal Health Coverage and Sustainable Development Goals." This interview guide is directed at stakeholders of CONNECT to help inform the evaluation of the initiative with insights from the community to the provincial and central levels.

ເປົ້າໝາຍຂອງ CONNECT ແມ່ນເພື່ອ "ເສີມຂະຫຍາຍການຄຸ້ມຄອງສຸຂະພາບຂັ້ນທ້ອງຖິ່ນ ສໍາລັບການເບິ່ງແຍງສຸຂະພາບໃນເບື້ອງຕົ້ນ (PHC) ນອກເໜືອຈາກ COVID-19 ທີ່ສອດຄ່ອງກັບ 3 ສ້າງ ເພື່ອບັນລຸເປົ້າໝາຍດ້ານສຸຂະພາບຂອງສະພາແຫ່ງຊາດ, ການຄຸ້ມຄອງສຸຂະພາບທົ່ວໄປ ແລະ ເປົ້າໝາຍການພັດທະນາແບບຍືນຍົງ." ຄູ່ມືໃນການສໍາພາດນີ້ແມ່ນມຸ່ງໄປຫາຜູ້ທີ່ມີສ່ວນຮ່ວມໃນໂຄງການຂອງ CONNECT ເພື່ອຊ່ວຍໃນການແຈ້ງໃຫ້ຜູ້ປະເມີນ

Objectives

- Inform the evaluation of CONNECT initiative along the evaluation criteria ແຈ້ງຜົນການປະເມີນໂຄງການຂອງ CONNECT ໂດຍອີງຕາມເກນຂອງການປະເມີນ
- Gain an understanding of the community-level behavioural mechanisms of impact ທໍາຄວາມເຂົ້າໃຈກັບໄກພຶດຕິກໍາຂອງຜົນກະທົບໃນຊຸມຊົນ

Notes

- Total duration of each interview to be 40-60 minutes, up to 50 interviews. ໄລຍະເວລາໃນການສໍາພາດແຕ່ລະຄັ້ງຈະໃຊ້ເວລາປະມານ 40 - 60 ນາທີ, ປະມານ 50 ຄັ້ງໃນການສໍາພາດ
- The interview format is semi-structured, meaning that it is a conversation around the survey questions. Probes are provided to guide the conversation. ຮູບແບບໃນການສໍາພາດແມ່ນ ແບບເຄິ່ງໂຄງສ້າງ, ເຊິ່ງໝາຍຄວາມວ່າມັນເປັນການສົນທະນາ
- Different community groups will be recruited into the interviews (i.e. the interview team should find those groups specifically), ideally after relevant M2 and M3 workshops have been implemented: ກຸ່ມຊຸມຊົນຕ່າງໆຈະຖືກຄັດເລືອກເຂົ້າຮ່ວມໃນການສໍາພາດ (ເຊັ່ນ: ທີມສໍາພາດຄວນຄົ້ນຫາກຸ່ມເຫຼົ່ານັ້ນໂດຍສະເພາະ) ໂດຍສະເພາະພາຍຫຼັງເວີກຊ໋ອບ M2 ແລະ M3 ທີ່ກ່ຽວຂ້ອງແລ້ວ.
    - Workshop facilitator / CONNECT team (approx. 5) ຜູ້ຈັດເວີກຊ໋ອບ/ທີມງານ CONNECT (ປະມານ 5ຄົນ)
    - Village representative (5-10; 1-2 per district) ຕົວແທນບ້ານ (5-10ຄົນ; 1-2 ຄົນ ຕໍ່ເມືອງ)
    - Village leader (approx. 5) ນາຍບ້ານ (ປະມານ 5ຄົນ)
    - Healthcare staff (5-10; 1-2 per district) ພະນັກງານແພດ (5-10 ຄົນ; 1-2 ຄົນ ຕໍ່ເມືອງ)
    - Local & district government stakeholder (5-10; 1-2 per district) ພາກສ່ວນທີ່ກ່ຽວຂ້ອງທາງພາກລັດທັງຂັ້ນເມືອງ ແລະ ຂັ້ນທ້ອງຖິ່ນ (5-10 ຄົນ; 1-2 ຄົນ ຕໍ່ເມືອງ)
    - Provincial & central government stakeholder (5-10) ພາກສ່ວນກ່ຽວຂ້ອງຂັ້ນແຂວງ ແລະ ຂັ້ນສູນກາງ (5-10 ຄົນ)
    - Other stakeholder groups (5-10; e.g. trans rights groups in Vientiane) ພາກສ່ວນອື່ນໆທີ່ກ່ຽວຂ້ອງ
- As a semi-structured interview, the interviewer can choose to focus the conversation on key topics of interest (as long as it supports the objective and if other interviews complement the missing perspectives). ເປັນການສໍາພາດແບບເຄິ່ງໂຄງສ້າງ , ຜູ້ສໍາພາດສາມາດເລືອກທີ່ຈະສຸມໃສ່ການ

ສິນທະນາໃນຫົວຂໍ້ທີ່ສໍາຄັນທີ່ໃຫ້ຄວາມສົນໃຈ (ຖ້າຫາກມັນໄດ້ສະໜັບສະໜູນຈຸດປະສົງ ແລະ ການສໍາພາດອື່ນໆທີ່ຈະມາເສີມໃນສ່ວນຂອງມຸມມອງ/ທັດສະນະຄະຕິທີ່ຂາດໄປ)

- The table below lists all topics ("themes") and the items to inform these topics. Not all sections and items are relevant for everyone – the respondent type information indicates which topics can be skipped. ຕາຕະລາງດ້ານລຸ່ມນີ້ໄດ້ສະແດງຫົວຂໍ້ທັງໝົດ (ຫົວຂໍ້) ແລະ ລາຍການຍ່ອຍທີ່ຈະແຈ້ງໃນບັນດາຫົວຂໍ້ເຫຼົ່ານີ້. ຫົວຂໍ້ ແລະ ລາຍການບາງອັນອາດບໍ່ໄດ້ກ່ຽວຂ້ອງກັບທຸກໆຄົນ - ປະເພດຂໍ້ມູນຂອງຜູ້ຕອບຈະສາມາດບອກໄດ້ວ່າຫົວໃດສາມາດຂ້າມໄປໄດ້.

Introduction ບົດນໍາ

<Obtaining verbal informed consent in accordance with Oral Consent Script> <ດໍາເນີນການຂໍຄໍາຍິນຍອມໂດຍໃຊ້ຂໍ້ຄວາມສໍາລັບອະທິບາຍວຽກຄົ້ນຄ້ວາແລະຂໍຄໍາຍິນຍອມເຂົ້າຮ່ວມການຄົ້ນຄ້ວາດ້ວຍວາຈາ>

Thank you for agreeing to participate in this study. ຂອບໃຈທ່ານສໍາລັບການຕົກລົງເຂົ້າຮ່ວມການຄົ້ນຄ້ວາໃນຄັ້ງນີ້

0. Let us start with a few questions about yourself. ເຮົາຈະເລີ່ມໂດຍຖາມຄໍາຖາມກ່ຽວກັບຂໍ້ມູນສ່ວນຕົວຂອງທ່ານ
   a) How old are you? ອາຍຸທ່ານ
   b) [*record as observed*] Sex [ບັນທຶກຈາກວິຈາລະນະຍານຂອງຜູ້ສໍາພາດ] ເພດ
   c) What is your ethnicity? ເຊື້ອຊາດ
   d) What is your occupation / position / role, and how long have you had your current position [if not community member] ອາຊີບ/ຕໍາແໜ່ງ/ໜ້າທີ່ຂອງທ່ານແມ່ນຫຍັງ? ແລະ ທ່ານໄດ້ເຮັດວຽກໃນຕໍາແໜ່ງນີ້ດົນປານໃດແລ້ວ? [ຖ້າທ່ານບໍ່ໄດ້ເປັນສະມາຊິກພາຍໃນຊຸມຊົນ]
   e) How would you describe your relationship to the CONNECT initiative? [e.g. participant in the workshops, working on health policy, interested in inclusive development, delivering healthcare services] ທ່ານຈະອະທິບາຍ
   f) When have you first learned about CONNECT? Can you summarise how you understand the initiative from your point of view? ທ່ານໄດ້ຮຽນຮູ້ກ່ຽວກັບໂຄງການ CONNECT ເປັນຄັ້ງທໍາອິດ

[*Note for interviewer: Now continue following the topic guide below. Probes and directions for guiding questions are indicated as sub-themes. Section 8 is lower priority and can be covered after all other themes have been informed sufficiently. See CONNECT Evaluation Concept Note for explanation of the key themes.*] [ໝາຍເຫດສໍາລັບຜູ້ສໍາພາດ: ປະຕິບັດຕາມຫົວຂໍ້ແນະນໍາດ້ານລຸ່ມນີ້. ການຖາມ-ຕອບ ແລະ ຄໍາຖາມແນະນໍາສໍາລັບຄໍາຖາມແນະນໍາຈະສະແດງເປັນຫົວຂໍ້ຍ່ອຍ. ສ່ວນ ຫົວຂໍ້ທີ 8 ມີລໍາດັບຄວາມສໍາຄັນໜ້ອຍກວ່າ ແລະ ສາມາດຄອບຄຸມໄດ້ພາຍຫຼັງຫົວຂໍ້ອື່ນໆທັງໝົດໄດ້ຖືກລາຍງານຢ່າງຄົບຖ້ວນ. ສັງເກດໝາຍເຫດແນວຄວາມຄິດໃນການປະເມີນຂອງ CONNECT ສໍາລັບຄໍາອະທິບາຍຂອງຫົວຂໍ້ຫຼັກ]

Theme and sub-theme<br>**ຫົວຂໍ້ ແລະ ຫົວຂໍ້ຍ່ອຍ**	Respondent type<br>**ປະເພດຂອງຜູ້ຕອບແບບສອບຖາມ**						
	Workshop facilitator / CONNECT team	Village representative	Village leader	Healthcare staff	Local & district govern. stakeholder	Provincial & central governm. stakeholder	Other stakeholder groups
1. Relevance **ຄວາມກ່ຽວຂ້ອງ**<br>*Guiding question: Does CONNECT respond to the needs, priorities, and policies of the target group and stakeholders – both in practice and in theory? ຄຳຖາມແນະນຳ: CONNECT ໄດ້ຕອບສະໜອງຄວາມຕ້ອງການ, ບູລິມະສິດ, ນະໂຍບາຍຂອງກຸ່ມເປົ້າໝາຍ ແລະ ຜູ້ມີສ່ວນຮ່ວມ ຫຼື ບໍ່? ທັງໃນທາງປະຕິບັດ ແລະ ທາງທິດສະດີ?*							
a. Governance context prior to CONNECT (e.g. levels of funding mobilization, participatory and community-led approaches) ບົດບາດໃນການຄຸ້ມຄອງກ່ອນ CONNECT (ເຊັ່ນ: ລະດັບຂັ້ນຂອງການລະດົມທຶນ, ແນວທາງໃນການມີສ່ວນຮ່ວມ ແລະ ການນຳໂດຍຊຸມຊົນ)					X	X	X
b. Village context prior to CONNECT implementation (e.g. community priorities, expectations about development and CONNECT) ສະພາບການຂອງໝູ່ບ້ານກ່ອນເຂົ້າຮ່ວມໂຄງການ CONNECT (ເຊັ່ນ: ລຳດັບຄວາມສຳຄັນຂອງຊຸມຊົນ, ຄວາມຄາດຫວັງກ່ຽວກັບການພັດທະນາ ແລະ ໂຄງການ CONNECT)		X	X	X			X
c. Examples of health service provision that has / has not worked well before CONNECT ຕົວຢ່າງຂອງການບໍລິການທີ່ບໍລິການໄດ້ດີ ແລະ ບໍ່ດີ ບານໃດກ່ອນທີ່ຈະມີໂຄງການ CONNECT	X	X	X	X	X	X	X
d. Examples of well-working health policies, and why they worked well ຕົວຢ່າງຂອງນະໂຍບາຍກ່ຽວກັບສຸຂະພາບທີ່ປະຕິບັດໄດ້ດີ, ແລະ ຍ້ອນຫຍັງຈຶ່ງສາມາດປະຕິບັດໄດ້ດີ?	X	X	X	X	X	X	X
e. People's general trust in health services before CONNECT ຄວາມໄວ້ເນື້ອເຊື່ອໃຈທົ່ວໄປຂອງປະຊາຊົນ ໃນການບໍລິການປິ່ນປົວສຸຂະພາບກ່ອນໂຄງການ CONNECT	X	X	X	X	X	X	X
f. Ability of the CONNECT initiative to overcome priority challenges ຄວາມສາມາດໃນການລິເລີ່ມຂອງໂຄງການ CONNECT ເພື່ອເອົາຊະນະຄວາມທ້າທາຍທີ່ມີການລຳດັບຄວາມສຳຄັນ	X	X	X	X	X	X	X
2. Coherence **ຄວາມສອດຄ່ອງ**<br>*Guiding question: How well does CONNECT fit in the country/sector/institution, existing strategies, the general configuration of behaviors and solutions shaping current practice, and the broader health system? ຄຳຖາມແນະນຳ: ໂຄງການ CONNECT ເໝາະກັບ ປະເທດ/ຂະແໜງການ/ສະຖາບັນ/ຍຸດທະສາດທີ່ມີຢູ່, ການກຳນົດທົ່ວໄປຂອງພຶດຕິກຳ ແລະ ວິທີແກ້ໄຂບັນຫາທີ່ກຳນົດແນວທາງໃນການປະຕິບັດ ແລະ ລະບົບສາທາລະນະສຸກທີ່ກວ້າງຂຶ້ນ ຫຼື ບໍ່?*							
a. CONNECT's ability to forge links between government agencies ສັກກະຍະພາບຄວາມສາມາດຂອງໂຄງການ CONNECT ໃນການສ້າງຄວາມເຊື່ອມໂຍງລະຫວ່າງອົງການລັດຖະບານ			X	X	X	X	X
b. Integration of CONNECT into government systems (decision-making, information, communication) ການເຊື່ອມໂຍງ CONNECT ເຂົ້າກັບລະບົບຂອງລັດຖະບານ (ການຕັດສິນໃຈ, ຂໍ້ມູນຂ່າວສານ, ການສື່ສານ)			X	X	X	X	
c. Integration of CONNECT into existing (COVID and MCH) health policies ການເຊື່ອມໂຍງໂຄງການ CONNECT ເຂົ້າໄປກັບນະໂຍບາຍສາທາລະນະສຸກທີ່ມີຢູ່ແລ້ວ (COVID ແລະ MCH).				X	X	X	
d. Integration of CONNECT into community health practices ການເຊື່ອມໂຍງໂຄງການ CONNECT ເຂົ້າກັບການປະຕິບັດດ້ານສຸຂະພາບ/ສາທາລະນະສຸກຂອງຊຸມຊົນ		X	X	X			

Theme and sub-theme<br>ຫົວຂໍ້ ແລະ ຫົວຂໍ້ຍ່ອຍ	Respondent type<br>ປະເພດຂອງຜູ້ຕອບແບບສອບຖາມ<br>Workshop facilitator / CONNECT team	Village representative	Village leader	Healthcare staff	Local & district govern. stakeholder	Provincial & central governm. stakeholder	Other stakeholder groups
e. Evidence that government policy learns from CONNECT's lessons ຫຼັກຖານອ້າງອີງທີ່ສະແດງວ່ານະໂຍບາຍຂອງລັດຖະບານໄດ້ຮຽນຮູ້ເພີ່ມເຕີມຈາກບົດຮຽນຂອງໂຄງການ CONNECT				X	X	X	
3. Effectiveness **ປະສິດທິຜົນ**<br>*Guiding question: Does CONNECT achieve its self-set objectives? ໂຄງການ CONNECT ໄດ້ບັນລຸຕາມເປົ້າໝາຍທີ່ວາງໄວ້ບໍ່?*							
a. Mobilisation of funding (e.g. more funding, re-direction of funds to health sector) ການລະດົມທຶນ (ເຊັ່ນ: ການລະດົມທຶນເພີ່ມເຕີມ, ການຫັນປ່ຽນທິດທາງຂອງທຶນຄືນໃໝ່ໃຫ້ແກ່ຂະແໜງສາທາລະນະສຸກ)			X	X	X	X	
b. Adaptation of government operations and cross-sectoral cooperation (structures, mechanisms, performance) ການປັບປຸງການເຄື່ອນໄຫວຂອງລັດ ແລະ ການຮ່ວມມືລະຫວ່າງຂະແໜງການທີ່ກ່ຽວຂ້ອງ (ໂຄງສ້າງ, ຮູບແບບແລະ ການປະຕິບັດ)					X	X	
c. Review of notions/indicators of "successful society" ການທົບທວນຄືນແນວຄິດ / ຕົວຊີ້ວັດຂອງ "ສັງຄົມທີ່ປະສົບຜົນສໍາເລັດ"		X	X	X	X	X	
d. Increased recognition of community-led activities for health equity ການຮັບຮູ້ເພີ່ມຂຶ້ນຂອງກິດຈະກໍາທີ່ນໍາພາໂດຍຊຸມຊົນເພື່ອຄວາມເທົ່າທຽມດ້ານສຸຂະພາບ/ສາທາລະນະສຸກ			X	X	X	X	
e. Development of sense of community ownership of health(care) ການພັດທະນາຄວາມຮູ້ສຶກຄວາມເປັນເຈົ້າຂອງດ້ານສຸຂະພາບຂອງຊຸມຊົນ (ການເບິ່ງແຍງ)	X	X	X	X			
f. Changing relationships between villagers and health center staff ການປ່ຽນແປງການພົວພັນລະຫວ່າງປະຊາຊົນໃນຊຸມຊົນ ແລະ ພະນັກງານແພດ	X	X	X	X	X	X	
g. Evolving provision and reception of maternal healthcare services ການພັດທະນາການໃຫ້ບໍລິການ ແລະ ການຮັບບໍລິການດ້ານການຮັກສາສຸຂະພາບຂອງແມ່		X	X	X	X	X	
h. Evolving COVID-19 response and reception ການພັດທະນາການຕອບສະໜອງ ແລະ ການຮັບມືກັບ COVID-19		X	X	X	X	X	
i. Vaccination ການສັກຢາວັກຊີນ							
ii. Testing ການກວດຫາເຊື້ອ							
iii. Family support for those in quarantine and isolation ການຊ່ວຍເຫຼືອຈາກຄອບຄົວສໍາລັບຜູ້ທີ່ຢູ່ໃນຊ່ວງການກັກຕົວ ແລະ ການແຍກປ່ຽວ							
iv. Enablement of home care and care pathways for isolating / quarantining households ການສ້າງແນວທາງໃນການເບິ່ງແຍງບ້ານ ແລະ ການເບິ່ງແຍງສໍາລັບການແຍກປ່ຽວ/ການກັກຕົວຢູ່ບ້ານ							
v. Stigma prevention ການປ້ອງກັນການໃສ່ຮ້າຍປ້າຍສີ							
i. Nature of trust and its changes after workshop ລັກສະນະຂອງຄວາມໄວ້ວາງໃຈ ແລະ ການປ່ຽນແປງພາຍຫຼັງເວີກຊັອບ	X	X	X	X	X	X	
j. Emergence of relevant and actionable community action plans ການສ້າງແຜນປະຕິບັດຂອງຊຸມຊົນທີ່ກ່ຽວຂ້ອງ ແລະ ສາມາດປະຕິບັດຕົວຈິງໄດ້	X	X	X	X	X	X	
k. Observed / anticipated changes in service utilisation ການສັງເກດ / ການຄາດວ່າຈະມີການປ່ຽນແປງໃນການນໍາໃຊ້ການບໍລິການ	X	X	X	X	X	X	

Theme and sub-theme<br>**ຫົວຂໍ້ ແລະ ຫົວຂໍ້ຍ່ອຍ**	Respondent type<br>**ປະເພດຂອງຜູ້ຕອບແບບສອບຖາມ**						
	Workshop facilitator / CONNECT team	Village representative	Village leader	Healthcare staff	Local & district govern. stakeholder	Provincial & central governm. stakeholder	Other stakeholder groups
l. Respectful service provision of health centre staff ການໃຫ້ຄວາມເຄົາລົບໃນການບໍລິການຂອງພະນັກງານແພດ	X	X	X	X	X	X	
m. Quality service provision of health centre staff ຄຸນນພາບດ້ານການໃຫ້ບໍລິການຂອງພະນັກງານແພດ	X	X	X	X	X	X	
n. Actual adaptation of healthcare delivery / access in short and medium term ການປັບໂຕຂອງການນຳສົ່ງການຮັກສາສຸຂະພາບ/ການເຂົ້າເຖິງໃນໄລຍະສັ້ນ ແລະ ໄລຍະກາງ	X	X	X	X	X	X	
4. Efficiency **ປະສິດທິພາບ**<br>*Guiding question: Was CONNECT a good use of resources? ຄຳຖາມແນະນຳ: ໂຄງການ CONNECT ເປັນໂຄງການທີ່ເປັນຊັບພະຍາກອນທີ່ດີ ຫຼື ບໍ່?*							
a. Participant interpretations of CONNECT workshops / activities ການອະທິບາຍ/ຕີຄວາມໝາຍຂອງຜູ້ເຂົ້າຮ່ວມທີ່ໄດ້ເຂົ້າຮ່ວມເວີກຊ໋ອບ ແລະ ກິດຈະກຳຂອງ CONNECT	X	X	X	X	X		
i. Different or similar to other workshops? ມີຄວາມແຕກຕ່າງ ແລະ ຄ້າຍຄືກັນກັບເວີກຊ໋ອບອື່ນໆບໍ່?							
ii. Experience of learning and achievement? What concretely? ປະສົບການໃນການຮຽນຮູ້ ແລະ ຄວາມສຳເລັດ? ສິ່ງທີ່ເປັນຮູບປະທຳມີອັນໃດແນ່?							
iii. Likes / dislikes / observations / suggested improvement ມັກ/ບໍ່ມັກ/ການສັງເກດ/ການແນະນຳການປັບປຸງ							
iv. Sense of success / failure? ຄວາມຮູ້ສຶກຂອງການປະສົບຜົນສຳເລັດ/ບໍ່ສຳເລັດ ແມ່ນຫຍັງ?							
v. Insights / learning (e.g. about community, healthcare staff) ຄວາມເຂົ້າໃຈ / ການຮຽນຮູ້ (ເຊັ່ນ: ກ່ຽວກັບຊຸມຊົນ, ພະນັກງານແພດ)							
b. Experience of workshop implementation and dynamics (successes, challenges, lessons learned) ປະສົບການ ແລະ ຂະບວນການໃນການຈັດຕັ້ງປະຕິບັດເວີກຊ໋ອບ (ຜົນສຳເລັດ, ສິ່ງທ້າທາຍ ແລະ ການຖອດຖອນບົດຮຽນ)	X	X	X	X	X		
c. Experience of planning and learning process surrounding the workshops ປະສົບການໃນການວາງແຜນ ແລະ ຂະບວນການໃນການຮຽນຮູ້ອ້ອມຮອບເວີກຊ໋ອບ	X		X		X		
d. Resource use (operational, allocative) in terms of time, staff, money, political capital, incl.: “Is this the best use of these resources?” ການນຳໃຊ້ຊັບພະຍາກອນ (ການດຳເນີນງານ, ການຈັດສັນ) ໃນແງ່ຂອງເວລາ, ພະນັກງານ, ເງິນ, ທຶນທາງດ້ານການເມືອງ, ລວມທັງ: "ນີ້ແມ່ນການນຳໃຊ້ທີ່ດີທີ່ສຸດຂອງຊັບພະຍາກອນເຫຼົ່ານີ້ບໍ?"	X				X	X	X
5. Impact **ຜົນກະທົບ**<br>*Guiding question: What are the wider consequences of CONNECT beyond its immediate objectives? ຄຳຖາມແນະນຳ: ອັນໃດເປັນຜົນສະທ້ອນທີ່ໄດ້ຮັບຂອງ CONNECT ທີ່ຂອບເຂດກວ້າງກວ່າວັດຖຸປະສົງຂອງໂຄງການເອງ?*							
a. Change and expansion of community-led health initiatives ການປ່ຽນແປງ ແລະ ການຂະຫຍາຍຕໍ່ໃນການລິເລີ່ມດ້ານສາທາລະນະສຸກທີ່ນຳໂດຍຊຸມຊົນ		X	X	X	X	X	

Theme and sub-theme<br>**ຫົວຂໍ້ ແລະ ຫົວຂໍ້ຍ່ອຍ**	Respondent type<br>**ປະເພດຂອງຜູ້ຕອບແບບສອບຖາມ**						
	Workshop facilitator / CONNECT team	Village representative	Village leader	Healthcare staff	Local & district govern. stakeholder	Provincial & central governm. stakeholder	Other stakeholder groups
b. (Anticipated) implications for long-term health sector governance and outcomes (ຄາດວ່າຈະມີ) ຜົນກະທົບຕໍ່ການຄຸ້ມຄອງຂະແໜງສາທາລະນະສຸກໃນໄລຍະຍາວ ແລະ ຜົນໄດ້ຮັບ					X	X	X
c. Evolving sentiment on the role of "communities" in policy ການພັດທະນາຄວາມຮູ້ສຶກໃນບົດບາດຂອງ "ຊຸມຊົນ" ທີ່ມີຕໍ່ນະໂຍບາຍ			X	X	X	X	
d. Unintended consequences ຜົນສະທ້ອນທີ່ບໍ່ຄາດໝາຍ							
i. Expansion of non-health community-led initiatives (i.e. inspiration) ການຂະຫຍາຍຄວາມຄິດລິເລີ່ມທີ່ນໍາໂດຍຊຸມຊົນເຊິ່ງບໍ່ແມ່ນດ້ານສາທາລະນະສຸກ (ເຊັ່ນ: ແຮງບັນດານໃຈ)		X	X	X	X		
ii. Spread of workshop lessons learned across broader community ການເຜີຍແຜ່ບົດຮຽນຂອງເວີກໃນຂອບເຂດທົ່ວຊຸມຊົນ		X	X	X	X		
iii. Biased distribution of workshop activities among higher-educated / similar ethnic community groups ການສະແດງຄວາມຄິດແບບມີອະຄະຕິຕໍ່ກິດຈະກໍາໃນເວີກຊ໋ອບລະຫວ່າງຜູ້ທີ່ມີລະດັບການສຶກສາໃນລະດັບສູງກວ່າ/ກຸ່ມຊົນເຜົ່າທີ່ມີຄວາມຄ້າຍຄືກັນ		X	X	X	X		
iv. Increased social cohesion in community, and thus growing support for healthcare access ເພີ່ມທະວີການຮ່ວມມືທາງສັງຄົມໃນຊຸມຊົນ, ແລະ ການຂະຫຍາຍຕົວດ້ານການສະໜັບສະໜູນການເຂົ້າເຖິງການເບິ່ງແຍງສຸຂະພາບ		X	X	X	X		
v. Growing community interest in healthcare topics, greater health literacy, and higher preventive & acute healthcare service utilisation ການຂະຫຍາຍຕົວຄວາມສົນໃຈຂອງຊຸມຊົນໃນຫົວຂໍ້ການເບິ່ງແຍງຮັກສາສຸຂະພາບ, ການຮູ້ຂໍ້ມູນດ້ານສາທາລະນະສຸກໃຫ້ຫຼາຍຂຶ້ນ, ແລະ ການໃຊ້ບໍລິການການຮັກສາສຸຂະພາບທີ່ສູງຂຶ້ນ		X	X	X	X		
vi. Increased healthcare provision from unregulated healthcare providers (driven by feeling of empowerment – e.g. overusing antiviral medicine from unregulated sources) ການສະໜອງເບິ່ງແຍງສຸຂະພາບທີ່ເພີ່ມຂຶ້ນຈາກຜູ້ໃຫ້ບໍລິການດ້ານສຸຂະພາບທີ່ບໍ່ມີການຄວບຄຸມ (ຍ້ອນຄວາມຮູ້ສຶກຂອງການສ້າງຄວາມເຂັ້ມແຂງ - ຕົວຢ່າງເຊັ່ນການໃຊ້ຢາຕ້ານໄວຣັສຫຼາຍເກີນໄປຈາກແຫຼ່ງທີ່ບໍ່ມີການຄວບຄຸມ)		X	X	X	X		
vii. Loss of trust disempowers, marginalizes, impoverishes private and informal healthcare providers		X	X	X	X		
viii. Inequitable barriers to behavioural change within communities for CONNECT workshops ອຸປະສັກທີ່ບໍ່ສະເໝີພາບຕໍ່ກັບການປ່ຽນແປງພຶດຕິກໍາພາຍໃນຊຸມຊົນສໍາລັບເວີກຊ໋ອບຂອງ CONNECT		X	X	X	X		
ix. Unforeseen interpretations of workshop / training content ການຕີຄວາມໝາຍທີ່ບໍ່ຄາດຄິດຂອງເວີກຊ໋ອບ/ຮູບແບບຂອງການເຝິກອົບຮົມ	X	X	X	X	X		
x. Demand for new range of (healthcare) services that cannot be provided, which frustrates relationships in medium term ຄວາມຕ້ອງການການບໍລິການ (ການເບິ່ງແຍງຮັກສາສຸຂະພາບ) ໃນລະດັບໃໝ່ທີ່ບໍ່ສາມາດສະໜອງໄດ້, ເຊິ່ງເຮັດໃຫ້ຄວາມຜິດຫວັງໃນການພົວພັນໃນໄລຍະກາງ		X	X	X	X		

Theme and sub-theme<br>**ຫົວຂໍ້ ແລະ ຫົວຂໍ້ຍ່ອຍ**	Respondent type<br>**ປະເພດຂອງຜູ້ຕອບແບບສອບຖາມ**						
	Workshop facilitator / CONNECT team	Village representative	Village leader	Healthcare staff	Local & district govern. stakeholder	Provincial & central governm. stakeholder	Other stakeholder groups
6. Sustainability **ຄວາມຍືນຍົງ**<br>*Guiding question: Will CONNECT (continue to) produce net benefits after its expiry? ຄໍາຖາມແນະນໍາ: CONNECT ຈະ (ສືບຕໍ່) ຜົນປະໂຫຍດສຸດທິຫຼັງຈາກໂຄງການໄດ້ສິ້ນສຸດ ຫຼື ບໍ?*							
a. (Anticipated) financial sustainability (funding continuity) (ການຄາດຄະເນ) ຄວາມຍືນຍົງທາງການເງິນ (ການຫາທຶນຢ່າງຕໍ່ເນື່ອງ)					X	X	
b. (Anticipated) operational sustainability (strategy development and integration) (ການຄາດຄະເນ) ຄວາມຍືນຍົງໃນການດໍາເນີນງານ (ການພັດທະນາຍຸດທະສາດ ແລະ ການເຊື່ອມໂຍງ)		X	X	X	X	X	
c. (Anticipated) institutional sustainability (formalizing process into policy, upholding relationships) (ການຄາດຄະເນ) ຄວາມຍືນຍົງຂອງສະຖາບັນ ()					X	X	X
d. Monitoring of CONNECT strategy implementation / adaptations ການຕິດຕາມການປະຕິບັດ / ການປັບຕົວຍຸດທະສາດຂອງ CONNECT	X		X		X	X	
e. Communication and feedback to government levels to enable institutional learning ການສື່ສານ ແລະ ຂໍ້ສະເໜີແນະນໍາໃີ່ນລະດັບລັດຖະບານເພື່ອເຮັດໃຫ້ເກີດການຮຽນຮູ້ພາຍໃນສະຖາບັນ			X	X	X	X	
f. Expectations for independent implementation / continuation of CONNECT ຄວາມຄາດຫວັງສໍາລັບການປະຕິບັດແບບອິດສະຫຼະ/ ການສືບຕໍ່ຂອງໂຄງການ CONNECT	X	X	X	X	X	X	
7. Equity **ຄວາມເທົ່າທຽມ/ເປັນທໍາ**<br>*Guiding question: What are the distributional consequences of CONNECT in its implementation, outcomes, and impacts? ຄໍາຖາມແນະນໍາ: ອັນໃດແມ່ນເຫດຜົນທີ່ນໍາມາຈາກໂຄງການ CONNECT ໃນການນໍາໄປປະຕິບັດ, ຜົນທີ່ໄດ້ຮັບ ແລະ ຜົນກະທົບ?*							
a. General inclusivity of marginalized groups in Lao healthcare ການມີສ່ວນຮ່ວມໂດຍທົ່ວໄປຂອງກຸ່ມຄົນດ້ອຍໂອກາດໃນການເບິ່ງແຍງສຸຂະພາບໃນລາວ		X	X	X	X	X	X
b. Equitable inclusion of CONNECT communities ການຮອບຮອມຄວາມສະເໝີພາບຂອງຊຸມຊົນ CONNECT	X	X	X	X	X		
c. Equitable community engagement in workshops and follow-on work ການມີສ່ວນຮ່ວມຢ່າງເທົ່າທຽມໃນຊຸມຊົນໃນເວີກຊ໋ອບ ແລະ ການຕິດຕາມຜົນຂອງວຽກ	X	X	X	X	X		
d. Equitable distribution of CONNECT outcomes (trust, service utilisation, respectful care) ການແຈກຢາຍຜົນໄດ້ຮັບຂອງໂຄງການ CONNECT ທີ່ເທົ່າທຽມກັນ (ຄວາມໄວ້ວາງໃຈ, ການໃຊ້ບໍລິການ ແລະ ການເບິ່ງແຍງດ້ວຍຄວາມເຄົາລົບ)	X	X	X	X	X		
e. Growing recognition of marginalized and indigenous groups and their specific needs and priorities ການຂະຫຍາຍຕົວການຮັບຮູ້ຂອງກຸ່ມຄົນດ້ອຍໂອກາດ ແລະ ຊົນເຜົ່າພື້ນເມືອງ ແລະ ຄວາມຕ້ອງການສະເພາະ ແລະ ບູລິມະສິດຂອງເຂົາເຈົ້າ	X	X	X	X	X		
8. Other mechanisms (optional / later) **ກົນໄກອື່ນໆ (ທາງເລືອກ / ພາຍຫຼັງ)**<br>*Guiding question: What factors shape the behavioural outcomes of CONNECT? ຄໍາຖາມແນະນໍາ: ປັດໃຈອັນໃດທີ່ສ້າງຜົນປະພຶດຕໍ່ກັບໂຄງການ CONNECT?*							
a. Automatic motivation ແຮງຈູງໃຈແບບອັດຕະໂນມັດ		X	X	X			

Theme and sub-theme<br>ຫົວຂໍ້ ແລະ ຫົວຂໍ້ຍ່ອຍ	Respondent type<br>ປະເພດຂອງຜູ້ຕອບແບບສອບຖາມ<br>Workshop facilitator / CONNECT team	Village representative	Village leader	Healthcare staff	Local & district govern. stakeholder	Provincial & central governm. stakeholder	Other stakeholder groups
i. Development of trust reduces fear of discrimination ການພັດທະນາຄວາມໄວ້ວາງໃຈ ເພື່ອຫຼຸດຜ່ອນຄວາມຢ້ານກົວໃນການແບ່ງແຍກ/ຈຳແນກ							
ii. Intervention creates impetus to break out of habits ການແຊກແຊງສ້າງແຮງກະຕຸ້ນເພື່ອທຳລາຍນິໄສ							
iii. Reframing of COVID reduces stigma and other reactions (affect) ການວາງກອບຂອງພະຍາດໂຄວິດຊ່ວຍຫຼຸດຜ່ອນການໃສ່ຮ້າຍປ້າຍສີ ແລະ ປະຕິກິລິຍາອື່ນໆ (ຜົນກະທົບ)							
iv. CONNECT reshapes community norms, reveals new consensus CONNECT ໄດ້ປັບປ່ຽນມາດຕະຖານຂອງຊຸມຊົນ, ເປີດເຜີຍຄວາມເຫັນດີແບບໃໝ່							
v. Changing trust re-shapes health communication and recommendation networks within the community (messenger) ການປ່ຽນແປງຄວາມໄວ້ວາງໃຈສ້າງຮູບແບບການສື່ສານດ້ານສຸຂະພາບ ແລະ ເຄືອຂ່າຍແນະນຳພາຍໃນຊຸມຊົນ (messenger)							
b. Reflective motivation ການສະທ້ອນຂອງແຮງຈູງໃຈ		X	X	X			
i. Priorities and realities become better known among stakeholders, which is reflected in better-tailored initiatives ບູລິມະສິດ ແລະ ຄວາມຈິງທີ່ກາຍເປັນທີ່ຮູ້ຈັກກັນດີຂຶ້ນລະຫວ່າງບັນດາພາກສ່ວນກ່ຽວຂ້ອງ, ເຊິ່ງສະແດງໃຫ້ເຫັນໃນຄວາມຄິດລິເລີ່ມທີ່ສາມາດປັບປຸງໃຫ້ເໝາະສົມໄດ້ຍິ່ງຂຶ້ນ.							
ii. Workshop content provides concrete learning opportunity ເນື້ອໃນຂອງເວີກຊ້ອບໄດ້ໃຫ້ໂອກາດໃນການຮຽນຮູ້ທີ່ຊັດເຈນ							
iii. Identification of marginalized groups for service targeting ການກຳນົດກຸ່ມເປົ້າໝາຍສຳລັບການກຳນົດເປົ້າໝາຍໃນການບໍລິການ							
iv. Community has greater stake in healthcare through “ownership” ຊຸມຊົນມີສ່ວນຮ່ວມຫຼາຍກວ່າໃນການເບິ່ງແຍງສຸຂະພາບໂດຍຜ່ານ "ຄວາມເປັນເຈົ້າຂອງ"							
v. Observable reduction in disrespectful & patronizing encounters ການພົບປະເຊິ່ງໜ້າທີ່ບໍ່ສຸພາບ ແລະ ການອຸປະຖຳທີ່ຫຼຸດລົງທີ່ສັງເກດໄດ້ຢ່າງຊັດເຈນ							
vi. Assurance of service quality ການຮັບປະກັນຄຸນນະພາບໃນການບໍລິການ							
c. Social opportunity ໂອກາດທາງດ້ານສັງຄົມ		X	X	X			
Strengthened community relationships (“social capital”)<br>i. ຮັດແໜ້ນຄວາມສຳພັນໃນຊຸມຊົນ (ທຶນທາງສັງຄົມ)							
ii. Ethnically inclusive communication ການສື່ສານທີ່ກອມລວມກຸ່ມຊົນເຜົ່າ							
iii. Improved inter-ethnic and -stakeholder trust ການປັບປຸງຄວາມໄວ້ວາງໃຈລະຫວ່າງຊົນເຜົ່າ ແລະ ຜູ້ມີສ່ວນຮ່ວມ							
iv. Tailoring of services reduces social (ethnic) barriers to care ການບໍລິການຕັດຫຍິບຊ່ວຍຫຼຸດຜ່ອນອຸປະສັກທາງດ້ານສັງຄົມ (ຊົນເຜົ່າ) ທີ່ມີຕໍ່ການເບິ່ງແຍງສຸຂະພາບ							
d. Physical opportunity ໂອກາດທາງດ້ານຮ່າງກາຍ		X	X	X			

Theme and sub-theme<br>**ຫົວຂໍ້ ແລະ ຫົວຂໍ້ຍ່ອຍ**	Respondent type<br>**ປະເພດຂອງຜູ້ຕອບແບບສອບຖາມ**						
	Workshop facilitator / CONNECT team	Village representative	Village leader	Healthcare staff	Local & district govern. stakeholder	Provincial & central govern. stakeholder	Other stakeholder groups
i. Tailoring reduces physical (transport, finance) barriers to care ການຕັດຫຍິບສາມາດຊ່ວຍຫຼຸດຜ່ອນອຸປະສັກໃນການເບິ່ງແຍງໃນດ້ານຮ່າງກາຍ (ດ້ານການຂົນສົ່ງ, ດ້ານການເງິນ)							
e. Physical capability ຄວາມສາມາດທາງດ້ານຮ່າງກາຍ		X	X	X			
i. Services cater better to disabled population sub-groups ການບໍລິການສາມາດຕອບສະໜອງໄດ້ດີກວ່າຕໍ່ປະຊາກອນກຸ່ມຍ່ອຍທີ່ມີຄວາມພິການ							
ii. Identification of high-risk COVID sub-populations ການກຳນົດກຸ່ມຍ່ອຍຂອງປະຊາກອນທີ່ມີຄວາມສ່ຽງສູງຕໍ່ພະຍາດໂຄວິດ							
f. Psychological capability ຄວາມສາມາດທາງດ້ານຈິດໃຈ		X	X	X			
i. Knowledge and training on respectful care, clinical standards ຄວາມຮູ້ ແລະ ການເຝິກອົບຮົມກ່ຽວກັບການເບິ່ງແຍງເອົາໃຈໃສ່, ມາດຕະຖານຂອງຄຣີນິກ							
ii. Heuristic short-cuts to solutions and practices endorsed by the public health system of Lao PDR (i.e. villagers might no longer search around for solutions) (ເຊັ່ນ: ປະຊາຊົນອາດບໍ່ຄິດຫາວິທີແກ້ໄຂບັນຫາອີກຕໍ່ໄປ)							
9. Final question **ຄຳຖາມສຸດທ້າຍ**							
a. Any suggestions or recommendations for the CONNECT initiative / Lao health policy? ທ່ານມີຂໍ້ສະເໜີ ຫຼື ຄຳແນະນຳເພີ່ມເຕີມ ຫຼື ບໍ່ ສຳລັບໂຄງການຂອງ CONNECT/ນະໂຍບາຍດ້ານສຸຂະພາບຂອງລາວ?	X	X	X	X	X	X	X

This is the end of our interview. Thank you so much for sharing your views with me. Your responses have been very helpful. ການສໍາພາດນີ້ສິ້ນສຸດແລ້ວ ຄໍາຕອບທີ່ທ່ານໃຫ້ກັບເຮົາຈະເປັນປະໂຫຍດກັບການຄົ້ນຄ້ວາຂອງເຮົາຫຼາຍ ຂອບໃຈສໍາລັບຄໍາຄິດເຫັນທີ່ທ່ານແບ່ງປັນກັບເຮົາ
